# Maternal plasma cortisol’s effect on offspring birth weight: a Mendelian Randomisation study

**DOI:** 10.1186/s12884-024-06250-3

**Published:** 2024-01-15

**Authors:** WD Thompson, RM Reynolds, RN Beaumont, NM Warrington, J Tyrrell, AR Wood, DM Evans, TJ McDonald, AH Hattersley, RM Freathy, DA Lawlor, MC Borges

**Affiliations:** 1 Department of Clinical and Biomedical Sciences, Faculty of Health and Life Sciences, University of Exeter, Exeter, UK; 2 Academic Rheumatology, Clinical Sciences Building, Nottingham City Hospital, Hucknall Road, Nottingham, NG5 1PB United Kingdom; 3 Centre for Cardiovascular Science, The Queens Medical Research Institute, University of Edinburgh, Edinburgh, UK; 4 Institute for Molecular Bioscience, University of Queensland, Brisbane, Australia; 5 K.G. Jebsen Center for Genetic Epidemiology, Department of Public Health and Nursing, NTNU, Norwegian University of Science and Technology, Trondheim, Norway; 6 Frazer Institute, University of Queensland, Brisbane, Australia; 7 MRC Integrative Epidemiology Unit, University of Bristol, Bristol, UK; 8 Academic Department of Blood Sciences, Royal Devon and Exeter NHS Foundation Trust, Exeter, UK; 9 Population Health, Bristol Medical School, University of Bristol, Bristol, UK; 10 Bristol NIHR Biomedical Research Centre, Bristol, UK

**Keywords:** UK Biobank, EFSOCH, Cortisol, Birth weight, Mendelian Randomization

## Abstract

**Background:**

Observational studies and randomized controlled trials have found evidence that higher maternal circulating cortisol levels in pregnancy are associated with lower offspring birth weight. However, it is possible that the observational associations are due to residual confounding.

**Methods:**

We performed two-sample Mendelian Randomisation (MR) using a single genetic variant (rs9989237) associated with morning plasma cortisol (GWAS; sample 1; *N* = 25,314). The association between this maternal genetic variant and offspring birth weight, adjusted for fetal genotype, was obtained from the published EGG Consortium and UK Biobank meta-analysis (GWAS; sample 2; *N* = up to 406,063) and a Wald ratio was used to estimate the causal effect. We also performed an alternative analysis using all GWAS reported cortisol variants that takes account of linkage disequilibrium. We also tested the genetic variant’s effect on pregnancy cortisol and performed PheWas to search for potential pleiotropic effects.

**Results:**

The estimated effect of maternal circulating cortisol on birth weight was a 50 gram (95% CI, -109 to 10) lower birth weight per 1 SD higher log-transformed maternal circulating cortisol levels, using a single variant. The alternative analysis gave similar results (-33 grams (95% CI, -77 to 11)). The effect of the cortisol variant on pregnancy cortisol was 2-fold weaker than in the original GWAS, and evidence was found of pleiotropy.

**Conclusions:**

Our findings provide some evidence that higher maternal morning plasma cortisol causes lower birth weight. Identification of more independent genetic instruments for morning plasma cortisol are necessary to explore the potential bias identified.

**Supplementary Information:**

The online version contains supplementary material available at 10.1186/s12884-024-06250-3.

## Background

Variation in human birth weight is associated with adverse perinatal health outcomes as well as long term health outcomes [1]. In particular, lower than average birth weight is associated with higher neonatal mortality and a higher risk of cardiovascular disease [2], type 2 diabetes [3] and hypertension [4] in adulthood. Understanding mechanisms that influence variation in birth weight could help identify targets for intervention to ensure healthy birth weight.

Experimental studies in animal models and observational studies in humans have demonstrated links between higher fetal glucocorticoid exposure and lower birth weight [5]. Higher maternal cortisol levels are one potential source of increased fetal glucocorticoid exposure, with evidence of higher levels of both maternal plasma [6] and salivary [7] cortisol being associated with lower birth weight infants. Infants exposed to antenatal corticosteroids in a secondary analyses of a randomized controlled trial (RCT) of women at risk of preterm birth also have lower birth weight compared to those randomised to placebo, although this was in part related to also having a shorter gestation [8].

There are challenges to assessing the effect of maternal cortisol levels on offspring birth weight. There are several maternal characteristics that can confound the relationship between maternal cortisol and offspring birth weight, such as maternal smoking and body mass index (BMI) [9], which can be difficult or even impossible to fully account for in conventional observational studies. Also, whilst the RCT evidence was from a large and well conducted study and therefore unlikely to be biased by confounding, it was limited to women at risk of preterm birth only. Furthermore, it was not a direct test of the effect of maternal cortisol on birth weight and the lower birth weight in those randomized to corticosteroids was driven in large part by reduced gestational duration [8]. Mendelian Randomization (MR), uses genetic variants to probe the effect of modifiable exposures (e.g. maternal cortisol levels) on health outcomes (e.g. offspring birth weight) [10]. Given that genetic variation is randomised at conception, MR is less susceptible to being biased by variables that are observationally correlated with the exposure variable but independently impact the outcome via a mechanism independent of the mechanism being tested.

We hypothesized that higher maternal plasma cortisol causes lower offspring birth weight and used MR to test this hypothesis. We used the most recent Genome Wide Association Study (GWAS) of fasting plasma cortisol levels [11] as the source of genetic variant associations with the exposure, and we used the GWAS of offspring birth weight in the Early Growth Genetics (EGG) Consortium and UK Biobank [12] to obtain estimates of maternal genetic effects on birth weight conditional on the fetal genotype. To investigate the plausibility of instrumental variable assumptions, we also tested the genetic variant’s association with cortisol in pregnancy in a European-ancestry birth cohort [13] and searched for potential sources of horizontal pleiotropy using an online database [14, 15].

## Methods

**Fig. 1:**
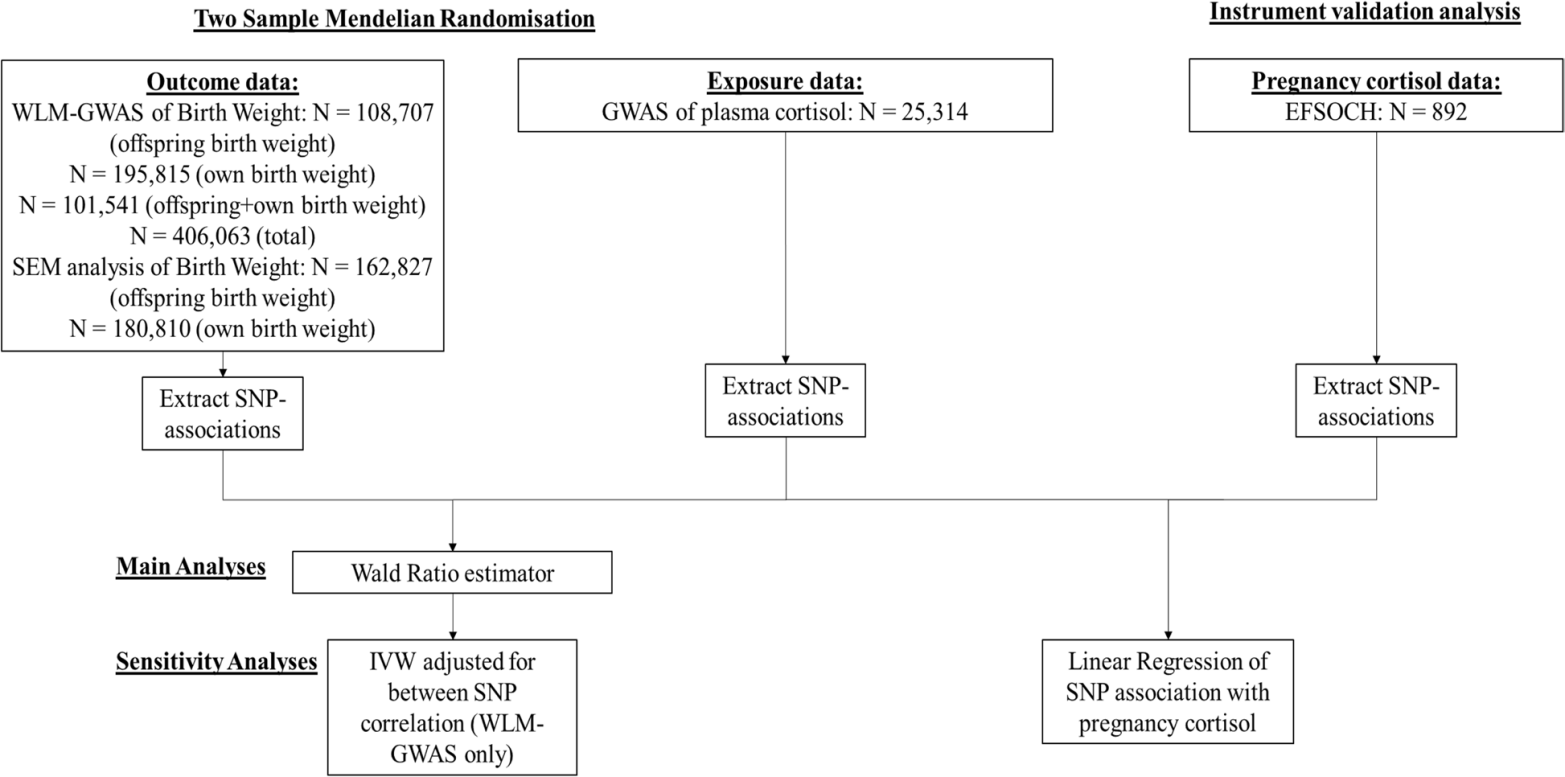
Diagram summarising the key data sources and analysis steps for this study

We used two-sample MR to estimate the causal effect of maternal plasma cortisol on offspring birth weight [16]. This method involves using estimates of the single nucleotide polymorphism (SNP)-exposure associations (using SNPs that are robustly associated with the exposure, in this case plasma cortisol) as well as using SNP-outcome associations extracted from a pre-existing data set (in this case offspring birth weight). For each SNP, the SNP-outcome association is divided by the SNP-exposure association. Normally, these ratios would be pooled to give an estimate of the causative effect of the exposure on an outcome. For this study we were limited by the fact that only one genome-wide significant locus for plasma cortisol has been identified. The study design and different sources used are summarised in Fig. 1.

### Data sources

A summary of all the cohorts contributing to the GWAS summary statistics used in this study can be found in Table 1.

**Table 1:** Summary of studies contributing to a) the circulating cortisol GWAS (CORNET), b) the maternal effects on pregnancy GWAS (EGG) and c) the observational pregnancy circulating cortisol (EFSOCH)

a)	Study	Country	N	Age, years (SD)	Cortisol, nmol/l (SD)	Sampling time
	ORCADES	UK	1974	53.5 (15.7)	765 (315)	0830-1030
	CROATIA-Korcula	Croatia	898	56.2 (13.9)	698 (207)	0800-0900
	CROATIA-Split	Croatia	496	45.0 (14.7)	979 (404)	0730-0900
	CROATIA-Vis	Croatia	892	56.4 (15.5)	622 (230)	0730-0900
	Rotterdam Study	Netherlands	6497	63.3 (9.6)	359 (115)	0800-1100
	HBCS1934-44	Finland	451	60.61 (2.80)	393 (120)	0750-1055
	NFBC1966	Finland	1324	31.1 (0.3)	380 (160)	0800-1100
	ALSPAC	UK	1567	15.43 (0.26)	486 (174)	0800-1057
	PIVUS	Sweden	919	70.2 (0.17)	386 (125)	0800-1000
	PREVEND	Netherlands	1151	49.4 (13.0)	442 (201)	0800-1100
	ET2DS	UK	1048	67.9 (4.2)	731 (190)	0800-0830
	Raine Study	Australia	860	17.1 (0.29)	614 (235)	Awakening (before 1000)
	MrOS Sweden	Sweden	969	75.3 (3.2)	487 (133)	0700-1000
	VIKING	UK	2073	49.9 (15.2)	292 (170)	0800-1030
	SHIP	Germany	910	49.8 (13.8)	*	Before 1300
	TwinsUK	UK	5654	53.3 (13.8)	*	0800-1200
	KORA	Germany	1651	60.92 (8.7)	*	NA
b)	Study	Country	N	Age, years (SD)	Birthweight, g (SD)	Gestational age, weeks (IQR)
	UK Biobank	UK	190,406	25.3 (4.5)	3227 (477)	NA
	B58C-WTCCC	UK	858	26.2 (5.2)	3325 (483)	40 (40–41)
	B58C-T1DGC	UK	836	26.1 (5.4)	3379 (469)	40 (40–41)
	DNBC-GOYA	Denmark	1805	29.2 (4.2)	3643 (495)	40 (39–41)
	DNBC-PTB-CONTROL	Denmark	1656	29.9 (4.2)	3595 (497)	40 (39–40)
	MoBa-2008	Norway	650	28.5 (3.3)	3679 (430)	40 (0.9)
	NFBC1966	Finland	2035	26.5 (3.7)	3525 (461)	40 (2)
	NTR	Netherlands	707	27.1 (3.7)	3469 (529)	40 (38–42)
	QIMR	Australia	892	24.5 (4.0)	3344 (532)	NA
	TWINSUK	UK	1603	NA	NA	NA
	ALSPAC	UK	6,686	28.0 (5.0)	3468 (475)	40 (40–41)
	HAPO	USA	1280	31.5 (5.3)	3557 (517)	40 (1.7)
	EFSOCH	UK	855	30.5 (5.9)	3506 (472)	40 (37–43)
c)	Study	Country	N	Age, years (SD)	Cortisol, nmol/l (SD)	Sampling time
	EFSOCH	UK	892	30.4 (5.3)	1010 (234)	0900 (within 60 minutes)

^a^Apart from EFSOCH [13] circulating cortisol (in pregnancy), the data comes from Crawford et al 2021 [11] and Warrington et al 2019 [12]

^b^This table only shows the studies that contributed maternal genotype and offspring birthweight data (*n* = 210,267) to the final WLM-adjusted GWAS of offspring birthweight (*n* = 406,063). More information regarding offspring genotype and own birthweight data can be found in Warrington et al. 2019

^c^*ORCADES* Orkney Complex Disease Study, *HBCS1934-44* Helsinki Birth Cohort Study 1934-1944, *NFBC1966* the Northern Finland 1966 Birth Cohort, *ALSPAC* Avon Longitudinal Study of Parents and Children; *PIVUS* Prospective Investigation of the Vasculature in Uppsala Seniors, *PREVEND* Prevention of Renal and Vascular End-stage Disease, *ET2D2* Edinburgh Type 2 Diabetes Study, *B58C-T1DGC* British 1958 Birth Cohort – Type 1 Diabetes Genetics Consortium, *MrOS Sweden* Osteoporotic Fractures in Men-Sweden, *KORA* Cooperative Health Research in the Augsburg Region, *SHIP* Study of Health in Pomerania, *VIKING* Viking Health Study-Shetland, *B58C-WTCCC* British 1958 Birth Cohort – Wellcome Trust Case Control Consortium, *DNBC-GOYA* Danish National Birth Cohort – Genetics of Overweight Young Adults, *DNBC-PTB-CONTROL* Danish National Birth Cohort – Preterm Birth-Control Mothers, *MoBa-2008* the Norwegian Mother and Baby Cohort, 2008, *NTR* Netherlands Twin Registry, *QIMR* Queensland Institute of Medical Research, *HAPO* Hyperglycaemia and Adverse Pregnancy Outcome Study, *NA* Not applicable;

#### Genetic associations with plasma cortisol

SNPs associated with circulating cortisol were identified from the most recent GWAS (*N*=25,314) [11]. In total, 17 cohorts contributed to the GWAS, and usually measured circulating cortisol levels before 12pm (range 7am to 1pm) [11]. in which four SNPs within one locus (i.e. the *SERPINA6/SERPINA1* locus) were associated with fasting plasma cortisol at genome wide significance (*p*-value ≤ 5e^-8^) [11]. These four SNPs are in partial linkage disequilibrium (LD) with one another and we selected the SNP most strongly associated with circulating cortisol, rs9989237, as the genetic instrument for our main MR analysis [11]. Details of the identified SNPs are found in Additional file 1 (Additional Table 1).

#### Genetic associations with birth weight

For our second sample we used the latest maternal GWAS of offspring birth weight from the Early Growth Genetics (EGG) meta-analysis. A total of 406,063 participants contributed to the weighted linear model analyses (WLM, see below) to estimate maternal effects conditional on offspring genotype, and offspring effects conditional on maternal genotype (see Additional file 1 (Methods)). Of these participants, 101,541 were UK Biobank participants who reported their own birth weight and birth weight of their first child, 195,815 were UK Biobank and EGG participants with own birth weight data, and 108,707 were UK Biobank and EGG participants with offspring birth weight data [12].In the UK Biobank and EGG meta-analysis, birth weight was standardized within each of the cohorts so that birth weight in our analyses is measured in SD units and our results were initially the difference in mean birth weight in SD units. We converted these to a difference in mean birth weight in grams by using the SD of birth weight from an earlier EGG paper (1 SD of birthweight = 484g) [17].

#### Genetic associations with maternal pregnancy cortisol

Cortisol levels in 892 mothers in the EFSOCH cohort [13] were assayed at 28 weeks gestation (Additional file 1 (Methods)). EFSOCH mothers were genotyped in three batches (one in Exeter, two in Bristol) using the Illumina Infinium HumanCoreExome-24 array, and when multiple genotyping batches are used for the same sample, bias can occur due to random differences between those participants assigned to one batch versus another (i.e., a batch effect) [18]. The association between the GWAS identified SNP and pregnancy cortisol in EFSOCH was adjusted for the genotyping chip to guard against batch effects.

### Data analyses

Our main analysis was to estimate the effect of maternal plasma cortisol on offspring birth weight in the UK Biobank and EGG meta-analysis. In addition to this, we undertook analyses to assess instrumental variable assumptions, specifically to determine the strength of the cortisol instruments and to explore the possibility of horizontal pleiotropy in the cortisol instrument.

#### Adjusting for the fetal genotype

To avoid violating the third assumption of MR (i.e. that a genetic instrument affects the outcome only via the associated exposure) due to fetal genetic effects [10], we adjusted for the fetal genotype. For the main analysis, to ensure our analyses considered only the effect of the maternal genotype, and not the correlated fetal genotype, we used SNP-birth weight associations that had been adjusted for fetal genotype using a weighted linear model (WLM) [12]. The WLM is a method that was developed to combine data from disparate study designs to estimate conditional maternal and fetal genetic effects, similar to conditional genetic association analysis in genotyped mother-child pairs (see Additional file 1 (Methods) and references [12, 19]). To verify the WLM-adjusted summary statistics, we also applied the SEM method to obtain the SNP maternal effect on offspring birth weight, adjusted for the fetal genotype using UK Biobank participants (own birth weight *N* = 186,810; offspring birth weight *N* = 162,827) and repeated the main MR analysis to check we obtained similar results.

#### Main MR analyses

We performed two-sample MR using the Wald ratio estimator [20], which was calculated by dividing the SNP’s effect on birth weight by the same SNP’s effect on circulating cortisol. Standard errors were calculated by dividing the standard error of the SNP’s effect on birth weight by the SNP’s effect on cortisol. This was done using SNP-outcome estimates from both the main WLM analysis and from our own SEM analysis. The resulting effect estimates from our MR analyses are reported per 1 SD of log-transformed plasma cortisol levels [11].

#### IVW analysis adjusting for between SNP correlations

To maximise power, we performed an additional MR analysis incorporating the four SNPs in partial LD at the *SERPINA6/SERPINA1* locus, as reported by Crawford et al [11]. Given those SNPs were partially correlated, we used a modified inverse variance weighted (IVW) analyses which accounts for the correlation across genetic instruments using the TwoSampleMR [21] and MendelianRandomisation [22] R packages and a correlation matrix of variants obtained from the 1000 genomes EUR reference panel via TwoSampleMR [21]. The correlation matrix of the R values used for this analysis is presented in Additional file 1 (Additional Table 2).

#### Testing cortisol instrument strength

An MR assumption is that the genetic instruments are robustly associated with the exposure. In two-sample MR, as undertaken here, weak instrument bias is expected to bias estimates towards the null in the absence of sample overlap. To test the strength of the genetic instruments for cortisol, we calculated the R^2^ and F-Statistic for all four SNP-cortisol associations reported in the GWAS (see Additional file 1 (Methods) for further details).

#### Testing cortisol instruments relevance to pregnancy

The cortisol GWAS was performed in a non-pregnant, mixed sex population, therefore it is possible that the instruments detected do not predict variations in circulating cortisol during pregnancy, or if they do, this is with a different magnitude to what we assume when using the GWAS result. We therefore compared the association between SNP rs9989237 and fasting plasma cortisol levels measured in pregnancy in the EFSOCH cohort with the same results from the original GWAS (see Additional file 1 (Methods) for further details).

#### Exploring the possibility of horizontal pleiotropy in the cortisol instrument

Another core MR assumption is that any effect of the genetic instrument on the outcome is fully mediated by the exposure. If this assumption is violated, the genetic instrument is considered invalid and MR estimates could be biased. Numerous MR methods have been developed that are robust to the presence of invalid instruments e.g. MR-Egger [23], weighted-median [24], Radial MR [25]. However, these methods typically require that multiple genetic instruments from different loci are available for a particular exposure. Given that only one independent SNP was available for our analyses, we explored the plausibility of the assumption of no invalid instruments by assessing the specificity of our genetic instrument in a phenome-wide association (PheWAS) scan using data from the MR-Base platform [14, 15], which has data from a wide range of GWAS that can be easily downloaded via R. To perform the scan, we downloaded every tested association between rs9989237 and an available GWAS variable using the “ieu-gwas-r” package [14], by specifying the *p*-value threshold at 1. This gave us 19,269 different variables in total. Though all of the variables associated with rs9989237 could result in pleiotropy, we decided to focus our attention on those variables whose *p*-value passed a Bonferroni threshold of 2.6e^-06^.

## Results

### Main results and sensitivity analyses

**Fig. 2:**
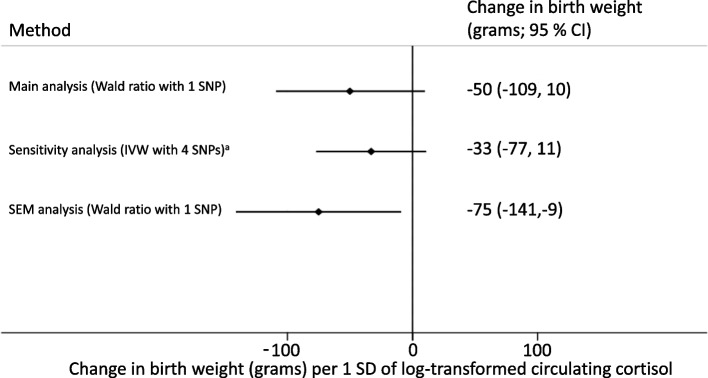
Mendelian Randomisation causative effect estimates for maternal plasma cortisol on mean birth weight. a) The SNPs used in the sensitivity analyses are correlated with the SNP used in the main analyses and each other. We used a form of IVW analyses that adjusts for between SNP correlations. b) IVW, Inverse Variance Weighted; SEM, Structural Equation Model

The estimated effect of maternal circulating cortisol was a 50 (95% CI, -109 to 10) grams lower offspring birth weight per 1 SD higher log-transformed maternal circulating cortisol levels. When using all four SNPs in IVW analysis adjusted for correlation between SNPs, the result was similar (-33 (95% CI, -77 to 11). Using the SEM to adjust for the fetal genotype gave similar results (-75 (95% CI, -141 to -9)). All effect estimates are shown in Fig. 2.

### SNP validation

#### Instrument strength and relevance in pregnancy

Using the data from the largest available GWAS, we estimated that the SNP used in the main analyses (rs9989237) explained ~0.2% of the variation in cortisol and had an F-statistic of 62. The R^2^ values and F-statistics for the other SNPs are shown in Table 2.

**Table 2:** R^2^ and F-statistic results for the genetic variants that were genome wide significant in the original genome wide association study

Genetic variant (SNP) ID	Number of-participants	Minor allele frequency	Per allele difference (SDs of log-transformed units) in plasma cortisol (95% CI)	R^2^	F-Statistic
rs11620763	25314	0.19	0.09 (0.06 to 0.11)	0.0023	57.49
rs2736898	25314	0.49	0.06 (0.04 to 0.07)	0.0017	43.37
rs7146221	25314	0.45	0.05 (0.03 to 0.07)	0.0013	31.87
rs9989237^a^	25314	0.21	0.09 (0.07 to 0.10)	0.0024	61.83

^a^SNP used in main analysis

^b^The R^2^ and F-statistics for rs11620763, rs2736898 and rs7146221 may be under or overestimated due to linkage disequilibrium with rs9989237

**Fig. 3:**
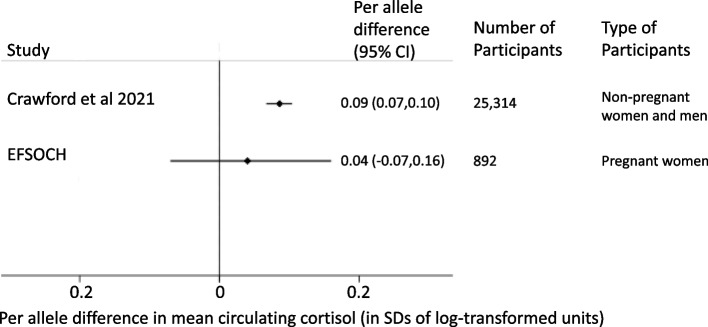
Main SNPs effect on cortisol levels in primary GWAS and EFSOCH pregnancy sample

In the EFSOCH study [13], the mean value of women’s fasting plasma cortisol was 1,010 nmol/l (SD; 233 nmol/l) or 3 log-transformed nmol/l (SD; 0.1 log-transformed nmol/l). The SNP used in our main analyses had a considerably (2-fold) weaker association with women’s fasting plasma cortisol levels in pregnancy than seen in the main GWAS of non-pregnant women and men (0.04 (95% CI, -0.07 to 0.16) vs 0.09 (95% CI, 0.07 to 0.10)), though given the small sample size the estimate was imprecise with very wide confidence intervals that included the GWAS point estimate and the null (see Fig. 3).

#### Possibility of the instrument influencing birth weight through horizontal pleiotropy

In total, 11 variables were associated at Bonferroni significance with rs9989237, and a further 1,516 variables were nominally associated with rs9989237. These associations with the cortisol increasing variant included higher levels of *SERPINA1* (beta = 0.123, *p* = 4.09e^-18^), 39S ribosomal protein L33 (beta = 0.252, *p* = 2.82e^-17^), PH and SEC7 domain-containing protein 1 (beta = 0.200, *p* = 2.24e^-11^) and Histidine (beta = 0.026, *p* = 3e^-07^), as well as lower levels of Albumin (beta = -0.034, *p* = 1.18e^-28^), Synaptosomal-associated protein 25 (beta = -0.18, *p* = 1.78e^-09^) and sex-hormone binding globulin (SHBG) both with (beta = -0.005, *p* = 3.4e^-07^) and without (beta = -0.005, *p* = 1.7e^-06^) adjustment for body mass index (BMI), and in male only GWAS of SHBG (with BMI adjustment, beta = -0.007, *p* = 2.3e^-06^; without BMI adjustment, beta = -0.008, *p* = 6.6e^-07^). See Table 3 for details of the Bonferroni significant associations and Additional file 1 (Addition Table 3) for details for all nominally significant results.

**Table 3:** Bonferroni threshold significant results for IEU-GWAS-R PheWAS of rs9989237

Trait	N	Units	Per trait raising allele effect size	Per trait raising allele standard error	P
Albumin levels (inverse rank normalized transformed)	315268	Quantiles	-0.03354	0.00302	1.18E-28
Albumin levels	315268	g/L	-0.08683	0.00789	3.63E-28
*SERPINA1* RNA expression in whole blood	29950	Z-score matrices	0.123405	0.014223	4.09E-18
Expression of 39S ribosomal protein L33, mitochondrial	3301	Relative concentration	0.2515	0.0298	2.82E-17
Expression of PH and SEC7 domain-containing protein 1	3301	Relative concentration	0.1998	0.0299	2.24E-11
Expression of Synaptosomal-associated protein 25	3301	Relative concentration	-0.18	0.0299	1.78E-09
Histidine levels	114895	Z-scores	0.026138	0.005102	3.00E-07
Sex hormone-binding globulin levels adjusted for BMI	368929	Log-transformed nmol/l	-0.00535	0.00103	3.40E-07
Sex hormone-binding globulin levels (male only GWAS)	180726	Log-transformed nmol/l	-0.00772	0.001513	6.60E-07
Sex hormone-binding globulin levels	370125	Log-transformed nmol/l	-0.00515	0.001147	1.70E-06
Sex hormone-binding globulin levels adjusted for BMI (male only GWAS)	180094	Log-transformed nmol/l	-0.00712	0.001419	2.30E-06

## Discussion

We used two-sample MR with a single genetic variant to investigate the effect of maternal plasma cortisol on offspring birth weight. The results of the main analysis, the IVW analysis adjusted for between variant correlation and the SEM analysis were all directionally consistent with the observational association of higher maternal cortisol associating with lower offspring birth weight. However, all three methods of analysis used, provided imprecise estimates, which included values that are potentially of importance, as well as small or zero mean differences. For example, the 50 to 75 gram reductions in birth weight in both the main and SEM secondary analysis, respectively, together with their higher 95% confidence interval levels (both higher than 100g) are likely to be of clinical importance, whereas the lower confidence intervals (of an increase in 10 grams in the main analysis and a decrease of 9 grams in the SEM) are unlikely to be so. Therefore, the evidence of an effect of maternal cortisol on birth weight is uncertain and larger studies are required to identify whether maternal cortisol levels are a modifiable target for supporting healthy fetal growth and hence birth weight. That said, the point estimate for the association between the main genetic variant and cortisol measured in pregnancy may be considerably smaller than that seen in the original GWAS, which could mean our results are biased towards the null. In addition, with just one independent genetic variant we were unable to explore horizontal pleiotropy, using conventional two-sample MR methods and our MR PheWAS suggested that the cortisol increasing variant also related to lower mean levels of SHBG which could result in biased estimates.

A systematic review of the associations of maternal pregnancy cortisol with a range of offspring outcomes identified three studies that explored the association with offspring birth weight [26]. Two of the studies examined associations of maternal saliva cortisol and with birth weight in small numbers (70 and 55 participants). One study, which included 2810 participants, explored the association of maternal serum cortisol with birth weight [6]. Several estimates from the study suggested an inverse association with mean birth weight (ranging from a mean difference of -0.94 (95% CI, -1.75 to -0.12) to -0.07 (95% CI, -0.23 to 0.08) grams per nmol/l), which is directionally consistent with our findings. That study was not our own data, and it used different units of analyses, therefore we cannot directly compare the findings with our MR estimates. Further evidence of an inverse effect of maternal plasma cortisol on offspring birth weight came from a large (*N* = 1,858), well conducted RCT of antenatal corticosteroids in mothers at risk of preterm birth, found that randomization to antenatal corticosteroids was associated with lower offspring birth weight (mean difference -113.1 (95% CI, -187 to -41.17) grams) compared to placebo [27]. A secondary analysis of that RCT found that at least two thirds of the association could be explained by shorter gestational duration, though an effect was still detected (mean difference -33.5 (95% CI,-66.3 to -0.7) grams) [8]. Neither study reported the change in circulating corticosteroids in the mothers randomised to antenatal corticosteroid treatment compared to placebo, hence these findings cannot be compared with our MR results in the way we have previously compared MR and RCT results [28]. Lower birth weight has been associated with higher circulating cortisol in later life [29]. It is therefore possible that pregnant women with higher cortisol levels may have been smaller at birth and that an association between maternal cortisol and offspring birth weight could arise via the correlation between maternal and offspring size at birth. The birth weight effects of maternal genetic variants considered in our analyses were adjusted for the correlation with fetal genetics [12], so while this possibility remains to be investigated, it would not have influenced our results. A recent MR study on the effect of cortisol on birth weight, which has been published as part of a PhD thesis only (thus not peer-reviewed), found evidence of higher maternal cortisol leading to lower birth weight (-19 (95% CI, -34 to -7) grams per 1 log-transformed SD of cortisol). This was directionally consistent, but with a considerably weaker and more precisely estimated effect than we found. This study used an older, smaller GWAS for selecting genetic instruments than we used in this study [30, 31], which identified different genetic instruments, and used different methods to prepare the variables to adjust for between SNP correlations [32].

### Strengths and limitations

This study used a large genome-wide data set of offspring birth weight, the UK Biobank and EGG meta-analyses [12]. However, the UK Biobank and EGG meta-analyses did not adjust for gestational duration, and as maternal cortisol has been associated with gestational duration in observational studies [33], this could be an alternative mechanism by which cortisol effects birth outcomes. We used a number of novel MR techniques to measure the effect of an exposure on an outcome when only a single locus is available. Additionally, we were able to partially validate the effect of the genetic instrument on maternal pregnancy cortisol using data from the EFSOCH cohort [13].

There are two important limitations to our study which relate to the genetic instruments for cortisol. First, despite using results from the largest GWAS to date of cortisol in our main analyses we only had one genetic instrument. Nonetheless, we chose the SNP with the strongest association with cortisol (*R*^2^ = 0.2%, F-statistic = 62) for the main analysis. Furthermore, we had near identical results when combining all four genome wide associated SNPs and controlling for their correlation. However, we cannot rule out weak instrument bias resulting in an underestimate of the causative effect [16]. We were not able to undertake conventional sensitivity analyses that are more robust to potential bias due to unbalanced horizontal pleiotropy [10]. The association of the genetic instrument with SHBG, albumin and histidine in MR-Base (at a *p*-value ≤2.6e^-6^) might indicate pleiotropic effects of our genetic instrument that may have biased our results. SHBG is produced in the liver and binds to steroid hormones, as does corticosteroid-binding globulin [34], which the *SERPINA1/A6* locus encodes [11]. SHBG has been observed to be negatively associated with insulin resistance, type 2 diabetes and gestational diabetes (a cause of higher mean birth weight [35]) even after adjusting for BMI [36]. As the cortisol raising allele was associated with *lower* circulating levels of SHBG, this could result in masking pleiotropy, meaning our results are an underestimate of a true, stronger inverse effect. Circulating albumin levels are widely seen as a marker of protein sufficiency (lower levels, less sufficient), and low maternal albumin levels have been associated with lower offspring birth weight [37]. Histidine is a precursor to the inflammatory compound histamine [38], and higher maternal circulating levels of histidine have been shown to be associated with lower offspring birth weight in previous MR studies [39]. As the cortisol raising allele was associated with *lower* albumin levels and *higher* histidine levels, it could be that the suggestive evidence of a negative effect of the cortisol raising allele on birth weight is due, at least in part to pleiotropy, meaning our results could be biased. Additionally, our genetic instrument was associated with the expression of three proteins, none of which (to the best of our knowledge) has been found to be directly associated with birth weight in humans. In our PheWAS, we used a Bonferroni corrected *p*-value threshold, which is common in PheWAS exploring potential multiple causal effects of an exposure (e.g. 19,269). However, one could argue that when exploring bias this is less appropriate and we should not make this correction, or at least have a less stringent approach, as here the aim is to be as rigorous as possible in exploring potential biases [40]. Larger GWAS of circulating cortisol levels are needed to identify additional independent genetic instruments.

Our results assume that the effect of the genetic instrument on cortisol observed in the GWAS is the same as that during pregnancy. If the true effect in pregnancy is closer to what we observe in the EFSOCH pregnancy sample, then our MR analyses may be biased towards the null. Further evidence that the genetic instrument may not be valid in pregnancy comes from our PheWAS analysis, which shows the effect of rs9989237 on SHBG is stronger in men than women. However, the EFSOCH population sample is limited (*N* = 892; all in relative health) and the confidence intervals of the estimate captured the GWAS reported cortisol association. Despite this potential mitigation, the 2-fold difference between the GWAS reported cortisol association and the EFSOCH pregnancy cortisol association means there is legitimate concern that the *SERPINA1/A6* locus is a weak instrument for pregnancy cortisol, leading to bias.

## Conclusions

In conclusion, we found some evidence that higher maternal plasma cortisol may cause lower birth weight. Despite using the largest GWAS of cortisol to date, we only had one independent genetic locus and considering the potential sources of bias discussed above, more investigations are needed to make robust conclusions about the effect of maternal pregnancy cortisol on offspring birth weight.

### Supplementary Information

**Additional file 1.** 

## Data Availability

Our study uses two-sample Mendelian randomization (MR). We used both published summary results (i.e. taking results from published research papers and websites) and individual participant cohort data as follows: For the two sample MR, we used genetic variants associated with circulating plasma cortisol. We extracted the exposure associations for these genetic variants from a dataset available to download at the University of Edinburgh DataShare site. https://datashare.ed.ac.uk/handle/10283/3836#:~:text=The%20CORNET%20consortium%20extended%20its,genetic%20association%20with%20SERPINA6%2FSERPINA1 We extracted the outcome associations for these genetic instruments from genome-wide datasets of offspring birth weight adjusted for maternal genotype, available for download from the EGG Consortium. http://egg-consortium.org/birth-weight-2019.html The references to the journals that reported data sources are cited in the main paper. We used individual participant data for the second MR sample and for undertaking sensitivity analyses from the UK Biobank and EFSOCH cohorts. The data in UK Biobank is fully available, via managed systems, to any researchers. The managed system for both studies is a requirement of the study funders but access is not restricted on the basis of overlap with other applications to use the data or on the basis of peer review of the proposed science. UK Biobank. Full information on how to access these data can be found here - https://www.ukbiobank.ac.uk/using-the-resource/ EFSOCH. Requests for access to the original EFSOCH dataset should be made in writing in the first instance to the EFSOCH data team via the Exeter Clinical Research Facility crf@exeter.ac.uk.

## Abbreviations

RCT: Randomized Control Trial
MR: Mendelian Randomization
GWAS: Genome Wide Association Study
EGG: Early Growth Genetics
SNP: Single nucleotide polymorphism
