# Supplementary material for: Maternal plasma cortisol’s effect on offspring birth weight: a Mendelian Randomisation study
Source: BMC Pregnancy Childbirth. 2024 Jan 15;24:65. doi: 10.1186/s12884-024-06250-3 (PMC10789047; doi:10.1186/s12884-024-06250-3)
Supplement: Supplementary file 1 — Additional file 1. [file 12884_2024_6250_MOESM1_ESM.docx]

**Additional file 1**

Table of Contents

[**Additional Methods** 2](#_Toc153465543)

[**Additional details regarding the WLM-GWAS and SEM analysis** 2](#_Toc153465544)

[Cohort Description: UK Biobank 2](#_Toc153465545)

[Defining offspring birth weight for WLM-GWAS 2](#_Toc153465546)

[Selecting participants of European ancestry 2](#_Toc153465547)

[Genotyping 2](#_Toc153465548)

[Structural Equation Modelling and Weighted Linear Modelling theory 3](#_Toc153465549)

[Extracting birth weight data in UK Biobank for SEM analysis 3](#_Toc153465550)

[**Additional details regarding the Pregnancy Cortisol analysis** 4](#_Toc153465551)

[Cohort Description: EFSOCH 4](#_Toc153465552)

[Selecting participants of European ancestry 4](#_Toc153465553)

[Genotyping 4](#_Toc153465554)

[Measuring Pregnancy Cortisol levels in EFSOCH for sensitivity analyses 5](#_Toc153465555)

[**Calculating R^2^ and F-Statistic** 5](#_Toc153465556)

[**Additional Tables** 6](#_Toc153465557)

[**Additional Table 1: Details of all reported SNPs genome-wide associated with plasma cortisol** 6](#_Toc153465558)

[**Additional Table 2: LD Matrix correlation table (R values) for genome wide significant SNPs identified in the main GWAS of plasma cortisol[10].** 7](#_Toc153465559)

[**Additional Table 3: Results of PheWAS of rs9989237 using MR-Base** 8](#_Toc153465560)

[**References** 69](#_Toc153465561)

# **Additional Methods**

## **Additional details regarding the WLM-GWAS and SEM analysis**

In the main paper, to perform Mendelian Randomisation, we used summary statistics derived from a previously published weighted linear model (WLM) applied to GWAS results of maternal and fetal effects on offspring birth weight using data from the UK Biobank and the Early Growth Genetics Consortium [1]. The WLM is an approximation of a computationally intensive structural equation model (SEM) [1, 2], so we also fitted the SEM to the UK Biobank sample to check that the results were similar.

### Cohort Description: UK Biobank

Between 2006 and 2010, patients were recruited from the NHS patient registers and contacted if they lived in close proximity to one of 22 assessment centres in England, Scotland and Wales. Detailed medical data was collected on 502,655 participants, aged between 40 and 69 at recruitment [3]. A total of 190,406 women in the UK Biobank cohort who had reported their first child’s birth weight were included in the UK Biobank and EGG meta-analysis WLM-GWAS, which was used for the main analysis on the effect of maternal cortisol on birth weight of this paper [1]. All participants provided written informed consent, including for their collected data to be used by international scientists. UK Biobank has approval from the North West Multi-centre Research Ethics Committee (MREC), which covers the UK. UK Biobank’s research ethics committee and Human Tissue Authority research tissue bank approvals mean that researchers wishing to use the resource do not need separate ethics approval.

The UK Biobank GWAS summary statistics had been meta-analysed with GWAS summary statistics from EGG consortium studies and a WLM then applied to estimate maternal SNP effect estimates adjusted for fetal effects [1].

### Defining offspring birth weight for WLM-GWAS

For the EGG consortium GWAS we used for our primary analyses, 90% of the participants came from UK Biobank. In UK Biobank, multiple births preterm births were excluded (preterm births defined as those < 2.2 kg due to a lack of gestational age information), and 90% of the birth weight values were self-reported [1].

### Selecting participants of European ancestry

In UK Biobank, we defined a subset of “European” ancestry participants for inclusion in for all of our analyses that used UK Biobank. To do this, we generated ancestry principal components (PCs) in the 1000 genomes samples. The UK Biobank samples were then projected into this PC space using the SNP loadings obtained from the principal components analysis using the 1000 genomes samples. The UK Biobank participants’ ancestry was classified using K-means clustering centred on the 3 main 1000 genomes populations (European, African, South Asian). Those clustering with the European cluster were classified as having European ancestry. The UK Biobank participants were asked to report their ethnic background. Only those reporting as either “British”, “Irish”, “White” or “Any other white background” were included in the clustering analysis.

### Genotyping

We analysed data from the May 2017 release of imputed genetic data (which has been extensively described elsewhere)[4], which was then pooled with the results from the EGG GWAS[1]. Given the reported technical error with non-HRC imputed variants[5], we focused exclusively on the set of ~40M imputed variants from the HRC reference panel. As we decided to use Structural Equation Modelling (SEM) as a sensitivity analyses separate from the overall UK Biobank + EGG analyses (see Data Analyses in main paper), we also analysed the UK Biobank participants separately.

To account for population structure and relatedness in UK Biobank, a linear mixed model implemented in BOLT-LMM v2.3[6] was used to perform genome-wide association (GWA) analysis of BW in the UKB sample. Only autosomal single nucleotide polymorphisms (SNPs) which were common (MAF>1%), in Hardy Weinberg equilibrium (p value > 1x10-6), passed QC in all 106 batches and were present on both genotyping arrays were included in the genetic relationship matrix (GRM). For the GWA analyses of birth weight of the first child (i.e. using the maternal genotype), the genotyping array and genotyping release (interim vs. full) were included as covariates in the regression model. For the GWAS of participants own birth weight, genotyping array, age at baseline and sex were adjusted for in all models.

### Structural Equation Modelling and Weighted Linear Modelling theory

SEM can be used to estimate the maternal specific genetic effect on offspring birth weight in the absence of data from genotyped mother-child pairs [2]. The model uses the participant’s own genotype, own birth weight and offspring’s birth weight as observed data. For each individual, these observed variables are modelled as functions of two latent (unobserved) variables, the individual’s mother’s genotype and the individual’s offspring’s genotype, which are both correlated 0.5 with the participants own genotype. A full description of SEM can be found in Warrington et al 2018 [2]. In brief, the model uses the variances and co-variances between the observed variables (own birth weight, offspring birth weight and own genotype), to estimate the parameters of interest including that maternal and fetal effects on birth weight. The model is flexible in that it can incorporate a subsect of participants with only their own birth weight and own genotype as well as a subsect of participant’s with only their own genotype (including males) and offspring birth weight. However, fitting the model using full information maximum likelihood is computationally intensive, making it difficult to use a genome wide level. Consequently, the authors developed a linear approximation of the full SEM that yielded similar effect estimates and standard errors but was computationally more efficient. This linear approximation, referred to as a WLM, combined the unadjusted maternal and fetal effect estimates at a single locus, using the following formula

$$\hat{\beta}_{m_{adj}}= \frac{4}{3}\hat{\beta}_{m_{unadj}}-\frac{2}{3}\hat{\beta}_{f_{unadj}}$$

where $\hat{\beta}_{m_{adj}}$ is the maternal specific genetic effect on the outcome (here, offspring birth weight),$\hat{\beta}_{m_{unadj}}$ is the unadjusted maternal genetic effect from a GWAS of maternal genotype on offspring birth weight and $\hat{\beta}_{f_{unadj}}$is the unadjusted fetal genetic effect from a GWAS of own genotype on own birth weight [1, 2].

The WLM is also used to estimate the standard error of the SNP-maternal specific effect, using the following formula

$$SE\left( \hat{\beta}_{m_{adj}} \right)= \sqrt{\left( \frac{16}{9}var(\hat{\beta}_{m_{unadj}})+\frac{4}{9}var(\hat{\beta}_{f_{unadj}}) \right)}$$

The estimated maternal specific genetic effect from the WLM,$\hat{\beta}_{m_{adj}}$, has been shown to be asymptotically equivalent to the estimated effect from a conditional linear model in mother-child pairs, where offspring birth weight is regressed on the maternal and offspring genotype [1].

### Extracting birth weight data in UK Biobank for SEM analysis

Female participants (N=273,495) were also asked to report the birth weight of their first child. Female participants that reported having a multiple first birth were excluded from our analyses (N=1,364). A total of 216,839 women with a singleton pregnancy for their first child, reported the birth weight of their first child. Values were reported to the nearest whole pounds, and were converted to kilograms, by multiplying by 0.454, for our analyses. Where women reported the birth weight of the first child at multiple time points (N=11,353) we used the mean of all measures after excluding any women with a difference of >1kg between any two measures (N=31). We further excluded from the whole sample any women who reported the birth weight of their first child <2.2kg or >4.6kg (N=6,333). This was done to reduce bias from reporting errors and, in relation to those <2.2kg to exclude extreme preterm births given we do not have information on gestational age. Birth weight of first child was regressed against the women’s reported age at first birth and her UK Biobank assessment centre location to reduce heterogeneity in reporting bias by these characteristics. Residuals from that regression model were then used in all analyses with values converted to standard deviation units for analysis. For the SEM analysis, we restricted the sample further, including only those from a determined list of unrelated white European participants, leaving 162,827 participants reporting the birth weight of their first child in total.

In order to perform SEM in UK Biobank, we needed data on participants own birth weight. A total of 280,315 participants reported their own birth weight in kilograms at either the baseline visit or at least one of the follow-up visits. Participants reporting being part of a multiple birth were excluded from our analyses (N=10,057). For participants reporting birth weight at more than one visit (N=11,629), the average across the reported birth weights were used, and if the largest difference between any 2 time points was >1kg, they were excluded (N=80). Data on gestational duration were not available. However, in order to exclude likely pre-term births, participants with birth weight values <2.5kg were excluded. We also excluded those with a birth weight >4.5kg as these are likely to be reporting errors or extreme outliers (total number excluded because of <2.5kg or >4.5kg birth weight =37,691). Participants’ own birth weight was regressed against year of birth and assessment centre location. Residuals from that regression model were then used in all analyses with values converted to standard deviation units for analysis. For the SEM analysis, we restricted the sample further, including only those from a determined list of unrelated white European participants, leaving 180,810 participants reporting their own birth weight in total.

## **Additional details regarding the Pregnancy Cortisol analysis**

### Cohort Description: EFSOCH

Between 2000 and 2004, pregnant women from a postcode defined region of Exeter, UK and their partners were recruited via the Exeter Maternity Unit database. A total of 1,017 families (98% white European) were recruited [7], from which a total of 993 live births were included in the primary analyses of this paper. All mothers and fathers gave informed consent and ethical approval was obtained from the North and East Devon Local Research Committee.

### Selecting participants of European ancestry

EFSOCH only included participants of white British origin [7]. Nonetheless, principal component analysis was performed to assess ancestry of the sample using flashPCA [8]. Outliers were defined as >4.56 SD from the cluster mean (defined using 1000 Genomes European data as the reference) and excluded (n=21 individuals [0.76%]).

### Genotyping

For EFSOCH, the SNP used in this study was taken from genome-wide imputed data that had been completed for both the mothers and their offspring (fetal genotype). In EFSOCH maternal and fetal data were obtained from the Illumina Infinium HumanCoreExome-24. Genotype data were imputed against Haplotype Reference Consortium HRC v1.1 reference panel after quality control (MAF >1%, HWE>1×10^-6^, sex mismatch, kinship errors and 4.56 SD from the cluster mean of any sub-populations cluster).

### Measuring Pregnancy Cortisol levels in EFSOCH for sensitivity analyses

We received in total information on 1,011 mother’s cortisol levels at 28 weeks gestation within 60 minutes of 9am. The serum cortisol levels were measured at the King Edward Memorial laboratory in Pune, India using an ALPCO kit (assay measuring range 0.5-60 μg/dl). The samples were taken as blood from the mother, spun to separate out the plasma and stored at -10 °C until further use. The samples were then tested for cortisol using an ELISA assay which involved Rabbit cortisol antibodies to bind to the cortisol to the microplate, cortisol-horseradish peroxidase conjugate to link cortisol levels to a detectable reaction and TMB with hydrogen peroxide for that reaction. The chemical reaction was measured via optical density and the levels of cortisol were estimated from a calibration curve. The inter-assay coefficient of variation was 7.7% and 9.2% at 43 μg/dl and 14 μg/dl respectively. All analyses was performed using a single reagent lot. For each microplate, a low and high internal quality control sample, as well as a randomly selected patient sample, were run in duplicate for this analyses (16, 43 and 14 μg/dl respectively).

Of the 1,011 mothers, 72 were randomly assigned to have their cortisol level repeat measured. If the difference between the new value and the repeat value was greater than 18%, that individual was excluded from further analysis. In total 10 mothers were dropped for this reason.

Before measuring the association between the GWAS identified SNP and pregnancy cortisol in EFSOCH, we excluded 26 participants as part of standard EFSOCH protocol. The reasons for excluding participants included non-European ancestry, participants leaving the area, delivery of the baby away from Exeter, participants leaving the study, stillbirth, terminal illness, twin births, chromosome abnormalities, cerebral palsy, premature delivery, presence of Glucokinase mutations and gestational diabetes.

## **Calculating R^2^ and F-Statistic**

In regression analyses, the R^2^ value is the amount of variance explained by a given model, whilst the F-statistic shows how much the model differs from the null hypothesis that there is no mean difference between two subgroups. As Crawford et al 2021 [9] did not provide F-statistics for their results, and only provided the variance explained for the lead SNP (rs9989237), we decided to calculate these values for the SNP-cortisol association ourselves. We calculated the R^2^ value using the following equation

$$R^{2}=2\times\beta^{2}\times MAF\times(1-MAF)$$

where $\beta$ is the SNP effect in SD units and MAF is the minor allele frequency of the SNP.

Using the R^2^ value we calculated ourselves, we calculated the F-statistic with the following equation

$$F=\frac{N-K-1}{K}\frac{R^{2}}{1-R^{2}}$$

where N is the number of GWAS participants and K is the number of SNPs (in our case one each time).

# **Additional Tables**

## **Additional Table 1: Details of all reported SNPs genome-wide associated with plasma cortisol**

| SNP | Nearest/Nearby gene | Trait raising allele | Trait lowering allele | Trait raising allele frequency | Beta for trait | Standard Error for trait | P-value for trait | Units for Beta | Source |
| --- | --- | --- | --- | --- | --- | --- | --- | --- | --- |
| rs11620763 | *SERPINA6/SERPINA1* | G | A | 0.81 | 0.09 | 0.01 | 1.97E-10 | Z-scores of log transformed cortisol levels^1^ | Crawford et al 2021 [9] |
| rs2736898 | *SERPINA6/SERPINA1* | T | C | 0.49 | 0.06 | 0.01 | 7.03E-14 | Z-scores of log transformed cortisol levels^1^ | Crawford et al 2021 [9] |
| rs7146221 | *SERPINA6/SERPINA1* | G | A | 0.55 | 0.05 | 0.01 | 6.28E-10 | Z-scores of log transformed cortisol levels^1^ | Crawford et al 2021 [9] |
| rs9989237 | *SERPINA6/SERPINA1* | T | C | 0.21 | 0.09 | 0.01 | 2.16E-19 | Z-scores of log transformed cortisol levels^1^ | Crawford et al 2021 [9] |

1. The mean cortisol levels for the contributing studies in Crawford et al 2021 [9] were presented in nmol/l in the supplementary material (with the exception of three which measured relative concentration). However, it is unclear in the paper whether this is actually the units measured in the original analysis.

## **Additional Table 2: LD Matrix correlation table (R values) for genome wide significant SNPs identified in the main GWAS of plasma cortisol[10].**

|  | rs9989237 | rs2736898 | rs11620763 | rs7146221 |
| --- | --- | --- | --- | --- |
| rs9989237 | 1 | 0.493006 | -0.182103 | -0.189644 |
| rs2736898 | 0.493006 | 1 | -0.217721 | -0.371343 |
| rs11620763 | -0.182103 | -0.217721 | 1 | 0.524575 |
| rs7146221 | -0.189644 | -0.371343 | 0.524575 | 1 |

## **Additional Table 3: Results of PheWAS of rs9989237 using MR-Base**

| Trait | chromosome | position | rsid | effect allele | other allele | effect allele frequency | n | per trait raising allele effect size | per trait raising allele standard error | p | gwas id |
| --- | --- | --- | --- | --- | --- | --- | --- | --- | --- | --- | --- |
| Albumin | 14 | 94795202 | rs9989237 | T | C | 0.20992 | 315268 | -0.03354 | 0.00302 | 1.18E-28 | ukb-d-30600_irnt |
| Albumin | 14 | 94795202 | rs9989237 | T | C | 0.20992 | 315268 | -0.08683 | 0.00789 | 3.63E-28 | ukb-d-30600_raw |
| ENSG00000197249 | 14 | 94795202 | rs9989237 | T | C | 0.22464 | 29950 | 0.123405 | 0.014223 | 4.09E-18 | eqtl-a-ENSG00000197249 |
| 39S ribosomal protein L33, mitochondrial | 14 | 94795202 | rs9989237 | T | C | 0.21768 | 3301 | 0.2515 | 0.0298 | 2.82E-17 | prot-a-1942 |
| PH and SEC7 domain-containing protein 1 | 14 | 94795202 | rs9989237 | T | C | 0.21768 | 3301 | 0.1998 | 0.0299 | 2.24E-11 | prot-a-2398 |
| Synaptosomal-associated protein 25 | 14 | 94795202 | rs9989237 | T | C | 0.21768 | 3301 | -0.18 | 0.0299 | 1.78E-09 | prot-a-2786 |
| Histidine | 14 | 94795202 | rs9989237 | T | C | 0.209424 | NA | 0.026138 | 0.005102 | 3.00E-07 | met-d-His |
| Sex hormone-binding globulin levels adjusted for BMI | 14 | 94795202 | rs9989237 | T | C | 0.210079 | 368929 | -0.00535 | 0.00103 | 3.40E-07 | ebi-a-GCST90012110 |
| Sex hormone-binding globulin levels | 14 | 94795202 | rs9989237 | T | C | 0.210149 | 180726 | -0.00772 | 0.001513 | 6.60E-07 | ebi-a-GCST90012109 |
| Sex hormone-binding globulin levels | 14 | 94795202 | rs9989237 | T | C | 0.21008 | 370125 | -0.00515 | 0.001147 | 1.70E-06 | ebi-a-GCST90012111 |
| Sex hormone-binding globulin levels adjusted for BMI | 14 | 94795202 | rs9989237 | T | C | 0.21013 | 180094 | -0.00712 | 0.001419 | 2.30E-06 | ebi-a-GCST90012108 |
| Bioavailable testosterone levels | 14 | 94795202 | rs9989237 | T | C | 0.210077 | 382988 | 0.011675 | 0.002451 | 3.00E-06 | ebi-a-GCST90012104 |
| NHP2-like protein 1 | 14 | 94795202 | rs9989237 | T | C | 0.21768 | 3301 | -0.1394 | 0.03 | 3.31E-06 | prot-a-2048 |
| Calcium | 14 | 94795202 | rs9989237 | T | C | 0.20991 | 315153 | -0.01408 | 0.003043 | 3.74E-06 | ukb-d-30680_irnt |
| SHBG | 14 | 94795202 | rs9989237 | T | C | 0.20983 | 312215 | -0.01249 | 0.002782 | 7.11E-06 | ukb-d-30830_irnt |
| Calcium | 14 | 94795202 | rs9989237 | T | C | 0.20991 | 315153 | -0.00127 | 0.000286 | 8.29E-06 | ukb-d-30680_raw |
| cAMP-regulated phosphoprotein 21 | 14 | 94795202 | rs9989237 | T | C | 0.21768 | 3301 | 0.1333 | 0.03 | 8.71E-06 | prot-a-168 |
| Albumin | 14 | 94795202 | rs9989237 | T | C | 0.209418 | NA | -0.02231 | 0.005088 | 1.20E-05 | met-d-Albumin |
| Nuclear receptor subfamily 1 group D member 1 | 14 | 94795202 | rs9989237 | T | C | 0.21768 | 3301 | 0.1283 | 0.03 | 1.91E-05 | prot-a-2085 |
| Sex hormone binding globulin (SHBG) | 14 | 94795202 | rs9989237 | T | C | 0.210515 | NA | -0.01661 | 0.003895 | 2.00E-05 | ieu-b-4871 |
| IDP T1 SIENAX white unnormalised volume | 14 | 94795202 | rs9989237 | T | C | 0.21004 | 7916 | 0.0373 | 0.0089 | 2.63E-05 | ubm-a-8 |
| IDP T1 SIENAX white normalised volume | 14 | 94795202 | rs9989237 | T | C | 0.21004 | 7916 | 0.0721 | 0.0172 | 2.69E-05 | ubm-a-7 |
| T-cell receptor-associated transmembrane adapter 1 | 14 | 94795202 | rs9989237 | T | C | 0.21768 | 3301 | 0.1255 | 0.03 | 2.88E-05 | prot-a-3097 |
| SHBG | 14 | 94795202 | rs9989237 | T | C | 0.20983 | 312215 | -0.32412 | 0.077914 | 3.18E-05 | ukb-d-30830_raw |
| Glia-derived nexin | 14 | 94795202 | rs9989237 | T | C | 0.21768 | 3301 | 0.1243 | 0.03 | 3.39E-05 | prot-a-2697 |
| IDP T1 SIENAX brain-normalised volume | 14 | 94795202 | rs9989237 | T | C | 0.21004 | 7916 | 0.0601 | 0.0147 | 4.47E-05 | ubm-a-9 |
| IDP T1 SIENAX brain-unnormalised volume | 14 | 94795202 | rs9989237 | T | C | 0.21004 | 7916 | 0.0309 | 0.0076 | 4.57E-05 | ubm-a-10 |
| Cholesteryl esters to total lipids ratio in medium LDL | 14 | 94795202 | rs9989237 | T | C | 0.20942 | NA | -0.02043 | 0.005066 | 4.80E-05 | met-d-M_LDL_CE_pct |
| R-spondin-3 | 14 | 94795202 | rs9989237 | T | C | 0.21768 | 3301 | 0.1209 | 0.03 | 5.50E-05 | prot-a-2605 |
| Proliferating cell nuclear antigen | 14 | 94795202 | rs9989237 | T | C | 0.21768 | 3301 | 0.121 | 0.03 | 5.50E-05 | prot-a-2208 |
| Cholesteryl esters to total lipids ratio in small LDL | 14 | 94795202 | rs9989237 | T | C | 0.20942 | NA | -0.02054 | 0.00508 | 5.90E-05 | met-d-S_LDL_CE_pct |
| Liver enzyme levels (alanine transaminase) | 14 | 94795202 | rs9989237 | T | C | 0.20997 | 437267 | -0.00192 | 0.000477 | 6.50E-05 | ebi-a-GCST90013405 |
| Phospholipids to total lipids ratio in small LDL | 14 | 94795202 | rs9989237 | T | C | 0.20942 | NA | 0.020345 | 0.005079 | 7.00E-05 | met-d-S_LDL_PL_pct |
| Choriogonadotropin subunit beta variant 2 | 14 | 94795202 | rs9989237 | T | C | 0.21768 | 3301 | 0.119 | 0.03 | 7.24E-05 | prot-a-531 |
| Serpin A12 levels | 14 | 94795202 | rs9989237 | T | C | 0.236 | 1301 | -0.18638 | 0.046487 | 7.28E-05 | ebi-a-GCST90010170 |
| High affinity cAMP-specific 3',5'-cyclic phosphodiesterase 7A | 14 | 94795202 | rs9989237 | T | C | 0.21768 | 3301 | 0.1178 | 0.03 | 8.51E-05 | prot-a-2223 |
| AMSH-like protease | 14 | 94795202 | rs9989237 | T | C | 0.21768 | 3301 | 0.1176 | 0.03 | 8.91E-05 | prot-a-2864 |
| Free cholesterol to total lipids ratio in large VLDL | 14 | 94795202 | rs9989237 | T | C | 0.209427 | NA | -0.01869 | 0.00504 | 9.30E-05 | met-d-L_VLDL_FC_pct |
| Activated Protein C | 14 | 94795202 | rs9989237 | T | C | 0.21768 | 3301 | 0.1156 | 0.03 | 0.000117 | prot-a-2381 |
| Non-cancer illness year/age first occurred | 14 | 94795202 | rs9989237 | T | C | 0.7941 | NA | -0.1019 | 0.02657 | 0.000127 | ukb-e-87_AFR |
| Respiratory disorders in diseases classified elsewhere | 14 | 94795202 | rs9989237 | T | C | 0.2083 | NA | -0.5295 | 0.1383 | 0.000129 | finn-b-RESP_ILL_DIS_CLASS_ELSEWHERE |
| volume rhCerebralWhiteMatterVol | 14 | 94795202 | rs9989237 | T | C | 0.21004 | 7916 | 0.0375 | 0.0099 | 0.000145 | ubm-a-2701 |
| Hepatocyte nuclear factor 1-alpha | 14 | 94795202 | rs9989237 | T | C | 0.21768 | 3301 | 0.1138 | 0.03 | 0.000148 | prot-a-1359 |
| Stromal cell-derived factor 1 | 14 | 94795202 | rs9989237 | T | C | 0.21768 | 3301 | 0.1135 | 0.03 | 0.000155 | prot-a-741 |
| Amyloid-like protein 2 | 14 | 94795202 | rs9989237 | T | C | 0.21768 | 3301 | 0.1131 | 0.03 | 0.000166 | prot-a-122 |
| volume CerebralWhiteMatterVol | 14 | 94795202 | rs9989237 | T | C | 0.21004 | 7916 | 0.0371 | 0.0099 | 0.00017 | ubm-a-2702 |
| Age hay fever, rhinitis or eczema diagnosed | 14 | 94795202 | rs9989237 | T | C | 0.7826 | NA | -0.134 | 0.03564 | 0.00017 | ukb-e-3761_AFR |
| Platelet-derived growth factor D | 14 | 94795202 | rs9989237 | T | C | 0.21768 | 3301 | 0.1124 | 0.03 | 0.000182 | prot-a-2228 |
| Gamma glutamyltransferase | 14 | 94795202 | rs9989237 | T | C | 0.20966 | 344104 | -0.01019 | 0.002725 | 0.000184 | ukb-d-30730_irnt |
| Synaptotagmin-11 | 14 | 94795202 | rs9989237 | T | C | 0.21768 | 3301 | 0.1117 | 0.03 | 0.0002 | prot-a-2911 |
| volume lhCerebralWhiteMatterVol | 14 | 94795202 | rs9989237 | T | C | 0.21004 | 7916 | 0.037 | 0.0099 | 0.0002 | ubm-a-2700 |
| Platelet count | 14 | 94795202 | rs9989237 | T | C | 0.2104 | 166066 | -0.01655 | 0.004465 | 0.000211 | ebi-a-GCST004603 |
| Phospholipids to total lipids ratio in medium LDL | 14 | 94795202 | rs9989237 | T | C | 0.20942 | NA | 0.019662 | 0.005084 | 0.00022 | met-d-M_LDL_PL_pct |
| Urea transporter 2 | 14 | 94795202 | rs9989237 | T | C | 0.21768 | 3301 | -0.1108 | 0.03 | 0.000224 | prot-a-2747 |
| ENSG00000119698 | 14 | 94795202 | rs9989237 | T | C | 0.22464 | 29375 | -0.05236 | 0.014254 | 0.000239 | eqtl-a-ENSG00000119698 |
| Alanine aminotransferase | 14 | 94795202 | rs9989237 | T | C | 0.20966 | 344136 | -0.01001 | 0.002742 | 0.00026 | ukb-d-30620_irnt |
| Probable carboxypeptidase X1 | 14 | 94795202 | rs9989237 | T | C | 0.21768 | 3301 | 0.1096 | 0.03 | 0.000263 | prot-a-648 |
| Glucocorticoid modulatory element-binding protein 2 | 14 | 94795202 | rs9989237 | T | C | 0.21768 | 3301 | 0.1084 | 0.03 | 0.000302 | prot-a-1224 |
| Bile salt-activated lipase | 14 | 94795202 | rs9989237 | T | C | 0.21768 | 3301 | 0.1083 | 0.03 | 0.000309 | prot-a-507 |
| Total protein | 14 | 94795202 | rs9989237 | T | C | 0.20994 | 314921 | -0.04479 | 0.012434 | 0.000316 | ukb-d-30860_raw |
| Average diameter for VLDL particles | 14 | 94795202 | rs9989237 | T | C | 0.20942 | NA | -0.01593 | 0.004747 | 0.00033 | met-d-VLDL_size |
| Gastrin-releasing peptide | 14 | 94795202 | rs9989237 | T | C | 0.21768 | 3301 | 0.1077 | 0.03 | 0.000331 | prot-a-1278 |
| Eukaryotic translation initiation factor 4E-binding protein 2 | 14 | 94795202 | rs9989237 | T | C | 0.21768 | 3301 | 0.1075 | 0.03 | 0.000339 | prot-a-924 |
| Cholesterol in large VLDL | 14 | 94795202 | rs9989237 | T | C | 0.20942 | NA | -0.01709 | 0.004979 | 0.00034 | met-d-L_VLDL_C |
| Total testosterone levels | 14 | 94795202 | rs9989237 | T | C | 0.20952 | 230454 | 0.012025 | 0.003444 | 0.00034 | ebi-a-GCST90012112 |
| Platelet count | 14 | 94795202 | rs9989237 | T | C | 0.209416 | 350474 | -0.01016 | 0.002836 | 0.000342 | ukb-d-30080_irnt |
| Total protein | 14 | 94795202 | rs9989237 | T | C | 0.20994 | 314921 | -0.01103 | 0.003083 | 0.000347 | ukb-d-30860_irnt |
| Free cholesterol in large VLDL | 14 | 94795202 | rs9989237 | T | C | 0.20942 | NA | -0.01678 | 0.004925 | 0.00035 | met-d-L_VLDL_FC |
| Platelet-derived growth factor subunit A | 14 | 94795202 | rs9989237 | T | C | 0.21768 | 3301 | 0.1072 | 0.03 | 0.000355 | prot-a-2225 |
| Cholesteryl esters in large VLDL | 14 | 94795202 | rs9989237 | T | C | 0.20942 | NA | -0.01712 | 0.005029 | 0.00038 | met-d-L_VLDL_CE |
| HLA class II histocompatibility antigen, DP beta 1 chain | 14 | 94795202 | rs9989237 | T | C | 0.21768 | 3301 | -0.1067 | 0.03 | 0.00038 | prot-a-1346 |
| volume SupraTentorialVol | 14 | 94795202 | rs9989237 | T | C | 0.21004 | 7916 | 0.0272 | 0.0076 | 0.00038 | ubm-a-2705 |
| Syntaxin-2 | 14 | 94795202 | rs9989237 | T | C | 0.21768 | 3301 | -0.1066 | 0.03 | 0.000389 | prot-a-2887 |
| Disorders of plasma-protein metabolism, not elsewhere classified | 14 | 94795202 | rs9989237 | T | C | 0.2083 | NA | -0.7115 | 0.2011 | 0.000402 | finn-b-E4_PLASMAPROT |
| Stabilin-1 | 14 | 94795202 | rs9989237 | T | C | 0.21768 | 3301 | 0.1062 | 0.03 | 0.000407 | prot-a-2862 |
| Histone acetyltransferase type B catalytic subunit | 14 | 94795202 | rs9989237 | T | C | 0.21768 | 3301 | 0.1059 | 0.03 | 0.000417 | prot-a-1309 |
| DnaJ homolog subfamily B member 11 | 14 | 94795202 | rs9989237 | T | C | 0.21768 | 3301 | 0.1058 | 0.03 | 0.000427 | prot-a-836 |
| Concentration of large VLDL particles | 14 | 94795202 | rs9989237 | T | C | 0.20942 | NA | -0.01643 | 0.004916 | 0.00043 | met-d-L_VLDL_P |
| Average diameter for LDL particles | 14 | 94795202 | rs9989237 | T | C | 0.20942 | NA | 0.0176 | 0.004972 | 0.00044 | met-d-LDL_size |
| Bioavailable testosterone levels | 14 | 94795202 | rs9989237 | T | C | 0.210009 | 188507 | 0.010961 | 0.003109 | 0.00046 | ebi-a-GCST90012102 |
| Non-cancer illness code, self-reported: eczema/dermatitis | 14 | 94795202 | rs9989237 | T | C | 0.209974 | 462933 | 0.00141 | 0.000402 | 0.00046 | ukb-b-20141 |
| DKTatlas lh WhiteSurfArea area | 14 | 94795202 | rs9989237 | T | C | 0.21004 | 7916 | 0.0317 | 0.0091 | 0.000501 | ubm-a-2746 |
| DKTatlas rh WhiteSurfArea area | 14 | 94795202 | rs9989237 | T | C | 0.21004 | 7916 | 0.0315 | 0.009 | 0.000501 | ubm-a-2852 |
| Phospholipids to total lipids ratio in very large HDL | 14 | 94795202 | rs9989237 | T | C | 0.209409 | NA | 0.014436 | 0.004676 | 0.00052 | met-d-XL_HDL_PL_pct |
| Zona pellucida-like domain-containing protein 1 | 14 | 94795202 | rs9989237 | T | C | 0.21768 | 3301 | -0.1041 | 0.03 | 0.000525 | prot-a-3282 |
| Profilin-2 | 14 | 94795202 | rs9989237 | T | C | 0.21768 | 3301 | -0.1039 | 0.03 | 0.000537 | prot-a-2253 |
| volume BrainSegVolNotVentSurf | 14 | 94795202 | rs9989237 | T | C | 0.21004 | 7916 | 0.027 | 0.0078 | 0.00055 | ubm-a-2696 |
| Total Testosterone | 14 | 94795202 | rs9989237 | T | C | 0.210288 | NA | 0.013313 | 0.003854 | 0.00055 | ieu-b-4864 |
| Diagnoses - main ICD10: H25.1 Senile nuclear cataract | 14 | 94795202 | rs9989237 | T | C | 0.209969 | 463010 | -0.00057 | 0.000166 | 0.00055 | ukb-b-3095 |
| ENSG00000165949 | 14 | 94795202 | rs9989237 | T | C | 0.22464 | 29950 | -0.04914 | 0.014255 | 0.000566 | eqtl-a-ENSG00000165949 |
| Lymphotactin | 14 | 94795202 | rs9989237 | T | C | 0.21768 | 3301 | 0.1034 | 0.03 | 0.000575 | prot-a-3237 |
| Angiopoietin-1 | 14 | 94795202 | rs9989237 | T | C | 0.21768 | 3301 | 0.1031 | 0.03 | 0.000589 | prot-a-92 |
| Ratio of monounsaturated fatty acids to total fatty acids | 14 | 94795202 | rs9989237 | T | C | 0.209439 | NA | -0.01608 | 0.004881 | 0.00063 | met-d-MUFA_pct |
| Mean reticulocyte volume | 14 | 94795202 | rs9989237 | T | C | 0.209449 | 344728 | 0.010039 | 0.002937 | 0.000632 | ukb-d-30260_irnt |
| Used an inhaler for chest within last hour | 14 | 94795202 | rs9989237 | T | C | 0.210118 | 424873 | -0.00077 | 0.000226 | 0.00064 | ukb-b-11188 |
| 72 kDa inositol polyphosphate 5-phosphatase | 14 | 94795202 | rs9989237 | T | C | 0.21768 | 3301 | 0.1025 | 0.03 | 0.000646 | prot-a-1557 |
| volume BrainSegVol | 14 | 94795202 | rs9989237 | T | C | 0.21004 | 7916 | 0.0256 | 0.0075 | 0.000646 | ubm-a-2694 |
| Neuron-specific protein family member 2 | 14 | 94795202 | rs9989237 | T | C | 0.21768 | 3301 | 0.1023 | 0.03 | 0.000661 | prot-a-2111 |
| Natural cytotoxicity triggering receptor 2 | 14 | 94795202 | rs9989237 | T | C | 0.21768 | 3301 | 0.1022 | 0.03 | 0.000661 | prot-a-2015 |
| Concentration of medium LDL particles | 14 | 94795202 | rs9989237 | T | C | 0.20942 | NA | -0.01663 | 0.005103 | 0.00067 | met-d-M_LDL_P |
| Intake of artificial sweetener added to tea | 14 | 94795202 | rs9989237 | T | C | 0.210565 | 64949 | 0.01321 | 0.003885 | 0.00067 | ukb-b-5867 |
| Nucleosome assembly protein 1-like 2 | 14 | 94795202 | rs9989237 | T | C | 0.21768 | 3301 | -0.102 | 0.03 | 0.000676 | prot-a-1999 |
| volume MaskVol | 14 | 94795202 | rs9989237 | T | C | 0.21004 | 7916 | 0.0239 | 0.007 | 0.000676 | ubm-a-2708 |
| Ratio of polyunsaturated fatty acids to monounsaturated fatty acids | 14 | 94795202 | rs9989237 | T | C | 0.209439 | NA | 0.016182 | 0.004882 | 0.00068 | met-d-PUFA_by_MUFA |
| Low density lipoprotein cholesterol levels | 14 | 94795202 | rs9989237 | T | C | 0.209983 | 431167 | -0.00799 | 0.002356 | 0.00069 | ebi-a-GCST90002412 |
| Leucine-rich repeat-containing protein 4B | 14 | 94795202 | rs9989237 | T | C | 0.21768 | 3301 | 0.1019 | 0.03 | 0.000692 | prot-a-1788 |
| Concentration of very large VLDL particles | 14 | 94795202 | rs9989237 | T | C | 0.20942 | NA | -0.01586 | 0.004894 | 0.00072 | met-d-XL_VLDL_P |
| Total lipids in small VLDL | 14 | 94795202 | rs9989237 | T | C | 0.20942 | NA | -0.01616 | 0.00504 | 0.00074 | met-d-S_VLDL_L |
| Desmocollin-3 | 14 | 94795202 | rs9989237 | T | C | 0.21768 | 3301 | 0.101 | 0.03 | 0.000759 | prot-a-867 |
| Tolloid-like protein 1 | 14 | 94795202 | rs9989237 | T | C | 0.21768 | 3301 | -0.1009 | 0.03 | 0.000776 | prot-a-2986 |
| Core-binding factor subunit beta | 14 | 94795202 | rs9989237 | T | C | 0.21768 | 3301 | -0.1009 | 0.03 | 0.000776 | prot-a-369 |
| Total lipids in large VLDL | 14 | 94795202 | rs9989237 | T | C | 0.20942 | NA | -0.01568 | 0.00492 | 0.00078 | met-d-L_VLDL_L |
| DKTatlas rh caudalmiddlefrontal area | 14 | 94795202 | rs9989237 | T | C | 0.21004 | 7916 | 0.0523 | 0.0156 | 0.000794 | ubm-a-2822 |
| volume SupraTentorialVolNotVent | 14 | 94795202 | rs9989237 | T | C | 0.21004 | 7916 | 0.027 | 0.0081 | 0.000851 | ubm-a-2706 |
| Diabetic neuropathy | 14 | 94795202 | rs9989237 | T | C | 0.2076 | NA | -0.1604 | 0.0481 | 0.000857 | finn-b-DM_NEUROPATHY |
| Triglycerides to total lipids ratio in very large HDL | 14 | 94795202 | rs9989237 | T | C | 0.209409 | NA | -0.01535 | 0.004799 | 0.00086 | met-d-XL_HDL_TG_pct |
| 6mm cylindrical power angle (right) | 14 | 94795202 | rs9989237 | T | C | 0.6112 | NA | -0.077 | 0.02313 | 0.00087 | ukb-e-5114_CSA |
| Concentration of small VLDL particles | 14 | 94795202 | rs9989237 | T | C | 0.20942 | NA | -0.01592 | 0.005025 | 0.00087 | met-d-S_VLDL_P |
| Illnesses of mother | 14 | 94795202 | rs9989237 | T | C | 0.6107 | NA | 0.3147 | 0.09465 | 0.000883 | ukb-e-20110_p11_CSA |
| UDP-glucuronosyltransferase 2A1 | 14 | 94795202 | rs9989237 | T | C | 0.21768 | 3301 | 0.0998 | 0.03 | 0.000891 | prot-a-3158 |
| Free cholesterol in very large VLDL | 14 | 94795202 | rs9989237 | T | C | 0.20942 | NA | -0.0157 | 0.004932 | 0.0009 | met-d-XL_VLDL_FC |
| Cholesteryl esters in medium LDL | 14 | 94795202 | rs9989237 | T | C | 0.20942 | NA | -0.0161 | 0.005095 | 0.00091 | met-d-M_LDL_CE |
| Spleen volume | 14 | 94795202 | rs9989237 | T | C | 0.209552 | 32860 | -0.02833 | 0.008539 | 0.00091 | ebi-a-GCST90016667 |
| Cholesteryl esters in small VLDL | 14 | 94795202 | rs9989237 | T | C | 0.20942 | NA | -0.01595 | 0.005082 | 0.00093 | met-d-S_VLDL_CE |
| Nidogen-2 | 14 | 94795202 | rs9989237 | T | C | 0.21768 | 3301 | 0.0993 | 0.03 | 0.000933 | prot-a-2050 |
| CMP-N-acetylneuraminate-poly-alpha-2,8-sialyltransferase | 14 | 94795202 | rs9989237 | T | C | 0.21768 | 3301 | -0.0991 | 0.03 | 0.000955 | prot-a-2860 |
| Platelet crit | 14 | 94795202 | rs9989237 | T | C | 0.209416 | 350471 | -0.0092 | 0.002788 | 0.000965 | ukb-d-30090_irnt |
| NET100 0683 | 14 | 94795202 | rs9989237 | T | C | 0.21004 | 7916 | 0.063 | 0.0191 | 0.000977 | ubm-a-1839 |
| Free cholesterol in VLDL | 14 | 94795202 | rs9989237 | T | C | 0.20942 | NA | -0.01602 | 0.005052 | 0.00098 | met-d-VLDL_FC |
| Phospholipids in very large VLDL | 14 | 94795202 | rs9989237 | T | C | 0.20942 | NA | -0.01543 | 0.004914 | 0.001 | met-d-XL_VLDL_PL |
| Histone-lysine N-methyltransferase 2C | 14 | 94795202 | rs9989237 | T | C | 0.21768 | 3301 | 0.0986 | 0.03 | 0.001023 | prot-a-1676 |
| Galanin-like peptide | 14 | 94795202 | rs9989237 | T | C | 0.21768 | 3301 | 0.0987 | 0.03 | 0.001023 | prot-a-1174 |
| Fibroblast growth factor 20 | 14 | 94795202 | rs9989237 | T | C | 0.21768 | 3301 | 0.0985 | 0.03 | 0.001023 | prot-a-1092 |
| 3mm regularity index (left) | 14 | 94795202 | rs9989237 | T | C | 0.615 | NA | -0.07954 | 0.02429 | 0.001059 | ukb-e-5163_CSA |
| Apolipoprotein E (isoform E3) | 14 | 94795202 | rs9989237 | T | C | 0.21768 | 3301 | -0.0982 | 0.03 | 0.001072 | prot-a-131 |
| Neutrophil-activating peptide 2 | 14 | 94795202 | rs9989237 | T | C | 0.21768 | 3301 | 0.098 | 0.03 | 0.001096 | prot-a-2337 |
| Concentration of medium VLDL particles | 14 | 94795202 | rs9989237 | T | C | 0.20942 | NA | -0.01591 | 0.005076 | 0.0011 | met-d-M_VLDL_P |
| Phospholipids in large VLDL | 14 | 94795202 | rs9989237 | T | C | 0.20942 | NA | -0.01542 | 0.004913 | 0.0011 | met-d-L_VLDL_PL |
| Proteasome activator complex subunit 3 | 14 | 94795202 | rs9989237 | T | C | 0.21768 | 3301 | 0.0979 | 0.03 | 0.001122 | prot-a-2421 |
| volume BrainSegVolNotVent | 14 | 94795202 | rs9989237 | T | C | 0.21004 | 7916 | 0.0259 | 0.0079 | 0.001122 | ubm-a-2695 |
| 6mm weak meridian angle (right) | 14 | 94795202 | rs9989237 | T | C | 0.6112 | NA | -0.07684 | 0.02359 | 0.001128 | ukb-e-5101_CSA |
| Diseases of the musculoskeletal system and connective tissue | 14 | 94795202 | rs9989237 | T | C | 0.209437 | 361194 | -0.00382 | 0.001176 | 0.00116 | ukb-d-XIII_MUSCULOSKELET |
| Fasting insulin | 14 | 94795202 | rs9989237 | T | C | 0.083 | 16386 | 0.0273 | 0.0089 | 0.001169 | ebi-a-GCST90002239 |
| C-C motif chemokine 13 | 14 | 94795202 | rs9989237 | T | C | 0.21768 | 3301 | 0.0975 | 0.03 | 0.001175 | prot-a-389 |
| Corneodesmosin | 14 | 94795202 | rs9989237 | T | C | 0.21768 | 3301 | 0.0973 | 0.03 | 0.001202 | prot-a-500 |
| Caspase-2 | 14 | 94795202 | rs9989237 | T | C | 0.21768 | 3301 | 0.0972 | 0.03 | 0.001202 | prot-a-363 |
| Rheumatoid factor | 14 | 94795202 | rs9989237 | T | C | 0.20895 | 30565 | -0.63734 | 0.19681 | 0.001204 | ukb-d-30820_raw |
| SPARC | 14 | 94795202 | rs9989237 | T | C | 0.21768 | 3301 | 0.0971 | 0.03 | 0.00123 | prot-a-2809 |
| Caseinolytic peptidase B protein homolog | 14 | 94795202 | rs9989237 | T | C | 0.21768 | 3301 | -0.0971 | 0.03 | 0.00123 | prot-a-591 |
| Interleukin-9 | 14 | 94795202 | rs9989237 | T | C | 0.21768 | 3301 | 0.0968 | 0.03 | 0.001259 | prot-a-1545 |
| Phospholipids to total lipids ratio in very large VLDL | 14 | 94795202 | rs9989237 | T | C | 0.209295 | NA | -0.01656 | 0.005097 | 0.0013 | met-d-XL_VLDL_PL_pct |
| Triglycerides in medium VLDL | 14 | 94795202 | rs9989237 | T | C | 0.20942 | NA | -0.01521 | 0.004946 | 0.0013 | met-d-M_VLDL_TG |
| Ulcerative colitis | 14 | 94795202 | rs9989237 | T | C | NA | 45975 | 0.0618 | 0.0192 | 0.001322 | ebi-a-GCST004133 |
| Heat shock protein HSP 90-alpha/beta | 14 | 94795202 | rs9989237 | T | C | 0.21768 | 3301 | 0.0963 | 0.03 | 0.001349 | prot-a-1384 |
| Paclitaxel drug response (AUC) | 14 | 94795202 | rs9989237 | T | C | 0.206 | 164 | 0.474403 | 0.145711 | 0.001394 | ebi-a-GCST90011779 |
| Pancreas iron content | 14 | 94795202 | rs9989237 | T | C | 0.209919 | 25617 | 0.033948 | 0.010634 | 0.0014 | ebi-a-GCST90016676 |
| Duration to entering value | 14 | 94795202 | rs9989237 | T | C | 0.209597 | 113410 | -0.01632 | 0.005108 | 0.0014 | ukb-b-19585 |
| Tumor necrosis factor receptor superfamily member 16 | 14 | 94795202 | rs9989237 | T | C | 0.21768 | 3301 | -0.0955 | 0.03 | 0.001445 | prot-a-2043 |
| Protein SERAC1 | 14 | 94795202 | rs9989237 | T | C | 0.21768 | 3301 | -0.0954 | 0.03 | 0.001479 | prot-a-2686 |
| Ratio of omega-6 fatty acids to total fatty acids | 14 | 94795202 | rs9989237 | T | C | 0.209439 | NA | 0.016066 | 0.004952 | 0.0015 | met-d-Omega_6_pct |
| Total lipids in VLDL | 14 | 94795202 | rs9989237 | T | C | 0.20942 | NA | -0.01527 | 0.004991 | 0.0015 | met-d-VLDL_L |
| Total lipids in medium VLDL | 14 | 94795202 | rs9989237 | T | C | 0.20942 | NA | -0.0153 | 0.005055 | 0.0015 | met-d-M_VLDL_L |
| Total lipids in very large VLDL | 14 | 94795202 | rs9989237 | T | C | 0.20942 | NA | -0.01481 | 0.004899 | 0.0015 | met-d-XL_VLDL_L |
| Sex hormone-binding globulin levels adjusted for BMI | 14 | 94795202 | rs9989237 | T | C | 0.210051 | 188908 | -0.00545 | 0.001519 | 0.0015 | ebi-a-GCST90012106 |
| Bioavailable Testosterone | 14 | 94795202 | rs9989237 | T | C | 0.210539 | NA | 0.012794 | 0.004031 | 0.0015 | ieu-b-4869 |
| Beta-defensin 135 | 14 | 94795202 | rs9989237 | T | C | 0.21768 | 3301 | -0.0949 | 0.03 | 0.001549 | prot-a-802 |
| Interleukin-6 | 14 | 94795202 | rs9989237 | T | C | 0.21768 | 3301 | 0.0951 | 0.03 | 0.001549 | prot-a-1539 |
| Stromal cell-derived factor 2-like protein 1 | 14 | 94795202 | rs9989237 | T | C | 0.21768 | 3301 | -0.0951 | 0.03 | 0.001549 | prot-a-2656 |
| Total bilirubin | 14 | 94795202 | rs9989237 | T | C | 0.20971 | 342829 | -0.04022 | 0.01272 | 0.001568 | ukb-d-30840_raw |
| Non-cancer illness code self-reported: cholelithiasis/gall stones | 14 | 94795202 | rs9989237 | T | C | 0.209385 | 337159 | -0.0012 | 0.000381 | 0.001574 | ukb-a-71 |
| ETS domain-containing protein Elk-1 | 14 | 94795202 | rs9989237 | T | C | 0.21768 | 3301 | -0.0949 | 0.03 | 0.001585 | prot-a-930 |
| Concentration of VLDL particles | 14 | 94795202 | rs9989237 | T | C | 0.20942 | NA | -0.01528 | 0.005062 | 0.0016 | met-d-VLDL_P |
| Phospholipids in VLDL | 14 | 94795202 | rs9989237 | T | C | 0.20942 | NA | -0.01517 | 0.005028 | 0.0016 | met-d-VLDL_PL |
| Cholesterol in small VLDL | 14 | 94795202 | rs9989237 | T | C | 0.20942 | NA | -0.01527 | 0.005088 | 0.0016 | met-d-S_VLDL_C |
| TestASV_41 (Barnesiella) prevalence | 14 | 94795202 | rs9989237 | T | C | NA | 8956 | 0.246842 | 0.078256 | 0.001609 | ebi-a-GCST90011717 |
| Plasminogen activator inhibitor 1 | 14 | 94795202 | rs9989237 | T | C | 0.21768 | 3301 | 0.0946 | 0.03 | 0.001622 | prot-a-2696 |
| Angiopoietin-4 | 14 | 94795202 | rs9989237 | T | C | 0.21768 | 3301 | 0.0946 | 0.03 | 0.001622 | prot-a-95 |
| Submaxillary gland androgen-regulated protein 3B | 14 | 94795202 | rs9989237 | T | C | 0.21768 | 3301 | 0.0946 | 0.03 | 0.001622 | prot-a-2783 |
| NET100 0254 | 14 | 94795202 | rs9989237 | T | C | 0.21004 | 7916 | -0.0603 | 0.0192 | 0.00166 | ubm-a-1410 |
| Vitamin B12 | 14 | 94795202 | rs9989237 | T | C | 0.6217 | NA | 0.1293 | 0.04113 | 0.001664 | ukb-e-100013_CSA |
| Amyloid beta A4 protein | 14 | 94795202 | rs9989237 | T | C | 0.21768 | 3301 | 0.0941 | 0.03 | 0.001738 | prot-a-139 |
| NET100 1387 | 14 | 94795202 | rs9989237 | T | C | 0.21004 | 7916 | -0.0607 | 0.0194 | 0.001738 | ubm-a-2543 |
| Histone-lysine N-methyltransferase SETD2 | 14 | 94795202 | rs9989237 | T | C | 0.21768 | 3301 | 0.0938 | 0.03 | 0.001778 | prot-a-2703 |
| Free cholesterol in chylomicrons and extremely large VLDL | 14 | 94795202 | rs9989237 | T | C | 0.20942 | NA | -0.01478 | 0.004906 | 0.0018 | met-d-XXL_VLDL_FC |
| Phospholipids in small VLDL | 14 | 94795202 | rs9989237 | T | C | 0.20942 | NA | -0.01489 | 0.005079 | 0.0018 | met-d-S_VLDL_PL |
| WAP four-disulfide core domain protein 13 | 14 | 94795202 | rs9989237 | T | C | 0.21768 | 3301 | 0.0935 | 0.03 | 0.00182 | prot-a-3223 |
| Triglycerides in small HDL | 14 | 94795202 | rs9989237 | T | C | 0.20942 | NA | -0.01498 | 0.004939 | 0.0019 | met-d-S_HDL_TG |
| Interleukin-18 receptor 1 | 14 | 94795202 | rs9989237 | T | C | 0.21768 | 3301 | -0.0931 | 0.03 | 0.001905 | prot-a-1492 |
| volume SupraTentorialVolNotVentVox | 14 | 94795202 | rs9989237 | T | C | 0.21004 | 7916 | 0.0256 | 0.0082 | 0.001905 | ubm-a-2707 |
| Arylsulfatase A | 14 | 94795202 | rs9989237 | T | C | 0.21768 | 3301 | 0.0929 | 0.03 | 0.00195 | prot-a-171 |
| Centromere protein W | 14 | 94795202 | rs9989237 | T | C | 0.21768 | 3301 | 0.0927 | 0.03 | 0.001995 | prot-a-511 |
| Cholesterol in medium LDL | 14 | 94795202 | rs9989237 | T | C | 0.20942 | NA | -0.01487 | 0.005098 | 0.002 | met-d-M_LDL_C |
| Lifetime number of sexual partners | 14 | 94795202 | rs9989237 | T | C | 0.6071 | NA | -0.04612 | 0.01493 | 0.002015 | ukb-e-2149_CSA |
| CD25 on IgD+ CD38- naive B cell | 14 | 94795202 | rs9989237 | T | C | 0.2687 | 1835 | 0.1208 | 0.0391 | 0.00203 | ebi-a-GCST90001781 |
| Glucagon-like peptide 1 receptor | 14 | 94795202 | rs9989237 | T | C | 0.21768 | 3301 | 0.0926 | 0.03 | 0.002042 | prot-a-1219 |
| Triglycerides in very large VLDL | 14 | 94795202 | rs9989237 | T | C | 0.20942 | NA | -0.0143 | 0.00487 | 0.0021 | met-d-XL_VLDL_TG |
| Ratio of polyunsaturated fatty acids to total fatty acids | 14 | 94795202 | rs9989237 | T | C | 0.209439 | NA | 0.015326 | 0.004897 | 0.0021 | met-d-PUFA_pct |
| Elafin levels | 14 | 94795202 | rs9989237 | T | C | 0.234 | 1322 | 0.142542 | 0.046193 | 0.002136 | ebi-a-GCST90010250 |
| Beta-1,4-galactosyltransferase 7 | 14 | 94795202 | rs9989237 | T | C | 0.21768 | 3301 | 0.0923 | 0.03 | 0.002138 | prot-a-221 |
| Other reactioin to severe stress, and adjustment disorders | 14 | 94795202 | rs9989237 | T | C | 0.2082 | NA | 0.0662 | 0.0216 | 0.00218 | finn-b-F5_STRESSOTH |
| Triglycerides in VLDL | 14 | 94795202 | rs9989237 | T | C | 0.20942 | NA | -0.01439 | 0.0049 | 0.0022 | met-d-VLDL_TG |
| Operation code: colonoscopy/sigmoidoscopy | 14 | 94795202 | rs9989237 | T | C | 0.209974 | 462933 | -0.00109 | 0.000355 | 0.0022 | ukb-b-2218 |
| Usual side of head for mobile phone use | 14 | 94795202 | rs9989237 | T | C | 0.6124 | NA | 0.1805 | 0.05904 | 0.002237 | ukb-e-1150_p3_CSA |
| NoneX | 14 | 94795202 | rs9989237 | T | C | 0.21768 | 3301 | 0.0918 | 0.03 | 0.002239 | prot-a-2065 |
| Interferon alpha-6 | 14 | 94795202 | rs9989237 | T | C | 0.21768 | 3301 | 0.0919 | 0.03 | 0.002239 | prot-a-1423 |
| Methyl-CpG-binding domain protein 4 | 14 | 94795202 | rs9989237 | T | C | 0.21768 | 3301 | 0.0916 | 0.03 | 0.002291 | prot-a-1862 |
| Atrial natriuretic factor | 14 | 94795202 | rs9989237 | T | C | 0.21768 | 3301 | 0.0914 | 0.03 | 0.002344 | prot-a-2076 |
| IDP T1 FAST ROIs L supracalc cortex | 14 | 94795202 | rs9989237 | T | C | 0.21004 | 7916 | 0.0508 | 0.0167 | 0.002344 | ubm-a-118 |
| DNA-binding protein SATB1 | 14 | 94795202 | rs9989237 | T | C | 0.21768 | 3301 | 0.0912 | 0.03 | 0.002399 | prot-a-2630 |
| Prolactin | 14 | 94795202 | rs9989237 | T | C | 0.21768 | 3301 | 0.0912 | 0.03 | 0.002399 | prot-a-2375 |
| Total lipids in medium LDL | 14 | 94795202 | rs9989237 | T | C | 0.20942 | NA | -0.01463 | 0.005096 | 0.0024 | met-d-M_LDL_L |
| Free cholesterol to total lipids ratio in large HDL | 14 | 94795202 | rs9989237 | T | C | 0.20942 | NA | 0.013321 | 0.004732 | 0.0024 | met-d-L_HDL_FC_pct |
| Diabethic neuropathy | 14 | 94795202 | rs9989237 | T | C | 0.208 | NA | -0.1435 | 0.0473 | 0.002423 | finn-b-G6_DIABETNEUR |
| Ras-related protein Rab-27A | 14 | 94795202 | rs9989237 | T | C | 0.21768 | 3301 | 0.0909 | 0.03 | 0.002455 | prot-a-2474 |
| Group IIE secretory phospholipase A2 | 14 | 94795202 | rs9989237 | T | C | 0.21768 | 3301 | 0.091 | 0.03 | 0.002455 | prot-a-2289 |
| Fibroblast growth factor 23 | 14 | 94795202 | rs9989237 | T | C | 0.21768 | 3301 | 0.091 | 0.03 | 0.002455 | prot-a-1093 |
| Glutamate carboxypeptidase 2 | 14 | 94795202 | rs9989237 | T | C | 0.21768 | 3301 | 0.0909 | 0.03 | 0.002455 | prot-a-1135 |
| Phospholipids in very large HDL | 14 | 94795202 | rs9989237 | T | C | 0.20942 | NA | 0.012274 | 0.004579 | 0.0025 | met-d-XL_HDL_PL |
| Diagnoses - main ICD10: L72.0 Epidermal cyst | 14 | 94795202 | rs9989237 | T | C | 0.209969 | 463010 | -0.00049 | 0.000161 | 0.0025 | ukb-b-15003 |
| Vesicle-associated membrane protein 8 | 14 | 94795202 | rs9989237 | T | C | 0.21768 | 3301 | -0.0906 | 0.03 | 0.002512 | prot-a-3188 |
| Hephaestin-like protein 1 | 14 | 94795202 | rs9989237 | T | C | 0.21768 | 3301 | 0.0904 | 0.03 | 0.00257 | prot-a-1328 |
| Main speciality of consultant (recoded): Trauma and orthopaedics | 14 | 94795202 | rs9989237 | T | C | 0.209962 | 461797 | -0.00277 | 0.000917 | 0.0026 | ukb-b-16399 |
| Cullin-4B | 14 | 94795202 | rs9989237 | T | C | 0.21768 | 3301 | 0.0903 | 0.03 | 0.00263 | prot-a-733 |
| Triglycerides in large VLDL | 14 | 94795202 | rs9989237 | T | C | 0.20942 | NA | -0.01389 | 0.004898 | 0.0027 | met-d-L_VLDL_TG |
| kallikrein related peptidase 11 | 14 | 94795202 | rs9989237 | T | C | 0.2384 | 3394 | 0.0942 | 0.0314 | 0.00272 | prot-b-8 |
| Treatment speciality of consultant (recoded) | 14 | 94795202 | rs9989237 | T | C | 0.7489 | NA | -0.3584 | 0.1197 | 0.002748 | ukb-e-41246_p71_MID |
| Brain-derived neurotrophic factor | 14 | 94795202 | rs9989237 | T | C | 0.21768 | 3301 | 0.09 | 0.03 | 0.002754 | prot-a-242 |
| Oral and oropharyngeal cancer | 14 | 94795202 | rs9989237 | T | C | 0.209926 | 372855 | 0.000403 | 0.000135 | 0.0028 | ieu-b-4962 |
| Protein transport protein Sec61 subunit gamma | 14 | 94795202 | rs9989237 | T | C | 0.21768 | 3301 | 0.0898 | 0.03 | 0.002818 | prot-a-2662 |
| CD20- B cell %B cell | 14 | 94795202 | rs9989237 | T | C | 0.2675 | 3658 | 0.08512 | 0.0285 | 0.002838 | ebi-a-GCST90001419 |
| TestASV_23 (Barnesiella) abundance | 14 | 94795202 | rs9989237 | T | C | NA | 8956 | 0.158749 | 0.053259 | 0.002876 | ebi-a-GCST90011500 |
| Secretoglobin family 1D member 2 | 14 | 94795202 | rs9989237 | T | C | 0.21768 | 3301 | -0.0895 | 0.03 | 0.002884 | prot-a-2647 |
| Isochorismatase domain-containing protein 1 | 14 | 94795202 | rs9989237 | T | C | 0.21768 | 3301 | 0.0896 | 0.03 | 0.002884 | prot-a-1578 |
| Total triglycerides | 14 | 94795202 | rs9989237 | T | C | 0.20942 | NA | -0.01424 | 0.004929 | 0.0029 | met-d-Total_TG |
| Triglycerides to total lipids ratio in large VLDL | 14 | 94795202 | rs9989237 | T | C | 0.209427 | NA | 0.014391 | 0.005064 | 0.0029 | met-d-L_VLDL_TG_pct |
| Phospholipids in medium VLDL | 14 | 94795202 | rs9989237 | T | C | 0.20942 | NA | -0.01449 | 0.005081 | 0.0029 | met-d-M_VLDL_PL |
| Concentration of small HDL particles | 14 | 94795202 | rs9989237 | T | C | 0.20942 | NA | -0.01525 | 0.005089 | 0.0029 | met-d-S_HDL_P |
| resistin | 14 | 94795202 | rs9989237 | T | C | 0.2384 | 3394 | -0.0936 | 0.0314 | 0.002912 | prot-b-32 |
| Burn and corrosion of trunk | 14 | 94795202 | rs9989237 | T | C | 0.2083 | NA | 0.2721 | 0.0914 | 0.00292 | finn-b-ST19_BURN_CORROSION_TRUNK |
| Persulfide dioxygenase ETHE1, mitochondrial | 14 | 94795202 | rs9989237 | T | C | 0.21768 | 3301 | -0.0892 | 0.03 | 0.002951 | prot-a-992 |
| Cholesterol in very large VLDL | 14 | 94795202 | rs9989237 | T | C | 0.20942 | NA | -0.01411 | 0.004972 | 0.003 | met-d-XL_VLDL_C |
| Triglycerides in small VLDL | 14 | 94795202 | rs9989237 | T | C | 0.20942 | NA | -0.01416 | 0.004942 | 0.003 | met-d-S_VLDL_TG |
| MAP kinase-activated protein kinase 5 | 14 | 94795202 | rs9989237 | T | C | 0.21768 | 3301 | 0.0891 | 0.03 | 0.00302 | prot-a-1853 |
| Index of best refractometry result (left) | 14 | 94795202 | rs9989237 | T | C | 0.7946 | NA | -0.07518 | 0.02537 | 0.003042 | ukb-e-5276_AFR |
| 3-hydroxybutyrate | 14 | 94795202 | rs9989237 | T | C | 0.213589 | 24151 | 0.033546 | 0.011321 | 0.003055 | met-c-846 |
| Endoplasmic reticulum resident protein 44 | 14 | 94795202 | rs9989237 | T | C | 0.21768 | 3301 | 0.0889 | 0.03 | 0.00309 | prot-a-985 |
| Paired box protein Pax-4 | 14 | 94795202 | rs9989237 | T | C | 0.21768 | 3301 | 0.0886 | 0.03 | 0.003162 | prot-a-2188 |
| a2009s lh S precentral-inf-part thickness | 14 | 94795202 | rs9989237 | T | C | 0.21004 | 7916 | -0.0506 | 0.0172 | 0.003162 | ubm-a-3026 |
| Injury of muscle and tendon at hip and thigh level | 14 | 94795202 | rs9989237 | T | C | 0.2083 | NA | -0.1608 | 0.0545 | 0.003169 | finn-b-ST19_INJURY_MUSCLE_TENDON_HIP_THIGH_LEVEL |
| ENSG00000165948 | 14 | 94795202 | rs9989237 | T | C | 0.22464 | 24676 | -0.04198 | 0.014257 | 0.00323 | eqtl-a-ENSG00000165948 |
| Amenorrhoea | 14 | 94795202 | rs9989237 | T | C | 0.2069 | NA | 0.1516 | 0.0515 | 0.003239 | finn-b-N14_AMENORRHEAPRIM |
| Serpin B8 levels | 14 | 94795202 | rs9989237 | T | C | 0.237 | 1254 | 0.141703 | 0.047844 | 0.003255 | ebi-a-GCST90010345 |
| Triglycerides to total lipids ratio in large HDL | 14 | 94795202 | rs9989237 | T | C | 0.20942 | NA | -0.01365 | 0.004808 | 0.0033 | met-d-L_HDL_TG_pct |
| cubilin | 14 | 94795202 | rs9989237 | T | C | 0.21768 | 3301 | 0.0883 | 0.03 | 0.003311 | prot-a-731 |
| SUN domain-containing protein 5 | 14 | 94795202 | rs9989237 | T | C | 0.21768 | 3301 | 0.0882 | 0.03 | 0.003311 | prot-a-2899 |
| Low-density lipoprotein receptor | 14 | 94795202 | rs9989237 | T | C | 0.21768 | 3301 | 0.0882 | 0.03 | 0.003311 | prot-a-1717 |
| Disintegrin and metalloproteinase domain-containing protein 12 | 14 | 94795202 | rs9989237 | T | C | 0.21768 | 3301 | 0.0881 | 0.03 | 0.003388 | prot-a-26 |
| Fish consumer | 14 | 94795202 | rs9989237 | T | C | 0.6357 | NA | 0.291 | 0.09953 | 0.003459 | ukb-e-103140_CSA |
| Interleukin-12 | 14 | 94795202 | rs9989237 | T | C | 0.21768 | 3301 | 0.0879 | 0.03 | 0.003467 | prot-a-1470 |
| Cholesteryl esters in small LDL | 14 | 94795202 | rs9989237 | T | C | 0.20942 | NA | -0.01418 | 0.005091 | 0.0035 | met-d-S_LDL_CE |
| Offspring birth weight | 14 | 94795202 | rs9989237 | T | C | 0.21 | 68258 | -0.023 | 0.0079 | 0.0035 | ebi-a-GCST005314 |
| Protein G6b | 14 | 94795202 | rs9989237 | T | C | 0.21768 | 3301 | 0.0875 | 0.03 | 0.003548 | prot-a-1159 |
| CDGSH iron-sulfur domain-containing protein 2 | 14 | 94795202 | rs9989237 | T | C | 0.21768 | 3301 | 0.0876 | 0.03 | 0.003548 | prot-a-563 |
| Cysteine-rich motor neuron 1 protein | 14 | 94795202 | rs9989237 | T | C | 0.21768 | 3301 | 0.0876 | 0.03 | 0.003548 | prot-a-660 |
| IGF-1 | 14 | 94795202 | rs9989237 | T | C | 0.2096 | 342439 | 0.008303 | 0.002851 | 0.003583 | ukb-d-30770_irnt |
| Ehlers-Danlos syndrome | 14 | 94795202 | rs9989237 | T | C | 0.2083 | NA | 0.4821 | 0.1656 | 0.003592 | finn-b-Q17_EHLER_SYNDR |
| Bipolar disorder vs Tourette's syndrome and other tic disorders (ordinary least squares (OLS)) | 14 | 94795202 | rs9989237 | T | C | NA | 25171 | -0.011 | 0.0039 | 0.0036 | ebi-a-GCST90016609 |
| Underlying (primary) cause of death: ICD10: J84.1 Other interstitial pulmonary diseases with fibrosis | 14 | 94795202 | rs9989237 | T | C | 0.209385 | 7637 | -0.00639 | 0.002194 | 0.003602 | ukb-a-358 |
| Diamine acetyltransferase 2 | 14 | 94795202 | rs9989237 | T | C | 0.21768 | 3301 | -0.0873 | 0.03 | 0.003631 | prot-a-2629 |
| Protein-tyrosine sulfotransferase 1 | 14 | 94795202 | rs9989237 | T | C | 0.21768 | 3301 | -0.0874 | 0.03 | 0.003631 | prot-a-3092 |
| C-reactive protein | 14 | 94795202 | rs9989237 | T | C | 0.5781 | NA | -0.08113 | 0.02795 | 0.003697 | ukb-e-30710_EAS |
| VLDL cholesterol | 14 | 94795202 | rs9989237 | T | C | 0.20942 | NA | -0.01418 | 0.005082 | 0.0037 | met-d-VLDL_C |
| Phospholipids to total lipids ratio in chylomicrons and extremely large VLDL | 14 | 94795202 | rs9989237 | T | C | 0.209453 | NA | -0.01449 | 0.005118 | 0.0037 | met-d-XXL_VLDL_PL_pct |
| Free cholesterol in medium VLDL | 14 | 94795202 | rs9989237 | T | C | 0.20942 | NA | -0.01425 | 0.005088 | 0.0037 | met-d-M_VLDL_FC |
| Glutathione S-transferase Mu 1 | 14 | 94795202 | rs9989237 | T | C | 0.21768 | 3301 | -0.0871 | 0.03 | 0.003715 | prot-a-1287 |
| Monounsaturated fatty acids | 14 | 94795202 | rs9989237 | T | C | 0.209439 | NA | -0.01432 | 0.005039 | 0.0038 | met-d-MUFA |
| Interferon alpha-10 | 14 | 94795202 | rs9989237 | T | C | 0.21768 | 3301 | -0.087 | 0.03 | 0.003802 | prot-a-1418 |
| Chorionic somatomammotropin hormone | 14 | 94795202 | rs9989237 | T | C | 0.21768 | 3301 | -0.0869 | 0.03 | 0.003802 | prot-a-688 |
| Treatment speciality of consultant (recoded) | 14 | 94795202 | rs9989237 | T | C | 0.7735 | NA | 0.2722 | 0.09412 | 0.003824 | ukb-e-41246_p26_MID |
| E3 ubiquitin-protein ligase ZNRF3 | 14 | 94795202 | rs9989237 | T | C | 0.21768 | 3301 | 0.0867 | 0.03 | 0.00389 | prot-a-3278 |
| Leukocyte-associated immunoglobulin-like receptor 1 | 14 | 94795202 | rs9989237 | T | C | 0.21768 | 3301 | 0.0866 | 0.03 | 0.00389 | prot-a-1694 |
| Fractures | 14 | 94795202 | rs9989237 | T | C | NA | 426795 | 4.82457 | -2.46529 | 0.0039 | ebi-a-GCST006980 |
| Ragulator complex protein LAMTOR3 | 14 | 94795202 | rs9989237 | T | C | 0.21768 | 3301 | -0.0865 | 0.03 | 0.003981 | prot-a-1699 |
| Ratio of linoleic acid to total fatty acids | 14 | 94795202 | rs9989237 | T | C | 0.209439 | NA | 0.014514 | 0.004977 | 0.004 | met-d-LA_pct |
| Bipolar disorder vs autism spectrum disorder (ordinary least squares (OLS)) | 14 | 94795202 | rs9989237 | T | C | NA | 38733 | -0.0093 | 0.0032 | 0.004 | ebi-a-GCST90016606 |
| Lysosome membrane protein 2 | 14 | 94795202 | rs9989237 | T | C | 0.21768 | 3301 | 0.0862 | 0.03 | 0.004074 | prot-a-2640 |
| C-C motif chemokine 26 | 14 | 94795202 | rs9989237 | T | C | 0.21768 | 3301 | 0.0862 | 0.03 | 0.004074 | prot-a-404 |
| [Pyruvate dehydrogenase (acetyl-transferring)] kinase isozyme 2, mitochondrial | 14 | 94795202 | rs9989237 | T | C | 0.21768 | 3301 | 0.0862 | 0.03 | 0.004074 | prot-a-2236 |
| a2009s lh G&S occipital inf area | 14 | 94795202 | rs9989237 | T | C | 0.21004 | 7916 | 0.0478 | 0.0166 | 0.004074 | ubm-a-2748 |
| Cholesterol to total lipids ratio in small LDL | 14 | 94795202 | rs9989237 | T | C | 0.20942 | NA | -0.01475 | 0.005108 | 0.0041 | met-d-S_LDL_C_pct |
| OTU99_55 (Barnesiella) abundance | 14 | 94795202 | rs9989237 | T | C | NA | 8956 | 0.097874 | 0.034109 | 0.004112 | ebi-a-GCST90011464 |
| Chlocystitis | 14 | 94795202 | rs9989237 | T | C | 0.2084 | NA | -0.1129 | 0.0394 | 0.004123 | finn-b-K11_CHOLECYST |
| Thromboxane-A synthase | 14 | 94795202 | rs9989237 | T | C | 0.21768 | 3301 | -0.0861 | 0.03 | 0.004169 | prot-a-2937 |
| Redox-regulatory protein FAM213A | 14 | 94795202 | rs9989237 | T | C | 0.21768 | 3301 | -0.0861 | 0.03 | 0.004169 | prot-a-1047 |
| Tumor necrosis factor receptor 2 levels | 14 | 94795202 | rs9989237 | T | C | 0.2198 | 21758 | 0.032 | 0.0112 | 0.004191 | ebi-a-GCST90012026 |
| Excessive, freguent and irrelgular menstruation | 14 | 94795202 | rs9989237 | T | C | 0.2078 | NA | 0.0452 | 0.0158 | 0.004232 | finn-b-N14_MESNRUIRREG |
| Acid sphingomyelinase-like phosphodiesterase 3a | 14 | 94795202 | rs9989237 | T | C | 0.21768 | 3301 | 0.0858 | 0.03 | 0.004266 | prot-a-2781 |
| Total lipids in very large HDL | 14 | 94795202 | rs9989237 | T | C | 0.20942 | NA | 0.011544 | 0.004578 | 0.0043 | met-d-XL_HDL_L |
| Main speciality of consultant (recoded) | 14 | 94795202 | rs9989237 | T | C | 0.7714 | NA | 0.2737 | 0.09595 | 0.004329 | ukb-e-41245_p20_MID |
| Endocrine, nutritional and metabolic diseases | 14 | 94795202 | rs9989237 | T | C | 0.209437 | 361194 | -0.00115 | 0.000404 | 0.004336 | ukb-d-IV_ENDOCRIN_NUTRIT |
| Bran cereal intake | 14 | 94795202 | rs9989237 | T | C | 0.7781 | NA | -0.0534 | 0.01874 | 0.00438 | ukb-e-100840_AFR |
| Treatment speciality of consultant (recoded): Trauma & orthopaedics | 14 | 94795202 | rs9989237 | T | C | 0.209953 | 448516 | -0.00275 | 0.000967 | 0.0044 | ukb-b-8667 |
| Used an inhaler for chest within last hour | 14 | 94795202 | rs9989237 | T | C | 0.209385 | 309688 | -0.00075 | 0.000265 | 0.004401 | ukb-a-339 |
| Direct bilirubin | 14 | 94795202 | rs9989237 | T | C | 0.20949 | 292933 | -0.00759 | 0.00267 | 0.004446 | ukb-d-30660_raw |
| Pneumonia derived septichemia (ASTHMA_SEPTICHE_PNEUMONIA) | 14 | 94795202 | rs9989237 | T | C | 0.2082 | NA | 0.2297 | 0.0808 | 0.00446 | finn-b-ASTHMA_SEPTICHE_PNEUMONIA |
| Pneumonia derived septichemia (SEPTICHE_PNEUMONIA) | 14 | 94795202 | rs9989237 | T | C | 0.2082 | NA | 0.2297 | 0.0808 | 0.00446 | finn-b-SEPTICHE_PNEUMONIA |
| Platelet-derived growth factor subunit B | 14 | 94795202 | rs9989237 | T | C | 0.21768 | 3301 | 0.0855 | 0.03 | 0.004467 | prot-a-2226 |
| Illnesses of siblings | 14 | 94795202 | rs9989237 | T | C | 0.8005 | NA | 0.1497 | 0.05267 | 0.004467 | ukb-e-20111_p13_AFR |
| Number of symbol digit matches attempted | 14 | 94795202 | rs9989237 | T | C | 0.209624 | 113106 | 0.014557 | 0.005121 | 0.0045 | ukb-b-1806 |
| a2009s rh G occipital middle area | 14 | 94795202 | rs9989237 | T | C | 0.21004 | 7916 | 0.045 | 0.0159 | 0.004571 | ubm-a-2871 |
| Achilles tendinitis | 14 | 94795202 | rs9989237 | T | C | 0.2078 | NA | 0.1249 | 0.0442 | 0.004666 | finn-b-M13_ACHILLESTEND |
| Transmembrane protein 2 | 14 | 94795202 | rs9989237 | T | C | 0.21768 | 3301 | -0.0849 | 0.03 | 0.004677 | prot-a-3009 |
| Cholesteryl esters in small HDL | 14 | 94795202 | rs9989237 | T | C | 0.20942 | NA | -0.01403 | 0.005096 | 0.0047 | met-d-S_HDL_CE |
| Enteropeptidase | 14 | 94795202 | rs9989237 | T | C | 0.21768 | 3301 | 0.0847 | 0.03 | 0.004786 | prot-a-3025 |
| Peroxisome proliferator-activated receptor alpha | 14 | 94795202 | rs9989237 | T | C | 0.21768 | 3301 | 0.0848 | 0.03 | 0.004786 | prot-a-2336 |
| Semaphorin-7A | 14 | 94795202 | rs9989237 | T | C | 0.21768 | 3301 | -0.0847 | 0.03 | 0.004786 | prot-a-2680 |
| Phospholipids in chylomicrons and extremely large VLDL | 14 | 94795202 | rs9989237 | T | C | 0.20942 | NA | -0.01322 | 0.004888 | 0.0048 | met-d-XXL_VLDL_PL |
| TestASV_47 (Parasutterella) prevalence | 14 | 94795202 | rs9989237 | T | C | NA | 8956 | -0.21824 | 0.077399 | 0.004807 | ebi-a-GCST90011723 |
| Inflammatory disease of cervix uteri | 14 | 94795202 | rs9989237 | T | C | 0.2079 | NA | -0.1513 | 0.0537 | 0.004817 | finn-b-N14_INFCERVIX |
| Examination and observation for other reasons | 14 | 94795202 | rs9989237 | T | C | 0.2079 | NA | 0.0468 | 0.0166 | 0.004873 | finn-b-Z21_EXAM_OBSER_OTH_REASO |
| Ubiquitin thioesterase OTUB2 | 14 | 94795202 | rs9989237 | T | C | 0.21768 | 3301 | 0.0845 | 0.03 | 0.004898 | prot-a-2158 |
| Transmembrane protein C16orf54 | 14 | 94795202 | rs9989237 | T | C | 0.21768 | 3301 | 0.0844 | 0.03 | 0.004898 | prot-a-285 |
| Free cholesterol in small VLDL | 14 | 94795202 | rs9989237 | T | C | 0.20942 | NA | -0.01358 | 0.005089 | 0.0049 | met-d-S_VLDL_FC |
| Sialomucin core protein 24 levels | 14 | 94795202 | rs9989237 | T | C | 0.235 | 1313 | 0.131458 | 0.046577 | 0.004933 | ebi-a-GCST90010291 |
| Burns classified according to extent of body surface involved | 14 | 94795202 | rs9989237 | T | C | 0.2083 | NA | 0.4305 | 0.1532 | 0.004947 | finn-b-ST19_BURNS_CLASSIFIED_ACCOR_EXTENT_BODY_SURFA_INVOLVED |
| Activated partial thromboplastin time | 14 | 94795202 | rs9989237 | T | C | 0.389 | NA | -0.02112 | 0.007516 | 0.00495 | bbj-a-7 |
| triglycerides | 14 | 94795202 | rs9989237 | T | C | 0.210174 | NA | -0.00686 | 0.002442 | 0.005 | ieu-b-111 |
| Diagnoses - main ICD10: J18.1 Lobar pneumonia, unspecified | 14 | 94795202 | rs9989237 | T | C | 0.209969 | 463010 | 0.00051 | 0.000182 | 0.005 | ukb-b-6576 |
| Place of birth in UK - north co-ordinate | 14 | 94795202 | rs9989237 | T | C | 0.209577 | 434576 | 0.005708 | 0.002032 | 0.005 | ukb-b-1431 |
| P2X purinoceptor 6 | 14 | 94795202 | rs9989237 | T | C | 0.21768 | 3301 | 0.0843 | 0.03 | 0.005012 | prot-a-2160 |
| NET100 0692 | 14 | 94795202 | rs9989237 | T | C | 0.21004 | 7916 | 0.0544 | 0.0194 | 0.005012 | ubm-a-1848 |
| DKTatlas rh inferiorparietal area | 14 | 94795202 | rs9989237 | T | C | 0.21004 | 7916 | 0.0409 | 0.0146 | 0.005012 | ubm-a-2826 |
| Follistatin levels | 14 | 94795202 | rs9989237 | T | C | 0.2184 | 21758 | -0.0359 | 0.0128 | 0.005069 | ebi-a-GCST90012080 |
| IGF-1 | 14 | 94795202 | rs9989237 | T | C | 0.2096 | 342439 | 0.045297 | 0.016169 | 0.005086 | ukb-d-30770_raw |
| Transmembrane emp24 domain-containing protein 4 | 14 | 94795202 | rs9989237 | T | C | 0.21768 | 3301 | 0.0842 | 0.03 | 0.005129 | prot-a-2995 |
| Transmembrane and coiled-coil domain-containing protein 5A | 14 | 94795202 | rs9989237 | T | C | 0.21768 | 3301 | 0.084 | 0.03 | 0.005129 | prot-a-2992 |
| B-cell lymphoma/leukemia 10 | 14 | 94795202 | rs9989237 | T | C | 0.21768 | 3301 | -0.084 | 0.03 | 0.005129 | prot-a-235 |
| Diagnoses - secondary ICD10: N92.0 Excessive and frequent menstruation with regular cycle | 14 | 94795202 | rs9989237 | T | C | 0.209969 | 463010 | 0.000542 | 0.000194 | 0.0052 | ukb-b-12282 |
| Plasmin | 14 | 94795202 | rs9989237 | T | C | 0.21768 | 3301 | 0.0838 | 0.03 | 0.005248 | prot-a-2302 |
| NET100 1091 | 14 | 94795202 | rs9989237 | T | C | 0.21004 | 7916 | 0.0539 | 0.0193 | 0.005248 | ubm-a-2247 |
| Trochanteric bursitis | 14 | 94795202 | rs9989237 | T | C | 0.2077 | NA | 0.1247 | 0.0447 | 0.005256 | finn-b-M13_TROCHANTERICBURSITIS |
| Job involves shift work | 14 | 94795202 | rs9989237 | T | C | 0.7919 | NA | 0.08788 | 0.0315 | 0.005268 | ukb-e-826_AFR |
| Prolyl 4-hydroxylase subunit alpha-1 | 14 | 94795202 | rs9989237 | T | C | 0.21768 | 3301 | 0.0835 | 0.03 | 0.00537 | prot-a-2161 |
| Regulator of microtubule dynamics protein 3 | 14 | 94795202 | rs9989237 | T | C | 0.21768 | 3301 | 0.0836 | 0.03 | 0.00537 | prot-a-2550 |
| NODEamps100 0001 | 14 | 94795202 | rs9989237 | T | C | 0.21004 | 7916 | 0.0514 | 0.0184 | 0.00537 | ubm-a-892 |
| OTU99_65 (Sutterellaceae) prevalence | 14 | 94795202 | rs9989237 | T | C | NA | 8956 | -0.21513 | 0.077287 | 0.005376 | ebi-a-GCST90011671 |
| OTU97_58 (Sutterellaceae) prevalence | 14 | 94795202 | rs9989237 | T | C | NA | 8956 | -0.21513 | 0.077287 | 0.005376 | ebi-a-GCST90011606 |
| Concentration of small LDL particles | 14 | 94795202 | rs9989237 | T | C | 0.20942 | NA | -0.01355 | 0.005093 | 0.0054 | met-d-S_LDL_P |
| Gut microbiota abundance (family Family XIII id.1957) | 14 | 94795202 | rs9989237 | T | C | NA | 14306 | -0.03698 | 0.013185 | 0.005438 | ebi-a-GCST90016939 |
| NET100 0750 | 14 | 94795202 | rs9989237 | T | C | 0.21004 | 7916 | 0.0533 | 0.0192 | 0.005495 | ubm-a-1906 |
| a2009s rh G front inf-Opercular area | 14 | 94795202 | rs9989237 | T | C | 0.21004 | 7916 | 0.0456 | 0.0164 | 0.005495 | ubm-a-2864 |
| Triglycerides in medium LDL | 14 | 94795202 | rs9989237 | T | C | 0.20942 | NA | -0.01347 | 0.005016 | 0.0055 | met-d-M_LDL_TG |
| Postprocedural disorders of nervous system | 14 | 94795202 | rs9989237 | T | C | 0.2083 | NA | 0.1523 | 0.0549 | 0.005508 | finn-b-G6_POSTOPNEURO |
| Other and unspecified mental retardation | 14 | 94795202 | rs9989237 | T | C | 0.2082 | NA | -0.3183 | 0.1149 | 0.005582 | finn-b-F5_OTHRET |
| Ratio of triglycerides to phosphoglycerides | 14 | 94795202 | rs9989237 | T | C | 0.209439 | NA | -0.01282 | 0.004727 | 0.0056 | met-d-TG_by_PG |
| a2009s lh G cingul-Post-dorsal thickness | 14 | 94795202 | rs9989237 | T | C | 0.21004 | 7916 | -0.0492 | 0.0178 | 0.005754 | ubm-a-2967 |
| Cholesteryl esters in very large HDL | 14 | 94795202 | rs9989237 | T | C | 0.20942 | NA | 0.011337 | 0.004563 | 0.0058 | met-d-XL_HDL_CE |
| Platelet distribution width | 14 | 94795202 | rs9989237 | T | C | 0.7908 | NA | 0.07339 | 0.02661 | 0.005808 | ukb-e-30110_AFR |
| Ever smoked | 14 | 94795202 | rs9989237 | T | C | NA | 108736 | 0.0071 | 0.0026 | 0.005851 | ieu-b-4858 |
| Killer cell lectin-like receptor subfamily F member 1 | 14 | 94795202 | rs9989237 | T | C | 0.21768 | 3301 | 0.0828 | 0.03 | 0.005888 | prot-a-1672 |
| L-lactate dehydrogenase C chain | 14 | 94795202 | rs9989237 | T | C | 0.21768 | 3301 | 0.0827 | 0.03 | 0.005888 | prot-a-1716 |
| Neutrophil cytosol factor 2 | 14 | 94795202 | rs9989237 | T | C | 0.21768 | 3301 | -0.0827 | 0.03 | 0.005888 | prot-a-2009 |
| Alpha-crystallin A chain | 14 | 94795202 | rs9989237 | T | C | 0.21768 | 3301 | 0.0827 | 0.03 | 0.005888 | prot-a-673 |
| Cholesterol in small HDL | 14 | 94795202 | rs9989237 | T | C | 0.20942 | NA | -0.01388 | 0.005079 | 0.0059 | met-d-S_HDL_C |
| Cholesterol in very large HDL | 14 | 94795202 | rs9989237 | T | C | 0.20942 | NA | 0.011397 | 0.004594 | 0.0059 | met-d-XL_HDL_C |
| Broad bean intake | 14 | 94795202 | rs9989237 | T | C | 0.210565 | 64949 | -0.00759 | 0.002757 | 0.0059 | ukb-b-15212 |
| Main speciality of consultant (recoded) | 14 | 94795202 | rs9989237 | T | C | 0.7491 | NA | -0.3342 | 0.1214 | 0.00591 | ukb-e-41245_p48_MID |
| Memory B cell %lymphocyte | 14 | 94795202 | rs9989237 | T | C | 0.2674 | 3656 | -0.07851 | 0.02856 | 0.006017 | ebi-a-GCST90001436 |
| Powdered/instant soup intake | 14 | 94795202 | rs9989237 | T | C | 0.6217 | NA | 0.03567 | 0.01299 | 0.006025 | ukb-e-102530_CSA |
| Cochlin | 14 | 94795202 | rs9989237 | T | C | 0.21768 | 3301 | 0.0825 | 0.03 | 0.006026 | prot-a-613 |
| Free cholesterol in very large HDL | 14 | 94795202 | rs9989237 | T | C | 0.20942 | NA | 0.011607 | 0.004745 | 0.0061 | met-d-XL_HDL_FC |
| Other benign neoplasms of skin | 14 | 94795202 | rs9989237 | T | C | 0.2083 | NA | -0.0612 | 0.0223 | 0.00613 | finn-b-CD2_BENIGN_SKIN |
| TestASV_18 (Prevotella) abundance | 14 | 94795202 | rs9989237 | T | C | NA | 8956 | 0.172924 | 0.063125 | 0.006155 | ebi-a-GCST90011494 |
| Protein unc-13 homolog A | 14 | 94795202 | rs9989237 | T | C | 0.21768 | 3301 | 0.0823 | 0.03 | 0.006166 | prot-a-3165 |
| Inpatient record format | 14 | 94795202 | rs9989237 | T | C | 0.5929 | NA | -0.09098 | 0.03322 | 0.006173 | ukb-e-41253_p6_CSA |
| Amyotrophic lateral sclerosis (sporadic) | 14 | 94795202 | rs9989237 | T | C | NA | 4084 | 0.0106 | 0.0039 | 0.006216 | ebi-a-GCST004901 |
| Plateletcrit | 14 | 94795202 | rs9989237 | T | C | 0.2103 | 164339 | -0.01226 | 0.004481 | 0.006224 | ebi-a-GCST004607 |
| Sexual interference by partner or ex-partner without consent as an adult | 14 | 94795202 | rs9989237 | T | C | 0.7906 | NA | -0.09581 | 0.03505 | 0.006257 | ukb-e-20524_AFR |
| Cadherin-7 | 14 | 94795202 | rs9989237 | T | C | 0.21768 | 3301 | -0.082 | 0.03 | 0.00631 | prot-a-486 |
| Ephrin type-A receptor 10 | 14 | 94795202 | rs9989237 | T | C | 0.21768 | 3301 | -0.082 | 0.03 | 0.00631 | prot-a-955 |
| UDP-glucuronosyltransferase 1-6 | 14 | 94795202 | rs9989237 | T | C | 0.21768 | 3301 | -0.082 | 0.03 | 0.00631 | prot-a-3156 |
| BAG family molecular chaperone regulator 4 | 14 | 94795202 | rs9989237 | T | C | 0.21768 | 3301 | 0.0818 | 0.03 | 0.006457 | prot-a-226 |
| NET100 0015 | 14 | 94795202 | rs9989237 | T | C | 0.21004 | 7916 | 0.0528 | 0.0194 | 0.006457 | ubm-a-1171 |
| Umbilical hernia | 14 | 94795202 | rs9989237 | T | C | 0.2087 | NA | 0.0764 | 0.028 | 0.006457 | finn-b-K11_UMBHER |
| Paclitaxel drug response (AUC) | 14 | 94795202 | rs9989237 | T | C | 0.182 | 77 | 0.602112 | 0.215035 | 0.006494 | ebi-a-GCST90011791 |
| Triglycerides in small LDL | 14 | 94795202 | rs9989237 | T | C | 0.20942 | NA | -0.01296 | 0.004968 | 0.0066 | met-d-S_LDL_TG |
| Mono [ADP-ribose] polymerase PARP16 | 14 | 94795202 | rs9989237 | T | C | 0.21768 | 3301 | -0.0815 | 0.03 | 0.006607 | prot-a-2184 |
| Killer cell immunoglobulin-like receptor 2DL5A | 14 | 94795202 | rs9989237 | T | C | 0.21768 | 3301 | -0.0813 | 0.03 | 0.006761 | prot-a-1644 |
| lymphocyte cell count | 14 | 94795202 | rs9989237 | T | C | 0.210402 | 524920 | -0.00627 | 0.002316 | 0.006773 | ieu-b-32 |
| CD20 on IgD+ CD38- unswitched memory B cell | 14 | 94795202 | rs9989237 | T | C | 0.2688 | 1836 | 0.1051 | 0.03879 | 0.00679 | ebi-a-GCST90001750 |
| Phospholipids to total lipids ratio in large VLDL | 14 | 94795202 | rs9989237 | T | C | 0.209427 | NA | -0.01262 | 0.004943 | 0.0068 | met-d-L_VLDL_PL_pct |
| Phospholipids in medium LDL | 14 | 94795202 | rs9989237 | T | C | 0.20942 | NA | -0.01307 | 0.005096 | 0.0068 | met-d-M_LDL_PL |
| Synaptosomal-associated protein 23 levels | 14 | 94795202 | rs9989237 | T | C | 0.233 | 909 | 0.149924 | 0.054999 | 0.006814 | ebi-a-GCST90010347 |
| Medial epicondylitis | 14 | 94795202 | rs9989237 | T | C | 0.2076 | NA | 0.2065 | 0.0764 | 0.006847 | finn-b-M13_MEDIALEPICOND |
| Anti-varicella zoster virus IgG seropositivity | 14 | 94795202 | rs9989237 | T | C | 0.212501 | 8735 | 0.017869 | 0.006613 | 0.006888 | ebi-a-GCST90006928 |
| Treatment/medication code: nasonex 0.05% aqueous nasal spray | 14 | 94795202 | rs9989237 | T | C | 0.209974 | 462933 | -0.00049 | 0.00018 | 0.0069 | ukb-b-3337 |
| Cell surface glycoprotein CD200 receptor 2 | 14 | 94795202 | rs9989237 | T | C | 0.21768 | 3301 | -0.0811 | 0.03 | 0.006918 | prot-a-424 |
| Interferon-induced protein with tetratricopeptide repeats 2 | 14 | 94795202 | rs9989237 | T | C | 0.21768 | 3301 | -0.081 | 0.03 | 0.006918 | prot-a-1416 |
| NET100 0821 | 14 | 94795202 | rs9989237 | T | C | 0.21004 | 7916 | -0.0521 | 0.0193 | 0.006918 | ubm-a-1977 |
| OCT measured (left): Not performed - other reason | 14 | 94795202 | rs9989237 | T | C | 0.211448 | 54601 | 0.004022 | 0.001492 | 0.007027 | ukb-d-6072_7 |
| Ephrin-B3 | 14 | 94795202 | rs9989237 | T | C | 0.21768 | 3301 | 0.0809 | 0.03 | 0.007079 | prot-a-906 |
| Sodium-independent sulfate anion transporter | 14 | 94795202 | rs9989237 | T | C | 0.21768 | 3301 | 0.0808 | 0.03 | 0.007079 | prot-a-2750 |
| Titin | 14 | 94795202 | rs9989237 | T | C | 0.21768 | 3301 | 0.0809 | 0.03 | 0.007079 | prot-a-3116 |
| Age at last live birth | 14 | 94795202 | rs9989237 | T | C | 0.6166 | NA | 0.07112 | 0.02641 | 0.007086 | ukb-e-2764_CSA |
| Type of cancer: ICD10: D05.1 Intraductal carcinoma in situ | 14 | 94795202 | rs9989237 | T | C | 0.209969 | 463010 | -0.00044 | 0.000164 | 0.0071 | ukb-b-4674 |
| Job coding: public service higher/senior executive officer, hospital administrator | 14 | 94795202 | rs9989237 | T | C | 0.20828 | 89866 | 0.002898 | 0.001077 | 0.007129 | ukb-d-22601_35613203 |
| Total lipids in small HDL | 14 | 94795202 | rs9989237 | T | C | 0.20942 | NA | -0.01436 | 0.00507 | 0.0072 | met-d-S_HDL_L |
| Concentration of chylomicrons and extremely large VLDL particles | 14 | 94795202 | rs9989237 | T | C | 0.20942 | NA | -0.01249 | 0.004898 | 0.0072 | met-d-XXL_VLDL_P |
| Operation code: cholecystectomy/gall bladder removal | 14 | 94795202 | rs9989237 | T | C | 0.209974 | 462933 | -0.00133 | 0.000495 | 0.0072 | ukb-b-6235 |
| Vegetarian sausages/burgers intake | 14 | 94795202 | rs9989237 | T | C | 0.210565 | 64949 | 0.004855 | 0.001808 | 0.0072 | ukb-b-3383 |
| Inhibin beta C chain | 14 | 94795202 | rs9989237 | T | C | 0.21768 | 3301 | -0.0807 | 0.03 | 0.007244 | prot-a-1556 |
| Ferritin | 14 | 94795202 | rs9989237 | T | C | 0.21768 | 3301 | -0.0807 | 0.03 | 0.007244 | prot-a-1149 |
| DnaJ homolog subfamily B member 9 | 14 | 94795202 | rs9989237 | T | C | 0.21768 | 3301 | -0.0807 | 0.03 | 0.007244 | prot-a-842 |
| Sushi domain-containing protein 3 | 14 | 94795202 | rs9989237 | T | C | 0.21768 | 3301 | 0.0807 | 0.03 | 0.007244 | prot-a-2903 |
| Intrahepatic Cholestasis of Pregnancy (ICP)<c3><8a> | 14 | 94795202 | rs9989237 | T | C | 0.2083 | NA | -0.1568 | 0.0585 | 0.007349 | finn-b-O15_ICP |
| Fruitcake intake | 14 | 94795202 | rs9989237 | T | C | 0.7781 | NA | -0.05656 | 0.02112 | 0.007389 | ukb-e-102180_AFR |
| BET1-like protein | 14 | 94795202 | rs9989237 | T | C | 0.21768 | 3301 | 0.0804 | 0.03 | 0.007413 | prot-a-245 |
| Properdin | 14 | 94795202 | rs9989237 | T | C | 0.21768 | 3301 | -0.0805 | 0.03 | 0.007413 | prot-a-525 |
| Glypican-6 | 14 | 94795202 | rs9989237 | T | C | 0.21768 | 3301 | -0.0804 | 0.03 | 0.007413 | prot-a-1248 |
| NET100 1293 | 14 | 94795202 | rs9989237 | T | C | 0.21004 | 7916 | -0.0515 | 0.0193 | 0.007413 | ubm-a-2449 |
| NET100 0962 | 14 | 94795202 | rs9989237 | T | C | 0.21004 | 7916 | 0.052 | 0.0194 | 0.007413 | ubm-a-2118 |
| Average monthly spirits intake | 14 | 94795202 | rs9989237 | T | C | 0.211252 | 34111 | -0.01886 | 0.007051 | 0.0075 | ukb-b-10830 |
| Qualifications | 14 | 94795202 | rs9989237 | T | C | 0.5789 | NA | 0.252 | 0.09432 | 0.007536 | ukb-e-6138_p6_EAS |
| Job SOC coding: Chemists | 14 | 94795202 | rs9989237 | T | C | 0.208274 | 91149 | 0.001661 | 0.000622 | 0.00755 | ukb-d-22617_2111 |
| Protein jagged-2 | 14 | 94795202 | rs9989237 | T | C | 0.21768 | 3301 | 0.0801 | 0.03 | 0.007586 | prot-a-1597 |
| Galectin-7 | 14 | 94795202 | rs9989237 | T | C | 0.21768 | 3301 | 0.0803 | 0.03 | 0.007586 | prot-a-1729 |
| DKTatlas rh lateraloccipital area | 14 | 94795202 | rs9989237 | T | C | 0.21004 | 7916 | 0.0396 | 0.0149 | 0.007586 | ubm-a-2829 |
| a2009s lh G pariet inf-Supramar area | 14 | 94795202 | rs9989237 | T | C | 0.21004 | 7916 | 0.0401 | 0.015 | 0.007586 | ubm-a-2772 |
| P-selectin glycoprotein ligand 1 levels | 14 | 94795202 | rs9989237 | T | C | 0.236 | 1301 | 0.126689 | 0.047292 | 0.007587 | ebi-a-GCST90010165 |
| Pneumococcal septichemia | 14 | 94795202 | rs9989237 | T | C | 0.2083 | NA | 0.2209 | 0.0827 | 0.007605 | finn-b-PNEUMO_SEPTHICHE |
| Endometrial cancer (endometrioid histology) | 14 | 94795202 | rs9989237 | T | C | 0.216708 | 54884 | 0.05756 | 0.021609 | 0.007728 | ebi-a-GCST006465 |
| Mitochondrial ubiquitin ligase activator of NFKB 1 | 14 | 94795202 | rs9989237 | T | C | 0.21768 | 3301 | -0.0799 | 0.03 | 0.007762 | prot-a-1969 |
| Acidic leucine-rich nuclear phosphoprotein 32 family member A | 14 | 94795202 | rs9989237 | T | C | 0.21768 | 3301 | -0.08 | 0.03 | 0.007762 | prot-a-104 |
| Macrophage scavenger receptor types I and II | 14 | 94795202 | rs9989237 | T | C | 0.21768 | 3301 | -0.0798 | 0.03 | 0.007762 | prot-a-1951 |
| NET100 0977 | 14 | 94795202 | rs9989237 | T | C | 0.21004 | 7916 | -0.0512 | 0.0192 | 0.007762 | ubm-a-2133 |
| NET100 1128 | 14 | 94795202 | rs9989237 | T | C | 0.21004 | 7916 | 0.0511 | 0.0192 | 0.007762 | ubm-a-2284 |
| NET100 0669 | 14 | 94795202 | rs9989237 | T | C | 0.21004 | 7916 | -0.0515 | 0.0193 | 0.007762 | ubm-a-1825 |
| Intrahepatic Cholestasis of Pregnancy (ICP)<c3><8a> (more controls excluded) | 14 | 94795202 | rs9989237 | T | C | 0.2083 | NA | -0.1568 | 0.0589 | 0.007792 | finn-b-O15_ICP_EXMORE |
| Average diameter for HDL particles | 14 | 94795202 | rs9989237 | T | C | 0.20942 | NA | 0.010538 | 0.004513 | 0.0078 | met-d-HDL_size |
| Monocyte chemoattractant protein-3 levels | 14 | 94795202 | rs9989237 | T | C | NA | 843 | 0.1407 | 0.0529 | 0.007857 | ebi-a-GCST004437 |
| Milk added to cereal | 14 | 94795202 | rs9989237 | T | C | 0.6412 | NA | 0.209 | 0.0787 | 0.007913 | ukb-e-100890_CSA |
| Tumor necrosis factor ligand superfamily member 12 | 14 | 94795202 | rs9989237 | T | C | 0.21768 | 3301 | 0.0798 | 0.03 | 0.007943 | prot-a-3055 |
| Lysosomal acid phosphatase | 14 | 94795202 | rs9989237 | T | C | 0.21768 | 3301 | -0.0797 | 0.03 | 0.007943 | prot-a-19 |
| CD19 on Plasma Blast-Plasma Cell | 14 | 94795202 | rs9989237 | T | C | 0.2676 | 3657 | -0.07548 | 0.02844 | 0.007989 | ebi-a-GCST90001739 |
| Phosphoprotein associated with glycosphingolipid-enriched microdomains 1 levels | 14 | 94795202 | rs9989237 | T | C | 0.236 | 1308 | 0.122776 | 0.046108 | 0.008018 | ebi-a-GCST90010333 |
| mucin 16, cell surface associated | 14 | 94795202 | rs9989237 | T | C | 0.2384 | 3394 | 0.0832 | 0.0314 | 0.008083 | prot-b-75 |
| Zinc/RING finger protein 4 | 14 | 94795202 | rs9989237 | T | C | 0.21768 | 3301 | 0.0794 | 0.03 | 0.008128 | prot-a-3280 |
| Osteocalcin | 14 | 94795202 | rs9989237 | T | C | 0.21768 | 3301 | -0.0795 | 0.03 | 0.008128 | prot-a-246 |
| C-type lectin domain family 7 member A | 14 | 94795202 | rs9989237 | T | C | 0.21768 | 3301 | 0.0793 | 0.03 | 0.008318 | prot-a-581 |
| Uncharacterized protein C17orf89 | 14 | 94795202 | rs9989237 | T | C | 0.21768 | 3301 | 0.079 | 0.03 | 0.008511 | prot-a-289 |
| DKTatlas lh caudalanteriorcingulate area | 14 | 94795202 | rs9989237 | T | C | 0.21004 | 7916 | 0.0384 | 0.0146 | 0.008511 | ubm-a-2715 |
| CD25 on naive-mature B cell | 14 | 94795202 | rs9989237 | T | C | 0.2674 | 3657 | 0.07663 | 0.02911 | 0.008521 | ebi-a-GCST90001791 |
| IgD+ CD38- B cell %lymphocyte | 14 | 94795202 | rs9989237 | T | C | 0.2674 | 3656 | -0.07611 | 0.02892 | 0.008526 | ebi-a-GCST90001431 |
| CD28+ CD45RA- CD8+ T cell Absolute Count | 14 | 94795202 | rs9989237 | T | C | 0.2654 | 3408 | 0.07803 | 0.02969 | 0.008613 | ebi-a-GCST90001693 |
| Hematopoietic progenitor cell antigen CD34 | 14 | 94795202 | rs9989237 | T | C | 0.21768 | 3301 | 0.0788 | 0.03 | 0.00871 | prot-a-441 |
| Basigin | 14 | 94795202 | rs9989237 | T | C | 0.21768 | 3301 | 0.0789 | 0.03 | 0.00871 | prot-a-275 |
| Lipocalin-1 | 14 | 94795202 | rs9989237 | T | C | 0.21768 | 3301 | -0.0788 | 0.03 | 0.00871 | prot-a-1708 |
| G_Ruminococcaceae abundance | 14 | 94795202 | rs9989237 | T | C | NA | 8956 | -0.04935 | 0.018829 | 0.008764 | ebi-a-GCST90011343 |
| Free cholesterol to total lipids ratio in medium LDL | 14 | 94795202 | rs9989237 | T | C | 0.20942 | NA | 0.01229 | 0.00488 | 0.0088 | met-d-M_LDL_FC_pct |
| Gut microbiota abundance (genus Eisenbergiella id.11304) | 14 | 94795202 | rs9989237 | T | C | NA | 14306 | 0.059101 | 0.022842 | 0.008823 | ebi-a-GCST90016991 |
| Protein transport protein Sec61 subunit beta | 14 | 94795202 | rs9989237 | T | C | 0.21768 | 3301 | 0.0785 | 0.03 | 0.008913 | prot-a-2661 |
| Fructose-1,6-bisphosphatase 1 | 14 | 94795202 | rs9989237 | T | C | 0.21768 | 3301 | -0.0786 | 0.03 | 0.008913 | prot-a-1063 |
| Forkhead box protein L2 | 14 | 94795202 | rs9989237 | T | C | 0.21768 | 3301 | 0.0787 | 0.03 | 0.008913 | prot-a-1138 |
| a2009s rh G front middle area | 14 | 94795202 | rs9989237 | T | C | 0.21004 | 7916 | 0.0395 | 0.0151 | 0.008913 | ubm-a-2867 |
| NET100 1400 | 14 | 94795202 | rs9989237 | T | C | 0.21004 | 7916 | -0.0507 | 0.0194 | 0.008913 | ubm-a-2556 |
| Job SOC coding: Public service associate professionals | 14 | 94795202 | rs9989237 | T | C | 0.208274 | 91149 | 0.002805 | 0.001073 | 0.008933 | ukb-d-22617_3561 |
| Other retinal disorders | 14 | 94795202 | rs9989237 | T | C | 0.209437 | 361194 | -0.00052 | 0.000197 | 0.008994 | ukb-d-H7_RETINALDISOTH |
| Cholesterol in small LDL | 14 | 94795202 | rs9989237 | T | C | 0.20942 | NA | -0.01265 | 0.005094 | 0.009 | met-d-S_LDL_C |
| Relative age voice broke | 14 | 94795202 | rs9989237 | T | C | 0.607 | NA | 0.03786 | 0.0145 | 0.009018 | ukb-e-2385_CSA |
| Phospholipids to total lipids ratio in very small VLDL | 14 | 94795202 | rs9989237 | T | C | 0.20942 | NA | 0.01338 | 0.004966 | 0.0091 | met-d-XS_VLDL_PL_pct |
| Number of symbol digit matches made correctly | 14 | 94795202 | rs9989237 | T | C | 0.209624 | 113106 | 0.01336 | 0.005122 | 0.0091 | ukb-b-15625 |
| Nuclear receptor-binding protein | 14 | 94795202 | rs9989237 | T | C | 0.21768 | 3301 | -0.0784 | 0.03 | 0.00912 | prot-a-2093 |
| Serine palmitoyltransferase 1 | 14 | 94795202 | rs9989237 | T | C | 0.21768 | 3301 | 0.0784 | 0.03 | 0.00912 | prot-a-2836 |
| NET100 1226 | 14 | 94795202 | rs9989237 | T | C | 0.21004 | 7916 | 0.0502 | 0.0193 | 0.00912 | ubm-a-2382 |
| Non-cancer illness code, self-reported: cholelithiasis/gall stones | 14 | 94795202 | rs9989237 | T | C | 0.209974 | 462933 | -0.00085 | 0.000325 | 0.0092 | ukb-b-18700 |
| Stromal cell-derived factor 1 | 14 | 94795202 | rs9989237 | T | C | 0.21768 | 3301 | 0.0781 | 0.03 | 0.009333 | prot-a-740 |
| Other benign neoplasms of skin (all cancers excluded) | 14 | 94795202 | rs9989237 | T | C | 0.208 | NA | -0.0584 | 0.0225 | 0.009446 | finn-b-CD2_BENIGN_SKIN_EXALLC |
| Dickkopf-related protein 4 | 14 | 94795202 | rs9989237 | T | C | 0.21768 | 3301 | 0.0779 | 0.03 | 0.00955 | prot-a-823 |
| Melanoma-associated antigen 10 | 14 | 94795202 | rs9989237 | T | C | 0.21768 | 3301 | -0.0779 | 0.03 | 0.00955 | prot-a-1827 |
| Epidermal growth factor receptor substrate 15-like 1 | 14 | 94795202 | rs9989237 | T | C | 0.21768 | 3301 | 0.0778 | 0.03 | 0.00955 | prot-a-969 |
| Ribonuclease K6 | 14 | 94795202 | rs9989237 | T | C | 0.21768 | 3301 | -0.078 | 0.03 | 0.00955 | prot-a-2557 |
| a2009s lh G front middle area | 14 | 94795202 | rs9989237 | T | C | 0.21004 | 7916 | 0.0373 | 0.0144 | 0.00955 | ubm-a-2761 |
| NA | 14 | 94795202 | rs9989237 | T | C | 0.6112 | NA | 0.2401 | 0.09265 | 0.009563 | ukb-e-recode124_CSA |
| Surgery/amputation of toe or leg | 14 | 94795202 | rs9989237 | T | C | 0.91379 | NA | 0.8709 | 0.3362 | 0.009592 | ukb-e-5540_p1_AFR |
| Recurrent and persistent haematuria, drug-induced | 14 | 94795202 | rs9989237 | T | C | 0.2083 | NA | 0.3467 | 0.1339 | 0.009598 | finn-b-N14_RECURANDPERHEAMATUR_ADVERSE |
| Duodenal ulcer | 14 | 94795202 | rs9989237 | T | C | 0.2084 | NA | -0.112 | 0.0433 | 0.00965 | finn-b-K11_DULC |
| Psychiatric diseases | 14 | 94795202 | rs9989237 | T | C | 0.2083 | NA | 0.0258 | 0.01 | 0.009651 | finn-b-F5_PSYCH |
| 6mm index of best keratometry results (right) | 14 | 94795202 | rs9989237 | T | C | 0.7939 | NA | 0.0693 | 0.02678 | 0.009669 | ukb-e-5251_AFR |
| Ever had laser treatment for glaucoma or high eye pressure: No | 14 | 94795202 | rs9989237 | T | C | 0.212571 | 5788 | -0.01037 | 0.004006 | 0.009683 | ukb-d-5327_0 |
| Final attempt correct: no | 14 | 94795202 | rs9989237 | T | C | 0.210091 | 119729 | -0.00248 | 0.000958 | 0.00972 | ukb-d-4294_0 |
| Dolichyl-diphosphooligosaccharide--protein glycosyltransferase subunit 1 | 14 | 94795202 | rs9989237 | T | C | 0.21768 | 3301 | -0.0776 | 0.03 | 0.009772 | prot-a-2588 |
| Integral membrane protein DGCR2/IDD | 14 | 94795202 | rs9989237 | T | C | 0.21768 | 3301 | 0.0775 | 0.03 | 0.009772 | prot-a-809 |
| C-C motif chemokine 14 | 14 | 94795202 | rs9989237 | T | C | 0.21768 | 3301 | 0.0777 | 0.03 | 0.009772 | prot-a-390 |
| Retinol dehydrogenase 16 | 14 | 94795202 | rs9989237 | T | C | 0.21768 | 3301 | -0.0776 | 0.03 | 0.009772 | prot-a-2512 |
| Job coding: chemist (analytic, developmental, industrial, research) | 14 | 94795202 | rs9989237 | T | C | 0.20828 | 89866 | 0.001614 | 0.000625 | 0.009792 | ukb-d-22601_21113020 |
| Giant cell arteritis | 14 | 94795202 | rs9989237 | T | C | 0.2083 | NA | 0.2116 | 0.082 | 0.009845 | finn-b-M13_GIANTCELL |
| Diagnoses - main ICD10: H35 Other retinal disorders | 14 | 94795202 | rs9989237 | T | C | 0.209437 | 361194 | -0.0005 | 0.000193 | 0.00997 | ukb-d-H35 |
| Amyotrophic lateral sclerosis | 14 | 94795202 | rs9989237 | T | C | 0.216272 | 36052 | 0.010695 | 0.004152 | 0.009998 | ieu-a-1085 |
| Free cholesterol in large HDL | 14 | 94795202 | rs9989237 | T | C | 0.20942 | NA | 0.010156 | 0.004465 | 0.01 | met-d-L_HDL_FC |
| Cholesterol in chylomicrons and extremely large VLDL | 14 | 94795202 | rs9989237 | T | C | 0.20942 | NA | -0.01178 | 0.004908 | 0.01 | met-d-XXL_VLDL_C |
| Cholesteryl esters in very large VLDL | 14 | 94795202 | rs9989237 | T | C | 0.20942 | NA | -0.01221 | 0.00501 | 0.01 | met-d-XL_VLDL_CE |
| Mozzarella intake | 14 | 94795202 | rs9989237 | T | C | 0.210565 | 64949 | 0.003921 | 0.001531 | 0.01 | ukb-b-14505 |
| Riboflavin kinase | 14 | 94795202 | rs9989237 | T | C | 0.21768 | 3301 | -0.0774 | 0.03 | 0.01 | prot-a-2529 |
| Injuries to the shoulder and upper arm | 14 | 94795202 | rs9989237 | T | C | 0.2083 | NA | 0.0378 | 0.0147 | 0.0101 | finn-b-ST19_INJURI_SHOUL_UPPER_ARM |
| Creatinine | 14 | 94795202 | rs9989237 | T | C | 0.213281 | 24807 | 0.028818 | 0.011184 | 0.010112 | met-c-850 |
| Final attempt correct: yes | 14 | 94795202 | rs9989237 | T | C | 0.210091 | 119729 | 0.002495 | 0.000972 | 0.010226 | ukb-d-4294_1 |
| Mediator of RNA polymerase II transcription subunit 4 | 14 | 94795202 | rs9989237 | T | C | 0.21768 | 3301 | -0.0771 | 0.03 | 0.010233 | prot-a-1876 |
| Gamma-interferon-inducible protein 16 | 14 | 94795202 | rs9989237 | T | C | 0.21768 | 3301 | -0.0771 | 0.03 | 0.010233 | prot-a-1414 |
| Cadherin-1 | 14 | 94795202 | rs9989237 | T | C | 0.21768 | 3301 | -0.0772 | 0.03 | 0.010233 | prot-a-480 |
| Polypeptide N-acetylgalactosaminyltransferase 3 | 14 | 94795202 | rs9989237 | T | C | 0.21768 | 3301 | 0.0771 | 0.03 | 0.010233 | prot-a-1172 |
| Crohn's disease of large intestine | 14 | 94795202 | rs9989237 | T | C | 0.2081 | NA | -0.1607 | 0.0626 | 0.01028 | finn-b-CHRONLARGE |
| Collagen alpha-5(VI) chain | 14 | 94795202 | rs9989237 | T | C | 0.21768 | 3301 | -0.0768 | 0.03 | 0.010471 | prot-a-624 |
| Receptor-interacting serine/threonine-protein kinase 2 | 14 | 94795202 | rs9989237 | T | C | 0.21768 | 3301 | -0.0768 | 0.03 | 0.010471 | prot-a-2546 |
| Procalcitonin | 14 | 94795202 | rs9989237 | T | C | 0.21768 | 3301 | 0.077 | 0.03 | 0.010471 | prot-a-341 |
| Neudesin | 14 | 94795202 | rs9989237 | T | C | 0.21768 | 3301 | -0.0769 | 0.03 | 0.010471 | prot-a-2032 |
| Interleukin-10 receptor subunit beta | 14 | 94795202 | rs9989237 | T | C | 0.21768 | 3301 | 0.0768 | 0.03 | 0.010471 | prot-a-1465 |
| NET100 0256 | 14 | 94795202 | rs9989237 | T | C | 0.21004 | 7916 | 0.0493 | 0.0193 | 0.010471 | ubm-a-1412 |
| Charcot foot | 14 | 94795202 | rs9989237 | T | C | 0.2077 | NA | -0.3838 | 0.1502 | 0.0106 | finn-b-CHARCOT |
| Macular hole | 14 | 94795202 | rs9989237 | T | C | 0.2084 | NA | 0.1916 | 0.075 | 0.01065 | finn-b-MACULAR_HOLE |
| Facial pigmentation measurement (UV light) | 14 | 94795202 | rs9989237 | T | C | 0.2922 | 11079 | -0.3563 | 0.1396 | 0.01071 | ebi-a-GCST90002283 |
| Lymphocyte activation gene 3 protein | 14 | 94795202 | rs9989237 | T | C | 0.21768 | 3301 | -0.0767 | 0.03 | 0.010715 | prot-a-1692 |
| Arginine/serine-rich protein 1 | 14 | 94795202 | rs9989237 | T | C | 0.21768 | 3301 | 0.0767 | 0.03 | 0.010715 | prot-a-2607 |
| NET25 0180 | 14 | 94795202 | rs9989237 | T | C | 0.21004 | 7916 | 0.0485 | 0.019 | 0.010715 | ubm-a-1126 |
| Signal-to-noise-ratio (SNR) of triplet (left) | 14 | 94795202 | rs9989237 | T | C | 0.5954 | NA | -0.1058 | 0.04155 | 0.01084 | ukb-e-4230_EAS |
| Hot chocolate intake | 14 | 94795202 | rs9989237 | T | C | 0.7781 | NA | 0.07763 | 0.03049 | 0.0109 | ukb-e-100550_AFR |
| ADP-ribosylation factor-like protein 1 | 14 | 94795202 | rs9989237 | T | C | 0.21768 | 3301 | 0.0765 | 0.03 | 0.010965 | prot-a-161 |
| Fms-related tyrosine kinase 3 ligand | 14 | 94795202 | rs9989237 | T | C | 0.21768 | 3301 | -0.0764 | 0.03 | 0.010965 | prot-a-1128 |
| Ornithine decarboxylase | 14 | 94795202 | rs9989237 | T | C | 0.21768 | 3301 | -0.0764 | 0.03 | 0.010965 | prot-a-2142 |
| Synaptotagmin-17 | 14 | 94795202 | rs9989237 | T | C | 0.21768 | 3301 | 0.0764 | 0.03 | 0.010965 | prot-a-2912 |
| TCRgd T cell Absolute Count | 14 | 94795202 | rs9989237 | T | C | 0.2671 | 3650 | 0.07151 | 0.0281 | 0.01098 | ebi-a-GCST90001615 |
| Triglycerides in LDL | 14 | 94795202 | rs9989237 | T | C | 0.20942 | NA | -0.01231 | 0.005034 | 0.011 | met-d-LDL_TG |
| Triglycerides in very small VLDL | 14 | 94795202 | rs9989237 | T | C | 0.20942 | NA | -0.01208 | 0.005013 | 0.011 | met-d-XS_VLDL_TG |
| Variation in diet | 14 | 94795202 | rs9989237 | T | C | 0.209946 | 460884 | -0.00385 | 0.001514 | 0.011 | ukb-b-2909 |
| Operative procedures - main OPCS: Q35.2 Endoscopic bilateral clipping of fallopian tubes | 14 | 94795202 | rs9989237 | T | C | 0.209969 | 463010 | 0.000458 | 0.000181 | 0.011 | ukb-b-16269 |
| Sources of admission to hospital (recoded): Other admission source | 14 | 94795202 | rs9989237 | T | C | 0.209925 | 461365 | -0.00044 | 0.000174 | 0.011 | ukb-b-13922 |
| OTU97_24 (Prevotella) abundance | 14 | 94795202 | rs9989237 | T | C | NA | 8956 | 0.138943 | 0.054655 | 0.011017 | ebi-a-GCST90011385 |
| 3mm asymmetry angle (right) | 14 | 94795202 | rs9989237 | T | C | 0.6107 | NA | -0.0608 | 0.02394 | 0.01109 | ukb-e-5108_CSA |
| white blood cell count | 14 | 94795202 | rs9989237 | T | C | 0.210559 | 562240 | -0.00572 | 0.002253 | 0.011112 | ieu-b-30 |
| Shoulder region | 14 | 94795202 | rs9989237 | T | C | 0.2083 | NA | -0.3951 | 0.1556 | 0.01112 | finn-b-Z21_SHOUL_REGION |
| Killer cell immunoglobulin-like receptor 2DL4 | 14 | 94795202 | rs9989237 | T | C | 0.21768 | 3301 | 0.0762 | 0.03 | 0.01122 | prot-a-1641 |
| bipolar disorder | 14 | 94795202 | rs9989237 | T | C | 0.208639 | 51710 | -0.0418 | 0.0165 | 0.01125 | ieu-b-41 |
| Aspartate aminotransferase | 14 | 94795202 | rs9989237 | T | C | 0.20968 | 342990 | -0.00711 | 0.002807 | 0.011357 | ukb-d-30650_irnt |
| Other or ill-defined heart diseases (no controls excluded) | 14 | 94795202 | rs9989237 | T | C | 0.2083 | NA | 0.1654 | 0.0654 | 0.01139 | finn-b-I9_OTHILLHEART_EXNONE |
| DNA-binding protein inhibitor ID-2 | 14 | 94795202 | rs9989237 | T | C | 0.21768 | 3301 | 0.076 | 0.03 | 0.011482 | prot-a-1408 |
| NET100 0832 | 14 | 94795202 | rs9989237 | T | C | 0.21004 | 7916 | -0.0489 | 0.0193 | 0.011482 | ubm-a-1988 |
| kallikrein-11 levels | 14 | 94795202 | rs9989237 | T | C | 0.2248 | 21758 | 0.0432 | 0.0171 | 0.01164 | ebi-a-GCST90012012 |
| Total bilirubin | 14 | 94795202 | rs9989237 | T | C | 0.20971 | 342829 | -0.00712 | 0.002822 | 0.011659 | ukb-d-30840_irnt |
| UPF0488 protein C8orf33 | 14 | 94795202 | rs9989237 | T | C | 0.21768 | 3301 | 0.0757 | 0.03 | 0.011749 | prot-a-323 |
| Apolipoprotein M | 14 | 94795202 | rs9989237 | T | C | 0.21768 | 3301 | -0.0757 | 0.03 | 0.011749 | prot-a-137 |
| E3 ubiquitin-protein ligase RNF43 | 14 | 94795202 | rs9989237 | T | C | 0.21768 | 3301 | -0.0758 | 0.03 | 0.011749 | prot-a-2572 |
| a2009s lh G temp sup-Lateral area | 14 | 94795202 | rs9989237 | T | C | 0.21004 | 7916 | 0.0367 | 0.0146 | 0.011749 | ubm-a-2780 |
| Free cholesterol to total lipids ratio in very large HDL | 14 | 94795202 | rs9989237 | T | C | 0.209409 | NA | -0.00969 | 0.004514 | 0.012 | met-d-XL_HDL_FC_pct |
| Cholesteryl esters in VLDL | 14 | 94795202 | rs9989237 | T | C | 0.20942 | NA | -0.01248 | 0.00509 | 0.012 | met-d-VLDL_CE |
| Operation code: upper limb surgery | 14 | 94795202 | rs9989237 | T | C | 0.209974 | 462933 | -0.00048 | 0.000191 | 0.012 | ukb-b-290 |
| PCT where patients GP was registered: SOUTH LEEDS PCT | 14 | 94795202 | rs9989237 | T | C | 0.209969 | 463010 | -0.0004 | 0.000161 | 0.012 | ukb-b-20178 |
| Hands-free device/speakerphone use with mobile phone in last 3 month | 14 | 94795202 | rs9989237 | T | C | 0.209624 | 388531 | -0.00587 | 0.002325 | 0.012 | ukb-b-20432 |
| neutrophil cell count | 14 | 94795202 | rs9989237 | T | C | 0.210243 | 519285 | -0.00592 | 0.002357 | 0.012016 | ieu-b-34 |
| Fibronectin type III domain-containing protein 5 | 14 | 94795202 | rs9989237 | T | C | 0.21768 | 3301 | -0.0754 | 0.03 | 0.012023 | prot-a-1133 |
| Membrane protein FAM159B | 14 | 94795202 | rs9989237 | T | C | 0.21768 | 3301 | 0.0754 | 0.03 | 0.012023 | prot-a-1026 |
| CD70 antigen | 14 | 94795202 | rs9989237 | T | C | 0.21768 | 3301 | 0.0754 | 0.03 | 0.012023 | prot-a-457 |
| Any mental disorder | 14 | 94795202 | rs9989237 | T | C | 0.2083 | NA | 0.0255 | 0.0102 | 0.01208 | finn-b-KRA_PSY_ANYMENTAL |
| Suicide or other Intentional self-harm | 14 | 94795202 | rs9989237 | T | C | 0.2083 | NA | 0.0255 | 0.0102 | 0.01208 | finn-b-VWXY20_SUICI_OTHER_INTENTI_SELF_H |
| Pain in joint | 14 | 94795202 | rs9989237 | T | C | 0.2085 | NA | 0.0422 | 0.0169 | 0.01225 | finn-b-JOINTPAIN |
| NKG2-E type II integral membrane protein | 14 | 94795202 | rs9989237 | T | C | 0.21768 | 3301 | -0.0751 | 0.03 | 0.012303 | prot-a-1670 |
| Polypeptide N-acetylgalactosaminyltransferase 11 | 14 | 94795202 | rs9989237 | T | C | 0.21768 | 3301 | 0.0752 | 0.03 | 0.012303 | prot-a-1168 |
| Protein NDRG4 | 14 | 94795202 | rs9989237 | T | C | 0.21768 | 3301 | -0.0753 | 0.03 | 0.012303 | prot-a-2020 |
| NET25 0033 | 14 | 94795202 | rs9989237 | T | C | 0.21004 | 7916 | 0.0476 | 0.019 | 0.012303 | ubm-a-979 |
| IDP dMRI TBSS L1 Fornix | 14 | 94795202 | rs9989237 | T | C | 0.21004 | 7916 | -0.0409 | 0.0163 | 0.012303 | ubm-a-345 |
| Platelet count | 14 | 94795202 | rs9989237 | T | C | 0.387939 | 145648 | -0.00955 | 0.003815 | 0.012346 | ebi-a-GCST90002358 |
| Abnormal heart sounds | 14 | 94795202 | rs9989237 | T | C | 0.7943 | NA | 0.3073 | 0.1229 | 0.01241 | ukb-e-396_AFR |
| C-type lectin domain family 2 member L | 14 | 94795202 | rs9989237 | T | C | 0.21768 | 3301 | 0.0749 | 0.03 | 0.012589 | prot-a-575 |
| NADH dehydrogenase [ubiquinone] iron-sulfur protein 4, mitochondrial | 14 | 94795202 | rs9989237 | T | C | 0.21768 | 3301 | -0.075 | 0.03 | 0.012589 | prot-a-2025 |
| a2009s lh G orbital area | 14 | 94795202 | rs9989237 | T | C | 0.21004 | 7916 | 0.0343 | 0.0137 | 0.012589 | ubm-a-2770 |
| DKTatlas lh lateralorbitofrontal area | 14 | 94795202 | rs9989237 | T | C | 0.21004 | 7916 | 0.0321 | 0.0129 | 0.012589 | ubm-a-2724 |
| DKTatlas lh rostralmiddlefrontal area | 14 | 94795202 | rs9989237 | T | C | 0.21004 | 7916 | 0.0342 | 0.0137 | 0.012589 | ubm-a-2739 |
| Serine/threonine-protein phosphatase 2A regulatory subunit B'' subunit alpha | 14 | 94795202 | rs9989237 | T | C | 0.21768 | 3301 | -0.0746 | 0.03 | 0.012883 | prot-a-2349 |
| Myc proto-oncogene protein | 14 | 94795202 | rs9989237 | T | C | 0.21768 | 3301 | 0.0747 | 0.03 | 0.012883 | prot-a-1978 |
| NET100 0987 | 14 | 94795202 | rs9989237 | T | C | 0.21004 | 7916 | 0.0479 | 0.0192 | 0.012883 | ubm-a-2143 |
| Diagnoses - main ICD10: L02 Cutaneous abscess, furuncle and carbuncle | 14 | 94795202 | rs9989237 | T | C | 0.209437 | 361194 | 0.000506 | 0.000203 | 0.012884 | ukb-d-L02 |
| Pollinosis | 14 | 94795202 | rs9989237 | T | C | 0.386834 | NA | 0.050065 | 0.020137 | 0.012909 | bbj-a-146 |
| Cholesterol in large HDL | 14 | 94795202 | rs9989237 | T | C | 0.20942 | NA | 0.009854 | 0.004487 | 0.013 | met-d-L_HDL_C |
| Carbohydrate sulfotransferase 6 | 14 | 94795202 | rs9989237 | T | C | 0.21768 | 3301 | 0.0746 | 0.03 | 0.013183 | prot-a-558 |
| Beta-defensin 118 | 14 | 94795202 | rs9989237 | T | C | 0.21768 | 3301 | 0.0744 | 0.03 | 0.013183 | prot-a-792 |
| Amyotrophic lateral sclerosis | 14 | 94795202 | rs9989237 | T | C | NA | 36052 | 0.0528 | 0.0213 | 0.01329 | ebi-a-GCST004692 |
| Female genital prolapse | 14 | 94795202 | rs9989237 | T | C | 0.2075 | NA | 0.0539 | 0.0218 | 0.01331 | finn-b-N14_FEMGENPROL |
| Ulcerative colitis (strict definition, all Crohn cases excluded) | 14 | 94795202 | rs9989237 | T | C | 0.2084 | NA | 0.0938 | 0.0379 | 0.01331 | finn-b-K11_UC_NOCD |
| hl.rand_norm(mean=pop_dict[mt.pop], seed=42) | 14 | 94795202 | rs9989237 | T | C | 0.5758 | NA | -0.06971 | 0.02817 | 0.01334 | ukb-e-recode8_EAS |
| hl.rand_norm(seed=42) | 14 | 94795202 | rs9989237 | T | C | 0.5758 | NA | -0.06972 | 0.02817 | 0.01334 | ukb-e-recode7_EAS |
| SLE, strict definition | 14 | 94795202 | rs9989237 | T | C | 0.2083 | NA | -0.1924 | 0.0778 | 0.01341 | finn-b-SLE_STRICT |
| NA | 14 | 94795202 | rs9989237 | T | C | 0.6115 | NA | 0.1818 | 0.07356 | 0.01343 | ukb-e-recode24_CSA |
| Complement C1q tumor necrosis factor-related protein 3 | 14 | 94795202 | rs9989237 | T | C | 0.21768 | 3301 | -0.0742 | 0.03 | 0.01349 | prot-a-304 |
| Ephrin-B1 | 14 | 94795202 | rs9989237 | T | C | 0.21768 | 3301 | -0.0743 | 0.03 | 0.01349 | prot-a-903 |
| Phenylalanine--tRNA ligase, mitochondrial | 14 | 94795202 | rs9989237 | T | C | 0.21768 | 3301 | -0.0743 | 0.03 | 0.01349 | prot-a-1055 |
| Tissue Factor | 14 | 94795202 | rs9989237 | T | C | 0.21768 | 3301 | -0.0743 | 0.03 | 0.01349 | prot-a-1008 |
| DKTatlas lh caudalmiddlefrontal area | 14 | 94795202 | rs9989237 | T | C | 0.21004 | 7916 | 0.0383 | 0.0155 | 0.01349 | ubm-a-2716 |
| Mycoses | 14 | 94795202 | rs9989237 | T | C | 0.2083 | NA | 0.0692 | 0.028 | 0.01354 | finn-b-AB1_MYCOSES |
| OTU99_24 (Prevotella) abundance | 14 | 94795202 | rs9989237 | T | C | NA | 8956 | 0.13831 | 0.056054 | 0.013609 | ebi-a-GCST90011447 |
| Tropomyosin alpha-3 chain | 14 | 94795202 | rs9989237 | T | C | 0.21768 | 3301 | 0.0739 | 0.03 | 0.013804 | prot-a-3087 |
| NACHT, LRR and PYD domains-containing protein 4 | 14 | 94795202 | rs9989237 | T | C | 0.21768 | 3301 | 0.0739 | 0.03 | 0.013804 | prot-a-2057 |
| Centrosomal protein of 57 kDa | 14 | 94795202 | rs9989237 | T | C | 0.21768 | 3301 | 0.0741 | 0.03 | 0.013804 | prot-a-514 |
| Dual specificity mitogen-activated protein kinase kinase 3 | 14 | 94795202 | rs9989237 | T | C | 0.21768 | 3301 | -0.074 | 0.03 | 0.013804 | prot-a-1841 |
| Noninflammatory disorders of female genital tract | 14 | 94795202 | rs9989237 | T | C | 0.2083 | NA | 0.0268 | 0.0109 | 0.01396 | finn-b-N14_FEMALEGENNONINF |
| Cholesteryl esters in large HDL | 14 | 94795202 | rs9989237 | T | C | 0.20942 | NA | 0.009741 | 0.0045 | 0.014 | met-d-L_HDL_CE |
| Intake of artificial sweetener added to coffee | 14 | 94795202 | rs9989237 | T | C | 0.210565 | 64949 | 0.010186 | 0.004146 | 0.014 | ukb-b-1338 |
| Emphysema | 14 | 94795202 | rs9989237 | T | C | 0.2082 | NA | -0.1676 | 0.0682 | 0.01402 | finn-b-J10_EMPHYSEMA |
| Day-of-week questionnaire completion requested: Wednesday | 14 | 94795202 | rs9989237 | T | C | 0.209871 | 51427 | 0.007013 | 0.002855 | 0.014022 | ukb-d-20079_3 |
| Pterin-4-alpha-carbinolamine dehydratase | 14 | 94795202 | rs9989237 | T | C | 0.21768 | 3301 | -0.0737 | 0.03 | 0.014125 | prot-a-2191 |
| Discoidin, CUB and LCCL domain-containing protein 2 | 14 | 94795202 | rs9989237 | T | C | 0.21768 | 3301 | -0.0738 | 0.03 | 0.014125 | prot-a-766 |
| Tumor necrosis factor ligand superfamily member 15 | 14 | 94795202 | rs9989237 | T | C | 0.21768 | 3301 | -0.0736 | 0.03 | 0.014125 | prot-a-3058 |
| Serine/threonine-protein kinase ULK3 | 14 | 94795202 | rs9989237 | T | C | 0.21768 | 3301 | 0.0737 | 0.03 | 0.014125 | prot-a-3163 |
| Other or ill-defined heart diseases | 14 | 94795202 | rs9989237 | T | C | 0.2086 | NA | 0.1603 | 0.0655 | 0.01437 | finn-b-I9_OTHILLHEART |
| Protein NDNF | 14 | 94795202 | rs9989237 | T | C | 0.21768 | 3301 | 0.0734 | 0.03 | 0.014454 | prot-a-2019 |
| NET100 1270 | 14 | 94795202 | rs9989237 | T | C | 0.21004 | 7916 | -0.0473 | 0.0193 | 0.014454 | ubm-a-2426 |
| Time spent watching television (TV) | 14 | 94795202 | rs9989237 | T | C | 0.6107 | NA | 0.03091 | 0.01264 | 0.01448 | ukb-e-1070_CSA |
| Acute myeloid leukaemia (all cancers excluded) | 14 | 94795202 | rs9989237 | T | C | 0.2081 | NA | 0.4484 | 0.1835 | 0.01455 | finn-b-C3_AML_EXALLC |
| Gut microbiota abundance (family Rikenellaceae id.967) | 14 | 94795202 | rs9989237 | T | C | NA | 14306 | -0.03164 | 0.012847 | 0.014597 | ebi-a-GCST90016950 |
| Breakfast cereal consumed | 14 | 94795202 | rs9989237 | T | C | 0.6414 | NA | 0.1915 | 0.07842 | 0.01462 | ukb-e-100760_CSA |
| "Carcinoma in situ of skin, other sites/unspecified" (all cancers excluded) | 14 | 94795202 | rs9989237 | T | C | 0.2081 | NA | 0.4284 | 0.1755 | 0.01463 | finn-b-CD2_INSITU_SKIN_NOS_EXALLC |
| Vitamin and mineral supplements | 14 | 94795202 | rs9989237 | T | C | 0.6078 | NA | -0.2133 | 0.08739 | 0.01463 | ukb-e-6155_p6_CSA |
| Chocolate sweet intake | 14 | 94795202 | rs9989237 | T | C | 0.6217 | NA | 0.04328 | 0.01774 | 0.0147 | ukb-e-102310_CSA |
| Fasting insulin | 14 | 94795202 | rs9989237 | T | C | 0.195 | 8353 | 0.0229 | 0.0116 | 0.01472 | ebi-a-GCST90002240 |
| IgD+ CD24+ B cell %lymphocyte | 14 | 94795202 | rs9989237 | T | C | 0.2674 | 3656 | -0.07012 | 0.02874 | 0.01475 | ebi-a-GCST90001439 |
| Number of operations, self-reported | 14 | 94795202 | rs9989237 | T | C | 0.793 | NA | 0.04031 | 0.01653 | 0.01477 | ukb-e-136_p1_AFR |
| Alcohol dehydrogenase 4 | 14 | 94795202 | rs9989237 | T | C | 0.21768 | 3301 | -0.0732 | 0.03 | 0.014791 | prot-a-47 |
| Low affinity immunoglobulin gamma Fc region receptor III-B | 14 | 94795202 | rs9989237 | T | C | 0.21768 | 3301 | 0.0732 | 0.03 | 0.014791 | prot-a-1076 |
| a2009s lh G parietal sup area | 14 | 94795202 | rs9989237 | T | C | 0.21004 | 7916 | 0.0398 | 0.0163 | 0.014791 | ubm-a-2773 |
| DKTatlas rh parsopercularis area | 14 | 94795202 | rs9989237 | T | C | 0.21004 | 7916 | 0.0392 | 0.0161 | 0.014791 | ubm-a-2836 |
| a2009s lh Pole temporal thickness | 14 | 94795202 | rs9989237 | T | C | 0.21004 | 7916 | -0.0441 | 0.0181 | 0.014791 | ubm-a-3001 |
| Reason for glasses/contact lenses | 14 | 94795202 | rs9989237 | T | C | 0.5684 | NA | -0.1926 | 0.07905 | 0.01484 | ukb-e-6147_p1_EAS |
| Total lipids in small LDL | 14 | 94795202 | rs9989237 | T | C | 0.20942 | NA | -0.01178 | 0.005088 | 0.015 | met-d-S_LDL_L |
| Number of operations, self-reported | 14 | 94795202 | rs9989237 | T | C | 0.209974 | 462933 | -0.00486 | 0.00199 | 0.015 | ukb-b-4733 |
| Operation code: rectal or colon polypectomy | 14 | 94795202 | rs9989237 | T | C | 0.209974 | 462933 | -0.0004 | 0.000164 | 0.015 | ukb-b-19438 |
| Arm fat-free mass (left) | 14 | 94795202 | rs9989237 | T | C | 0.209989 | 454672 | -0.00377 | 0.001548 | 0.015 | ukb-b-19925 |
| Diagnoses - main ICD10: D25.9 Leiomyoma of uterus, unspecified | 14 | 94795202 | rs9989237 | T | C | 0.209969 | 463010 | 0.000665 | 0.000272 | 0.015 | ukb-b-9536 |
| Diagnoses - secondary ICD10: K30 Dyspepsia | 14 | 94795202 | rs9989237 | T | C | 0.209969 | 463010 | -0.00041 | 0.000168 | 0.015 | ukb-b-12556 |
| Extrapyramidal and movement disorders | 14 | 94795202 | rs9989237 | T | C | 0.2083 | NA | 0.0624 | 0.0257 | 0.01509 | finn-b-G6_XTRAPYR |
| Vesicle-associated membrane protein-associated protein B/C | 14 | 94795202 | rs9989237 | T | C | 0.21768 | 3301 | 0.073 | 0.03 | 0.015136 | prot-a-3190 |
| C-type lectin domain family 4 member K | 14 | 94795202 | rs9989237 | T | C | 0.21768 | 3301 | 0.0731 | 0.03 | 0.015136 | prot-a-425 |
| Phosphatidylinositol transfer protein alpha isoform | 14 | 94795202 | rs9989237 | T | C | 0.21768 | 3301 | -0.0731 | 0.03 | 0.015136 | prot-a-2278 |
| NET100 0892 | 14 | 94795202 | rs9989237 | T | C | 0.21004 | 7916 | 0.0472 | 0.0194 | 0.015136 | ubm-a-2048 |
| a2009s rh S temporal sup area | 14 | 94795202 | rs9989237 | T | C | 0.21004 | 7916 | 0.0355 | 0.0146 | 0.015136 | ubm-a-2925 |
| ECG, phase duration | 14 | 94795202 | rs9989237 | T | C | 0.8098 | NA | 0.2062 | 0.08499 | 0.01528 | ukb-e-5992_AFR |
| Tinnitus | 14 | 94795202 | rs9989237 | T | C | 0.6102 | NA | -0.05442 | 0.02244 | 0.0153 | ukb-e-4803_CSA |
| Benign neoplasm: Skin of other and unspecified parts of face | 14 | 94795202 | rs9989237 | T | C | 0.2083 | NA | -0.1039 | 0.0428 | 0.01531 | finn-b-CD2_BENIGN_SKIN_FACE |
| OTU97_150 (Bacteroides) abundance | 14 | 94795202 | rs9989237 | T | C | NA | 8956 | -0.23518 | 0.097038 | 0.015369 | ebi-a-GCST90011374 |
| Acute myeloid leukaemia | 14 | 94795202 | rs9989237 | T | C | 0.2083 | NA | 0.443 | 0.183 | 0.01547 | finn-b-C3_AML |
| ADP-ribose pyrophosphatase, mitochondrial | 14 | 94795202 | rs9989237 | T | C | 0.21768 | 3301 | -0.0728 | 0.03 | 0.015488 | prot-a-2129 |
| Sialic acid-binding Ig-like lectin 14 | 14 | 94795202 | rs9989237 | T | C | 0.21768 | 3301 | 0.0728 | 0.03 | 0.015488 | prot-a-2727 |
| Heat shock 70 kDa protein 6 | 14 | 94795202 | rs9989237 | T | C | 0.21768 | 3301 | -0.0726 | 0.03 | 0.015488 | prot-a-1388 |
| C-C motif chemokine 24 | 14 | 94795202 | rs9989237 | T | C | 0.21768 | 3301 | 0.0728 | 0.03 | 0.015488 | prot-a-401 |
| Tumor necrosis factor receptor superfamily member 4 | 14 | 94795202 | rs9989237 | T | C | 0.21768 | 3301 | 0.0727 | 0.03 | 0.015488 | prot-a-3051 |
| IDP dMRI TBSS ISOVF Fornix | 14 | 94795202 | rs9989237 | T | C | 0.21004 | 7916 | -0.0393 | 0.0162 | 0.015488 | ubm-a-585 |
| IDP T1 SIENAX grey unnormalised volume | 14 | 94795202 | rs9989237 | T | C | 0.21004 | 7916 | 0.021 | 0.0087 | 0.015488 | ubm-a-6 |
| Radiation-related disorders of the skin and subcutaneous tissue | 14 | 94795202 | rs9989237 | T | C | 0.2083 | NA | -0.0606 | 0.025 | 0.01551 | finn-b-L12_RADIATIONRELATEDSKIN |
| Haematocrit percentage | 14 | 94795202 | rs9989237 | T | C | 0.209415 | 350475 | 0.005773 | 0.002387 | 0.015591 | ukb-d-30030_irnt |
| Job SOC coding: Business and related associate professionals n.e.c. | 14 | 94795202 | rs9989237 | T | C | 0.208274 | 91149 | -0.00203 | 0.000839 | 0.015638 | ukb-d-22617_3539 |
| Carcinoma in situ of skin, other sites/unspecified | 14 | 94795202 | rs9989237 | T | C | 0.2083 | NA | 0.4248 | 0.1758 | 0.01567 | finn-b-CD2_INSITU_SKIN_NOS |
| Gastroduodenal ulcer | 14 | 94795202 | rs9989237 | T | C | 0.2083 | NA | -0.0655 | 0.0271 | 0.0157 | finn-b-K11_GASTRODUOULC |
| Other and unspecified disorders of pigmentation | 14 | 94795202 | rs9989237 | T | C | 0.2082 | NA | 0.4475 | 0.1853 | 0.01572 | finn-b-L12_PIGMENTNAS |
| Melanoma in situ of other and unspecified parts of face | 14 | 94795202 | rs9989237 | T | C | 0.2083 | NA | -0.3912 | 0.1621 | 0.01582 | finn-b-CD2_INSITU_MELANOMA_FACE |
| Collagen alpha-1(XX) chain | 14 | 94795202 | rs9989237 | T | C | 0.21768 | 3301 | 0.0725 | 0.03 | 0.015849 | prot-a-619 |
| CD70 antigen | 14 | 94795202 | rs9989237 | T | C | 0.21768 | 3301 | 0.0725 | 0.03 | 0.015849 | prot-a-458 |
| Alpha-N-acetylgalactosaminide alpha-2,6-sialyltransferase 3 | 14 | 94795202 | rs9989237 | T | C | 0.21768 | 3301 | 0.0726 | 0.03 | 0.015849 | prot-a-2855 |
| Tumor necrosis factor receptor superfamily member 11A | 14 | 94795202 | rs9989237 | T | C | 0.21768 | 3301 | -0.0724 | 0.03 | 0.015849 | prot-a-3036 |
| Protein enabled homolog | 14 | 94795202 | rs9989237 | T | C | 0.21768 | 3301 | 0.0724 | 0.03 | 0.015849 | prot-a-941 |
| Fetal and adult testis-expressed transcript protein | 14 | 94795202 | rs9989237 | T | C | 0.21768 | 3301 | 0.0725 | 0.03 | 0.015849 | prot-a-1060 |
| GRB2-related adapter protein 2 | 14 | 94795202 | rs9989237 | T | C | 0.21768 | 3301 | 0.0724 | 0.03 | 0.015849 | prot-a-1269 |
| a2009s lh S precentral-inf-part area | 14 | 94795202 | rs9989237 | T | C | 0.21004 | 7916 | 0.0409 | 0.0169 | 0.015849 | ubm-a-2814 |
| IDP T1 SIENAX grey normalised volume | 14 | 94795202 | rs9989237 | T | C | 0.21004 | 7916 | 0.0306 | 0.0127 | 0.015849 | ubm-a-5 |
| Diagnoses - main ICD10: K25 Gastric ulcer | 14 | 94795202 | rs9989237 | T | C | 0.209437 | 361194 | -0.00052 | 0.000215 | 0.015898 | ukb-d-K25 |
| Index of best refractometry result (right) | 14 | 94795202 | rs9989237 | T | C | 0.5928 | NA | 0.1056 | 0.04379 | 0.0159 | ukb-e-5221_EAS |
| Ohter specific/unspecified arthritis | 14 | 94795202 | rs9989237 | T | C | 0.2083 | NA | 0.0664 | 0.0275 | 0.01591 | finn-b-M13_ARTHRITISNAS |
| HLA DR+ Natural Killer %Natural Killer | 14 | 94795202 | rs9989237 | T | C | 0.2679 | 3596 | -0.07046 | 0.02921 | 0.01592 | ebi-a-GCST90001649 |
| Chronic hepatitis, not elsewhere classified | 14 | 94795202 | rs9989237 | T | C | 0.2082 | NA | 0.1985 | 0.0824 | 0.01596 | finn-b-K11_CHRONHEP |
| Cholesteryl esters to total lipids ratio in very large VLDL | 14 | 94795202 | rs9989237 | T | C | 0.209295 | NA | 0.011385 | 0.004884 | 0.016 | met-d-XL_VLDL_CE_pct |
| Cholesteryl esters in LDL | 14 | 94795202 | rs9989237 | T | C | 0.20942 | NA | -0.01157 | 0.005075 | 0.016 | met-d-LDL_CE |
| Couscous intake | 14 | 94795202 | rs9989237 | T | C | 0.210565 | 64949 | 0.004126 | 0.001707 | 0.016 | ukb-b-758 |
| Maximum digits remembered correctly | 14 | 94795202 | rs9989237 | T | C | 0.6168 | NA | 0.08355 | 0.0347 | 0.01605 | ukb-e-4282_CSA |
| Heparin-binding EGF-like growth factor | 14 | 94795202 | rs9989237 | T | C | 0.21768 | 3301 | 0.0722 | 0.03 | 0.016218 | prot-a-1313 |
| DKTatlas lh precentral area | 14 | 94795202 | rs9989237 | T | C | 0.21004 | 7916 | 0.0329 | 0.0137 | 0.016218 | ubm-a-2736 |
| Macrophage Migration Inhibitory Factor levels | 14 | 94795202 | rs9989237 | T | C | NA | 3494 | -0.0714 | 0.0295 | 0.01624 | ebi-a-GCST004423 |
| Intrahepatic Cholestasis of Pregnancy (ICP)<c3><8a>, incl. ICD-8 | 14 | 94795202 | rs9989237 | T | C | 0.2083 | NA | -0.1227 | 0.0511 | 0.01625 | finn-b-O15_ICP_WIDE |
| Type 2 diabetes with neurological complications | 14 | 94795202 | rs9989237 | T | C | 0.2077 | NA | -0.1435 | 0.0597 | 0.01633 | finn-b-E4_DM2NEU |
| Dipeptidyl peptidase 2 levels | 14 | 94795202 | rs9989237 | T | C | 0.234 | 1290 | 0.113205 | 0.046962 | 0.016359 | ebi-a-GCST90010307 |
| Job coding: management information officer, conference/events co-ordinator/organiser, exhibition officer, work study engineer/officer/analyst, contract adviser/agent, election agent, business system analyst | 14 | 94795202 | rs9989237 | T | C | 0.20828 | 89866 | -0.00198 | 0.000825 | 0.016362 | ukb-d-22601_35393271 |
| Anxiety disorders (more controls excluded) | 14 | 94795202 | rs9989237 | T | C | 0.2078 | NA | 0.0356 | 0.0149 | 0.01647 | finn-b-KRA_PSY_ANXIETY_EXMORE |
| Candidiasis | 14 | 94795202 | rs9989237 | T | C | 0.2082 | NA | 0.0944 | 0.0394 | 0.01649 | finn-b-AB1_CANDIDIASIS |
| Gro-beta/gamma | 14 | 94795202 | rs9989237 | T | C | 0.21768 | 3301 | 0.0719 | 0.03 | 0.016596 | prot-a-746 |
| Matrix-remodeling-associated protein 8 | 14 | 94795202 | rs9989237 | T | C | 0.21768 | 3301 | 0.072 | 0.03 | 0.016596 | prot-a-1975 |
| Bone morphogenetic protein 7 | 14 | 94795202 | rs9989237 | T | C | 0.21768 | 3301 | 0.0719 | 0.03 | 0.016596 | prot-a-257 |
| Latent-transforming growth factor beta-binding protein 4 | 14 | 94795202 | rs9989237 | T | C | 0.21768 | 3301 | 0.0719 | 0.03 | 0.016596 | prot-a-1806 |
| Membrane protein FAM174B | 14 | 94795202 | rs9989237 | T | C | 0.21768 | 3301 | 0.072 | 0.03 | 0.016596 | prot-a-1035 |
| NET100 0831 | 14 | 94795202 | rs9989237 | T | C | 0.21004 | 7916 | 0.046 | 0.0192 | 0.016596 | ubm-a-1987 |
| Handedness (chirality/laterality) | 14 | 94795202 | rs9989237 | T | C | 0.6112 | NA | 0.1587 | 0.0664 | 0.01686 | ukb-e-1707_p2_CSA |
| ETS-related transcription factor Elf-5 | 14 | 94795202 | rs9989237 | T | C | 0.21768 | 3301 | 0.0716 | 0.03 | 0.016982 | prot-a-929 |
| Killer cell immunoglobulin-like receptor 3DS1 | 14 | 94795202 | rs9989237 | T | C | 0.21768 | 3301 | 0.0718 | 0.03 | 0.016982 | prot-a-1648 |
| Protein disulfide-isomerase A3 | 14 | 94795202 | rs9989237 | T | C | 0.21768 | 3301 | 0.0716 | 0.03 | 0.016982 | prot-a-2232 |
| Dickkopf-related protein 1 | 14 | 94795202 | rs9989237 | T | C | 0.21768 | 3301 | 0.0717 | 0.03 | 0.016982 | prot-a-821 |
| IQ domain-containing protein F1 | 14 | 94795202 | rs9989237 | T | C | 0.21768 | 3301 | -0.0716 | 0.03 | 0.016982 | prot-a-1565 |
| Lysosomal protective protein | 14 | 94795202 | rs9989237 | T | C | 0.21768 | 3301 | 0.0717 | 0.03 | 0.016982 | prot-a-717 |
| Contactin-associated protein 1 | 14 | 94795202 | rs9989237 | T | C | 0.21768 | 3301 | 0.0717 | 0.03 | 0.016982 | prot-a-610 |
| Galectin-1 | 14 | 94795202 | rs9989237 | T | C | 0.21768 | 3301 | -0.0717 | 0.03 | 0.016982 | prot-a-1726 |
| NET25 0054 | 14 | 94795202 | rs9989237 | T | C | 0.21004 | 7916 | 0.0458 | 0.0192 | 0.016982 | ubm-a-1000 |
| a2009s rh G&S occipital inf area | 14 | 94795202 | rs9989237 | T | C | 0.21004 | 7916 | 0.0388 | 0.0162 | 0.016982 | ubm-a-2854 |
| NET100 0368 | 14 | 94795202 | rs9989237 | T | C | 0.21004 | 7916 | 0.0459 | 0.0192 | 0.016982 | ubm-a-1524 |
| NET100 1272 | 14 | 94795202 | rs9989237 | T | C | 0.21004 | 7916 | -0.0461 | 0.0193 | 0.016982 | ubm-a-2428 |
| Operation code: anal surgery | 14 | 94795202 | rs9989237 | T | C | 0.209974 | 462933 | 0.000475 | 0.0002 | 0.017 | ukb-b-17277 |
| Interval between previous point and current one in numeric path (trail #1) | 14 | 94795202 | rs9989237 | T | C | 0.209914 | 109986 | -0.01241 | 0.005206 | 0.017 | ukb-b-9917 |
| Emotional lability | 14 | 94795202 | rs9989237 | T | C | 0.2271 | 3268 | -0.0685 | 0.0287 | 0.01706 | ebi-a-GCST90013452 |
| Need for immunization against other single viral diseases | 14 | 94795202 | rs9989237 | T | C | 0.2082 | NA | -0.24 | 0.1007 | 0.01713 | finn-b-Z21_NEED_IMMUNI_OTH_SINGLE_VIRAL_DISEA |
| Glomerulonephritis | 14 | 94795202 | rs9989237 | T | C | 0.2083 | NA | -0.0637 | 0.0267 | 0.01715 | finn-b-GLOMER_NEPHRITIS |
| Protein | 14 | 94795202 | rs9989237 | T | C | 0.6217 | NA | 0.111 | 0.04658 | 0.01718 | ukb-e-100003_CSA |
| CD24+ CD27+ B cell %lymphocyte | 14 | 94795202 | rs9989237 | T | C | 0.2674 | 3656 | -0.0678 | 0.02848 | 0.01733 | ebi-a-GCST90001442 |
| Hepatocyte growth factor-regulated tyrosine kinase substrate | 14 | 94795202 | rs9989237 | T | C | 0.21768 | 3301 | 0.0714 | 0.03 | 0.017378 | prot-a-1335 |
| Rap1 GTPase-activating protein 1 | 14 | 94795202 | rs9989237 | T | C | 0.21768 | 3301 | -0.0715 | 0.03 | 0.017378 | prot-a-2489 |
| Heparan sulfate glucosamine 3-O-sulfotransferase 3B1 | 14 | 94795202 | rs9989237 | T | C | 0.21768 | 3301 | -0.0715 | 0.03 | 0.017378 | prot-a-1376 |
| NET100 1348 | 14 | 94795202 | rs9989237 | T | C | 0.21004 | 7916 | -0.0459 | 0.0193 | 0.017378 | ubm-a-2504 |
| NODEamps100 0051 | 14 | 94795202 | rs9989237 | T | C | 0.21004 | 7916 | 0.0386 | 0.0162 | 0.017378 | ubm-a-942 |
| Any mental disorder, or suicide (or attempt), or psychic disorders complicating pregnancy, partum or puerperum or nerve system disorders | 14 | 94795202 | rs9989237 | T | C | 0.2083 | NA | 0.0241 | 0.0101 | 0.0175 | finn-b-KRA_PSY_ANYMENTAL_SUICID_PREG_NERV |
| Other and specified injuries of hip and thigh | 14 | 94795202 | rs9989237 | T | C | 0.2084 | NA | -0.2749 | 0.1157 | 0.01751 | finn-b-ST19_OTHER_SPECIFE_INJURI_HIP_THIGH1 |
| CD8dim Natural Killer T Absolute Count | 14 | 94795202 | rs9989237 | T | C | 0.2671 | 3652 | 0.06741 | 0.02836 | 0.01752 | ebi-a-GCST90001633 |
| Sum basophil neutrophil counts | 14 | 94795202 | rs9989237 | T | C | 0.2106 | 171529 | 0.010284 | 0.004333 | 0.01762 | ebi-a-GCST004621 |
| Depression medications | 14 | 94795202 | rs9989237 | T | C | 0.2081 | NA | 0.0295 | 0.0124 | 0.01774 | finn-b-ANTIDEPRESSANTS |
| Hepcidin | 14 | 94795202 | rs9989237 | T | C | 0.21768 | 3301 | -0.0711 | 0.03 | 0.017783 | prot-a-1307 |
| Interleukin-24 | 14 | 94795202 | rs9989237 | T | C | 0.21768 | 3301 | 0.0711 | 0.03 | 0.017783 | prot-a-1514 |
| Protein Z-dependent protease inhibitor | 14 | 94795202 | rs9989237 | T | C | 0.21768 | 3301 | -0.0712 | 0.03 | 0.017783 | prot-a-2688 |
| a2009s lh G&S paracentral thickness | 14 | 94795202 | rs9989237 | T | C | 0.21004 | 7916 | -0.0426 | 0.018 | 0.017783 | ubm-a-2961 |
| NET100 0788 | 14 | 94795202 | rs9989237 | T | C | 0.21004 | 7916 | -0.0452 | 0.0191 | 0.017783 | ubm-a-1944 |
| Triglycerides in large LDL | 14 | 94795202 | rs9989237 | T | C | 0.20942 | NA | -0.01143 | 0.005043 | 0.018 | met-d-L_LDL_TG |
| Signal-to-noise-ratio (SNR) of triplet (left) | 14 | 94795202 | rs9989237 | T | C | 0.210592 | 148728 | -0.01049 | 0.004438 | 0.018 | ukb-b-43 |
| Morning/evening person (chronotype) | 14 | 94795202 | rs9989237 | T | C | 0.5777 | NA | 0.06936 | 0.02936 | 0.01818 | ukb-e-1180_EAS |
| Tumor necrosis factor receptor superfamily member 10A | 14 | 94795202 | rs9989237 | T | C | 0.21768 | 3301 | 0.0709 | 0.03 | 0.018197 | prot-a-3032 |
| Ribosome-binding protein 1 | 14 | 94795202 | rs9989237 | T | C | 0.21768 | 3301 | 0.0711 | 0.03 | 0.018197 | prot-a-2600 |
| Neuron-specific protein family member 1 | 14 | 94795202 | rs9989237 | T | C | 0.21768 | 3301 | 0.071 | 0.03 | 0.018197 | prot-a-2110 |
| Zinc finger protein 18 | 14 | 94795202 | rs9989237 | T | C | 0.21768 | 3301 | -0.0709 | 0.03 | 0.018197 | prot-a-3264 |
| C-C motif chemokine 17 | 14 | 94795202 | rs9989237 | T | C | 0.21768 | 3301 | 0.071 | 0.03 | 0.018197 | prot-a-394 |
| Probable ATP-dependent RNA helicase DHX58 | 14 | 94795202 | rs9989237 | T | C | 0.21768 | 3301 | -0.0709 | 0.03 | 0.018197 | prot-a-815 |
| NET100 1237 | 14 | 94795202 | rs9989237 | T | C | 0.21004 | 7916 | -0.0455 | 0.0192 | 0.018197 | ubm-a-2393 |
| DKTatlas lh supramarginal area | 14 | 94795202 | rs9989237 | T | C | 0.21004 | 7916 | 0.034 | 0.0144 | 0.018197 | ubm-a-2743 |
| IDP T1 FAST ROIs R supracalc cortex | 14 | 94795202 | rs9989237 | T | C | 0.21004 | 7916 | 0.0391 | 0.0166 | 0.018197 | ubm-a-119 |
| DKTatlas lh superiortemporal area | 14 | 94795202 | rs9989237 | T | C | 0.21004 | 7916 | 0.03 | 0.0127 | 0.018197 | ubm-a-2742 |
| NET100 0599 | 14 | 94795202 | rs9989237 | T | C | 0.21004 | 7916 | -0.0457 | 0.0194 | 0.018197 | ubm-a-1755 |
| Length of working week for main job | 14 | 94795202 | rs9989237 | T | C | 0.7917 | NA | 0.07212 | 0.03059 | 0.0184 | ukb-e-767_AFR |
| Iron | 14 | 94795202 | rs9989237 | T | C | 0.6217 | NA | 0.105 | 0.04454 | 0.01844 | ukb-e-100011_CSA |
| Melanoma in situ of other and unspecified parts of face (all cancers excluded) | 14 | 94795202 | rs9989237 | T | C | 0.2081 | NA | -0.3825 | 0.1623 | 0.01847 | finn-b-CD2_INSITU_MELANOMA_FACE_EXALLC |
| Ras-related protein Rap-2a | 14 | 94795202 | rs9989237 | T | C | 0.21768 | 3301 | 0.0708 | 0.03 | 0.018621 | prot-a-2491 |
| Carbonic anhydrase 9 | 14 | 94795202 | rs9989237 | T | C | 0.21768 | 3301 | 0.0707 | 0.03 | 0.018621 | prot-a-334 |
| CUGBP Elav-like family member 2 | 14 | 94795202 | rs9989237 | T | C | 0.21768 | 3301 | 0.0707 | 0.03 | 0.018621 | prot-a-510 |
| Zinc finger protein SNAI2 | 14 | 94795202 | rs9989237 | T | C | 0.21768 | 3301 | -0.0707 | 0.03 | 0.018621 | prot-a-2785 |
| Growth/differentiation factor 8 | 14 | 94795202 | rs9989237 | T | C | 0.21768 | 3301 | -0.0707 | 0.03 | 0.018621 | prot-a-1956 |
| Ephrin type-B receptor 2 | 14 | 94795202 | rs9989237 | T | C | 0.21768 | 3301 | -0.0706 | 0.03 | 0.018621 | prot-a-960 |
| a2009s rh S temporal inf area | 14 | 94795202 | rs9989237 | T | C | 0.21004 | 7916 | 0.0372 | 0.0158 | 0.018621 | ubm-a-2924 |
| IDP tfMRI 90th-percentile BOLD faces | 14 | 94795202 | rs9989237 | T | C | 0.21004 | 7916 | 0.0448 | 0.0191 | 0.018621 | ubm-a-185 |
| DKTatlas lh pericalcarine thickness | 14 | 94795202 | rs9989237 | T | C | 0.21004 | 7916 | -0.0421 | 0.0179 | 0.018621 | ubm-a-2945 |
| Congenital malformations of the musculoskeletal system, not elsewhere classified | 14 | 94795202 | rs9989237 | T | C | 0.2083 | NA | 0.3125 | 0.1328 | 0.01865 | finn-b-Q17_CONGEN_MALFO_MUSCULOS_SYSTEM_NOT_ELSEW_CLASSIFIED |
| Calcific tendinitis of shoulder | 14 | 94795202 | rs9989237 | T | C | 0.2076 | NA | 0.1443 | 0.0614 | 0.01866 | finn-b-M13_CALCIFICTEND |
| Leg pain when walking normally | 14 | 94795202 | rs9989237 | T | C | 0.7641 | NA | -0.236 | 0.1004 | 0.0187 | ukb-e-5485_AFR |
| Crohn disease (strict definition, require KELA, min 2 HDR) | 14 | 94795202 | rs9989237 | T | C | 0.2083 | NA | -0.1439 | 0.0612 | 0.01873 | finn-b-K11_CD_STRICT2 |
| Soft tissue disorders related to use, overuse and pressure | 14 | 94795202 | rs9989237 | T | C | 0.209437 | 361194 | -0.00038 | 0.000162 | 0.018791 | ukb-d-M13_SOFTOVERUSE |
| Malignant neoplasm of ovary | 14 | 94795202 | rs9989237 | T | C | 0.2083 | NA | -0.1543 | 0.0657 | 0.01894 | finn-b-C3_OVARY |
| Triglycerides to total lipids ratio in medium HDL | 14 | 94795202 | rs9989237 | T | C | 0.20942 | NA | -0.0112 | 0.004885 | 0.019 | met-d-M_HDL_TG_pct |
| Concentration of very large HDL particles | 14 | 94795202 | rs9989237 | T | C | 0.20942 | NA | 0.009184 | 0.004584 | 0.019 | met-d-XL_HDL_P |
| Sex hormone binding globulin (SHBG) | 14 | 94795202 | rs9989237 | T | C | 0.210262 | NA | -0.00851 | 0.003626 | 0.019 | ieu-b-4870 |
| Sushi intake | 14 | 94795202 | rs9989237 | T | C | 0.210565 | 64949 | 0.002045 | 0.000872 | 0.019 | ukb-b-5213 |
| Fractured bone site(s): Other bones | 14 | 94795202 | rs9989237 | T | C | 0.209964 | 460340 | -0.00134 | 0.00057 | 0.019 | ukb-b-17738 |
| Current employment status: Unable to work because of sickness or disability | 14 | 94795202 | rs9989237 | T | C | 0.209385 | 336252 | -0.00133 | 0.000567 | 0.019069 | ukb-a-407 |
| Age first had sexual intercourse | 14 | 94795202 | rs9989237 | T | C | 0.6082 | NA | 0.04913 | 0.02096 | 0.01909 | ukb-e-2139_CSA |
| C-C motif chemokine 24 levels | 14 | 94795202 | rs9989237 | T | C | 0.234 | 1323 | 0.109035 | 0.046431 | 0.019121 | ebi-a-GCST90010197 |
| Benign neoplasm: Skin of other and unspecified parts of face (all cancers excluded) | 14 | 94795202 | rs9989237 | T | C | 0.208 | NA | -0.1006 | 0.043 | 0.01913 | finn-b-CD2_BENIGN_SKIN_FACE_EXALLC |
| Sudden idiopathic hearing loss | 14 | 94795202 | rs9989237 | T | C | 0.2083 | NA | 0.1076 | 0.0459 | 0.01918 | finn-b-H8_HL_IDIOP |
| Time spent watching television (TV) | 14 | 94795202 | rs9989237 | T | C | 0.7937 | NA | -0.04026 | 0.01722 | 0.01936 | ukb-e-1070_AFR |
| Female infertility, tubal origin | 14 | 94795202 | rs9989237 | T | C | 0.208 | NA | -0.149 | 0.0638 | 0.01948 | finn-b-N14_FITUB |
| Gap junction alpha-1 protein | 14 | 94795202 | rs9989237 | T | C | 0.21768 | 3301 | 0.0703 | 0.03 | 0.019498 | prot-a-1213 |
| NKG2-E type II integral membrane protein | 14 | 94795202 | rs9989237 | T | C | 0.21768 | 3301 | -0.0701 | 0.0301 | 0.019498 | prot-a-1671 |
| NET100 1191 | 14 | 94795202 | rs9989237 | T | C | 0.21004 | 7916 | 0.0452 | 0.0194 | 0.019498 | ubm-a-2347 |
| NET100 0348 | 14 | 94795202 | rs9989237 | T | C | 0.21004 | 7916 | -0.0439 | 0.0188 | 0.019498 | ubm-a-1504 |
| SLE (Finngen) | 14 | 94795202 | rs9989237 | T | C | 0.2084 | NA | -0.1772 | 0.0759 | 0.01951 | finn-b-SLE_FG |
| Spinal stenosis | 14 | 94795202 | rs9989237 | T | C | 0.208 | NA | 0.0478 | 0.0205 | 0.01952 | finn-b-M13_SPINSTENOSIS |
| Type 1 diabetes with renal complications | 14 | 94795202 | rs9989237 | T | C | 0.2077 | NA | -0.1356 | 0.0581 | 0.01958 | finn-b-E4_DM1REN |
| Age at first birth | 14 | 94795202 | rs9989237 | T | C | NA | 124088 | 0.0602 | 0.0258 | 0.01975 | ebi-a-GCST90000049 |
| Kallikrein-10 levels | 14 | 94795202 | rs9989237 | T | C | 0.235 | 1312 | 0.109572 | 0.046907 | 0.019832 | ebi-a-GCST90010320 |
| Diagnoses - main ICD10: M70 Soft tissue disorders related to use, overuse and pressure | 14 | 94795202 | rs9989237 | T | C | 0.209437 | 361194 | -0.00038 | 0.000162 | 0.019877 | ukb-d-M70 |
| Carboxypeptidase B levels | 14 | 94795202 | rs9989237 | T | C | 0.234 | 1323 | 0.108228 | 0.046316 | 0.019899 | ebi-a-GCST90010206 |
| Disintegrin and metalloproteinase domain-containing protein 29 | 14 | 94795202 | rs9989237 | T | C | 0.21768 | 3301 | 0.0699 | 0.03 | 0.019953 | prot-a-29 |
| Galactoside 3(4)-L-fucosyltransferase | 14 | 94795202 | rs9989237 | T | C | 0.21768 | 3301 | -0.07 | 0.03 | 0.019953 | prot-a-1152 |
| Surfeit locus protein 1 | 14 | 94795202 | rs9989237 | T | C | 0.21768 | 3301 | -0.07 | 0.03 | 0.019953 | prot-a-2900 |
| Endoplasmic reticulum mannosyl-oligosaccharide 1,2-alpha-mannosidase | 14 | 94795202 | rs9989237 | T | C | 0.21768 | 3301 | -0.07 | 0.03 | 0.019953 | prot-a-1834 |
| Protein deglycase DJ-1 | 14 | 94795202 | rs9989237 | T | C | 0.21768 | 3301 | -0.0699 | 0.03 | 0.019953 | prot-a-2181 |
| NET100 0068 | 14 | 94795202 | rs9989237 | T | C | 0.21004 | 7916 | 0.0451 | 0.0193 | 0.019953 | ubm-a-1224 |
| NET100 0085 | 14 | 94795202 | rs9989237 | T | C | 0.21004 | 7916 | -0.0451 | 0.0194 | 0.019953 | ubm-a-1241 |
| NET100 0888 | 14 | 94795202 | rs9989237 | T | C | 0.21004 | 7916 | 0.0448 | 0.0193 | 0.019953 | ubm-a-2044 |
| a2009s rh G precentral area | 14 | 94795202 | rs9989237 | T | C | 0.21004 | 7916 | 0.0352 | 0.0152 | 0.019953 | ubm-a-2881 |
| Milk type used | 14 | 94795202 | rs9989237 | T | C | 0.7902 | NA | -0.161 | 0.06921 | 0.01999 | ukb-e-1418_p3_AFR |
| Triglycerides in HDL | 14 | 94795202 | rs9989237 | T | C | 0.20942 | NA | -0.01232 | 0.00502 | 0.02 | met-d-HDL_TG |
| Total lipids in LDL | 14 | 94795202 | rs9989237 | T | C | 0.20942 | NA | -0.01119 | 0.005072 | 0.02 | met-d-LDL_L |
| Triglycerides in medium HDL | 14 | 94795202 | rs9989237 | T | C | 0.20942 | NA | -0.01216 | 0.005028 | 0.02 | met-d-M_HDL_TG |
| Bioavailable testosterone levels | 14 | 94795202 | rs9989237 | T | C | 0.210023 | 178782 | 0.00738 | 0.003743 | 0.02 | ebi-a-GCST90012103 |
| Hearing aid user | 14 | 94795202 | rs9989237 | T | C | 0.210357 | 280546 | -0.00167 | 0.000714 | 0.02 | ukb-b-19060 |
| Fasting time | 14 | 94795202 | rs9989237 | T | C | 0.209965 | 462992 | 0.004866 | 0.002089 | 0.02 | ukb-b-16156 |
| Non-cancer illness code, self-reported: gestational hypertension/pre-eclampsia | 14 | 94795202 | rs9989237 | T | C | 0.209974 | 462933 | -0.00037 | 0.000161 | 0.02 | ukb-b-13535 |
| R00 Abnormalities of heart beat | 14 | 94795202 | rs9989237 | T | C | 0.7944 | NA | 0.2954 | 0.1271 | 0.02008 | ukb-e-R00_AFR |
| NA | 14 | 94795202 | rs9989237 | T | C | 0.791 | NA | -0.2198 | 0.09458 | 0.0201 | ukb-e-recode238_AFR |
| UC patients in KELA-register ( KELA 208 prior to 1994, or ICD K51) | 14 | 94795202 | rs9989237 | T | C | 0.2083 | NA | 0.0772 | 0.0333 | 0.0203 | finn-b-K11_KELAUC |
| Treatment speciality of consultant (recoded) | 14 | 94795202 | rs9989237 | T | C | 0.7678 | NA | 0.3217 | 0.1386 | 0.0203 | ukb-e-41246_p33_MID |
| CD45 on CD33+ HLA DR+ CD14- | 14 | 94795202 | rs9989237 | T | C | 0.2704 | 1579 | 0.1003 | 0.0432 | 0.02031 | ebi-a-GCST90002042 |
| Tumor necrosis factor receptor 1 levels | 14 | 94795202 | rs9989237 | T | C | 0.2198 | 21758 | 0.0258 | 0.0111 | 0.02034 | ebi-a-GCST90012015 |
| Lymphocyte function-associated antigen 3 | 14 | 94795202 | rs9989237 | T | C | 0.21768 | 3301 | -0.0697 | 0.03 | 0.020417 | prot-a-454 |
| Carbonic anhydrase 2 | 14 | 94795202 | rs9989237 | T | C | 0.21768 | 3301 | 0.0697 | 0.03 | 0.020417 | prot-a-329 |
| SLIT and NTRK-like protein 1 | 14 | 94795202 | rs9989237 | T | C | 0.21768 | 3301 | 0.0696 | 0.0301 | 0.020417 | prot-a-2766 |
| EH domain-containing protein 4 | 14 | 94795202 | rs9989237 | T | C | 0.21768 | 3301 | 0.0698 | 0.03 | 0.020417 | prot-a-912 |
| Fibroblast growth factor receptor 2 | 14 | 94795202 | rs9989237 | T | C | 0.21768 | 3301 | 0.0697 | 0.03 | 0.020417 | prot-a-1102 |
| Kallikrein-4 | 14 | 94795202 | rs9989237 | T | C | 0.21768 | 3301 | 0.0696 | 0.03 | 0.020417 | prot-a-1662 |
| volume CSF | 14 | 94795202 | rs9989237 | T | C | 0.21004 | 7916 | -0.0366 | 0.0158 | 0.020417 | ubm-a-2669 |
| Chronic kidney disease | 14 | 94795202 | rs9989237 | T | C | 0.2082 | NA | -0.0674 | 0.0291 | 0.02048 | finn-b-N14_CHRONKIDNEYDIS |
| Arginase-1 levels | 14 | 94795202 | rs9989237 | T | C | 0.232 | 1072 | 0.117051 | 0.050342 | 0.020665 | ebi-a-GCST90010286 |
| Hospital episode type: General episode | 14 | 94795202 | rs9989237 | T | C | 0.209437 | 361194 | -0.00298 | 0.001289 | 0.020789 | ukb-d-41231_1 |
| Temporal arteritis | 14 | 94795202 | rs9989237 | T | C | 0.2083 | NA | 0.1972 | 0.0853 | 0.02085 | finn-b-M13_TEMPARTERIT |
| Disorders of lipoid metabolism | 14 | 94795202 | rs9989237 | T | C | 0.7513 | NA | -0.2881 | 0.1247 | 0.02087 | ukb-e-272_MID |
| Nidogen-1 | 14 | 94795202 | rs9989237 | T | C | 0.21768 | 3301 | 0.0694 | 0.03 | 0.020893 | prot-a-2049 |
| NET100 0597 | 14 | 94795202 | rs9989237 | T | C | 0.21004 | 7916 | 0.0449 | 0.0194 | 0.020893 | ubm-a-1753 |
| Drive faster than motorway speed limit | 14 | 94795202 | rs9989237 | T | C | 0.6066 | NA | -0.02754 | 0.01193 | 0.02096 | ukb-e-1100_p1_CSA |
| Puerperal sepsis | 14 | 94795202 | rs9989237 | T | C | 0.2085 | NA | 0.086 | 0.0372 | 0.02098 | finn-b-O15_PUERP_SEPSIS |
| Phospholipids in LDL | 14 | 94795202 | rs9989237 | T | C | 0.20942 | NA | -0.01094 | 0.005077 | 0.021 | met-d-LDL_PL |
| Phospholipids to total lipids ratio in small HDL | 14 | 94795202 | rs9989237 | T | C | 0.20942 | NA | 0.008975 | 0.004919 | 0.021 | met-d-S_HDL_PL_pct |
| Bipolar disorder vs major depressive disorder (ordinary least squares (OLS)) | 14 | 94795202 | rs9989237 | T | C | NA | 191108 | -0.0064 | 0.0027 | 0.021 | ebi-a-GCST90016607 |
| Systemic lupus erythematosus | 14 | 94795202 | rs9989237 | T | C | 0.2082 | NA | -0.1745 | 0.0757 | 0.02115 | finn-b-M13_SLE |
| Benign neoplasm: Pancreas (all cancers excluded) | 14 | 94795202 | rs9989237 | T | C | 0.2081 | NA | 0.3145 | 0.1365 | 0.0212 | finn-b-CD2_BENIGN_PANCREAS_EXALLC |
| Constipation | 14 | 94795202 | rs9989237 | T | C | 0.7902 | NA | -0.2445 | 0.1062 | 0.02135 | ukb-e-563_AFR |
| Derlin-1 | 14 | 94795202 | rs9989237 | T | C | 0.21768 | 3301 | -0.069 | 0.0301 | 0.02138 | prot-a-806 |
| Programmed cell death protein 1 | 14 | 94795202 | rs9989237 | T | C | 0.21768 | 3301 | 0.0693 | 0.03 | 0.02138 | prot-a-2214 |
| MAX-interacting protein 1 | 14 | 94795202 | rs9989237 | T | C | 0.21768 | 3301 | 0.0693 | 0.03 | 0.02138 | prot-a-1973 |
| Relaxin receptor 1 | 14 | 94795202 | rs9989237 | T | C | 0.21768 | 3301 | 0.0691 | 0.0301 | 0.02138 | prot-a-2612 |
| Trem-like transcript 1 protein | 14 | 94795202 | rs9989237 | T | C | 0.21768 | 3301 | 0.0691 | 0.0301 | 0.02138 | prot-a-3099 |
| GDNF family receptor alpha-3 | 14 | 94795202 | rs9989237 | T | C | 0.21768 | 3301 | 0.0692 | 0.0301 | 0.02138 | prot-a-1203 |
| Protein FAM163B | 14 | 94795202 | rs9989237 | T | C | 0.21768 | 3301 | 0.0692 | 0.0301 | 0.02138 | prot-a-1028 |
| NODEamps100 0016 | 14 | 94795202 | rs9989237 | T | C | 0.21004 | 7916 | 0.0437 | 0.019 | 0.02138 | ubm-a-907 |
| volume MaskVol-to-eTIV | 14 | 94795202 | rs9989237 | T | C | 0.21004 | 7916 | 0.0409 | 0.0178 | 0.02138 | ubm-a-2710 |
| NET100 1134 | 14 | 94795202 | rs9989237 | T | C | 0.21004 | 7916 | 0.0443 | 0.0192 | 0.02138 | ubm-a-2290 |
| Triglycerides | 14 | 94795202 | rs9989237 | T | C | 0.20967 | 343992 | -0.00655 | 0.002847 | 0.021427 | ukb-d-30870_irnt |
| Osteoarthrosis | 14 | 94795202 | rs9989237 | T | C | 0.6119 | NA | 0.15 | 0.06523 | 0.02144 | ukb-e-740_CSA |
| Giant cell arteritis with polymyalgia rheumatica | 14 | 94795202 | rs9989237 | T | C | 0.2083 | NA | 0.1966 | 0.0855 | 0.0215 | finn-b-GIANT_CELL_TEMP_ARTERITIS |
| Month of birth | 14 | 94795202 | rs9989237 | T | C | 0.6075 | NA | -0.13 | 0.05663 | 0.02169 | ukb-e-52_p10_CSA |
| Lesion of femoral nerve | 14 | 94795202 | rs9989237 | T | C | 0.2081 | NA | -0.3981 | 0.1734 | 0.02172 | finn-b-G6_FEMLE |
| "Carcinoma in situ of skin of upper limb, including shoulder" (all cancers excluded) | 14 | 94795202 | rs9989237 | T | C | 0.2081 | NA | 0.3102 | 0.1353 | 0.02182 | finn-b-CD2_INSITU_SKIN_UPPERLIMB_EXALLC |
| Interleukin-7 receptor subunit alpha | 14 | 94795202 | rs9989237 | T | C | 0.21768 | 3301 | -0.0688 | 0.03 | 0.021878 | prot-a-1544 |
| Protein kinase C-binding protein NELL1 | 14 | 94795202 | rs9989237 | T | C | 0.21768 | 3301 | 0.0689 | 0.0301 | 0.021878 | prot-a-2030 |
| Protein sel-1 homolog 2 | 14 | 94795202 | rs9989237 | T | C | 0.21768 | 3301 | -0.0689 | 0.03 | 0.021878 | prot-a-2664 |
| CMRF35-like molecule 2 | 14 | 94795202 | rs9989237 | T | C | 0.21768 | 3301 | -0.069 | 0.03 | 0.021878 | prot-a-435 |
| Cathepsin Z | 14 | 94795202 | rs9989237 | T | C | 0.21768 | 3301 | -0.0689 | 0.03 | 0.021878 | prot-a-729 |
| Transforming growth factor beta-1 | 14 | 94795202 | rs9989237 | T | C | 0.21768 | 3301 | 0.0688 | 0.03 | 0.021878 | prot-a-2962 |
| IDP T1 FAST ROIs L frontal pole | 14 | 94795202 | rs9989237 | T | C | 0.21004 | 7916 | 0.0293 | 0.0128 | 0.021878 | ubm-a-26 |
| CD28+ CD4-CD8- T cell Absolute Count | 14 | 94795202 | rs9989237 | T | C | 0.2654 | 3408 | 0.05763 | 0.02513 | 0.0219 | ebi-a-GCST90001657 |
| Lumbosacral root disorders, not elsewhere classified | 14 | 94795202 | rs9989237 | T | C | 0.2082 | NA | 0.2601 | 0.1136 | 0.02197 | finn-b-G6_LSROOT |
| Benign neoplasm: Pancreas | 14 | 94795202 | rs9989237 | T | C | 0.2083 | NA | 0.3128 | 0.1366 | 0.02199 | finn-b-CD2_BENIGN_PANCREAS |
| Total lipids in large HDL | 14 | 94795202 | rs9989237 | T | C | 0.20942 | NA | 0.008964 | 0.00447 | 0.022 | met-d-L_HDL_L |
| Phospholipids in small HDL | 14 | 94795202 | rs9989237 | T | C | 0.20942 | NA | -0.01256 | 0.005044 | 0.022 | met-d-S_HDL_PL |
| Fruit consumers | 14 | 94795202 | rs9989237 | T | C | 0.210565 | 64949 | -0.006 | 0.002623 | 0.022 | ukb-b-17554 |
| Operation code: carpal tunnel surgery | 14 | 94795202 | rs9989237 | T | C | 0.209974 | 462933 | 0.000593 | 0.00026 | 0.022 | ukb-b-17788 |
| Glomerular disorders in diseases classified elsewhere | 14 | 94795202 | rs9989237 | T | C | 0.2083 | NA | -0.0984 | 0.0429 | 0.02202 | finn-b-N14_GLOMEINOTH |
| Z87 Personal history of other diseases and conditions | 14 | 94795202 | rs9989237 | T | C | 0.6124 | NA | 0.1363 | 0.05959 | 0.02217 | ukb-e-Z87_CSA |
| epilepsy, all documented cases | 14 | 94795202 | rs9989237 | T | C | 0.223 | 44889 | 0.019792 | 0.008658 | 0.02223 | ieu-b-8 |
| ER membrane protein complex subunit 1 | 14 | 94795202 | rs9989237 | T | C | 0.21768 | 3301 | 0.0685 | 0.0301 | 0.022387 | prot-a-935 |
| Single-pass membrane and coiled-coil domain-containing protein 2 | 14 | 94795202 | rs9989237 | T | C | 0.21768 | 3301 | 0.0686 | 0.0301 | 0.022387 | prot-a-2775 |
| Acidic leucine-rich nuclear phosphoprotein 32 family member B | 14 | 94795202 | rs9989237 | T | C | 0.21768 | 3301 | 0.0685 | 0.0301 | 0.022387 | prot-a-105 |
| Protein disulfide-isomerase A5 | 14 | 94795202 | rs9989237 | T | C | 0.21768 | 3301 | 0.0686 | 0.03 | 0.022387 | prot-a-2233 |
| Rho GTPase-activating protein 1 | 14 | 94795202 | rs9989237 | T | C | 0.21768 | 3301 | -0.0686 | 0.03 | 0.022387 | prot-a-147 |
| Dynein light chain Tctex-type 3 | 14 | 94795202 | rs9989237 | T | C | 0.21768 | 3301 | 0.0687 | 0.03 | 0.022387 | prot-a-881 |
| a2009s lh G oc-temp lat-fusifor area | 14 | 94795202 | rs9989237 | T | C | 0.21004 | 7916 | 0.0372 | 0.0163 | 0.022387 | ubm-a-2767 |
| a2009s lh G precentral thickness | 14 | 94795202 | rs9989237 | T | C | 0.21004 | 7916 | -0.0402 | 0.0176 | 0.022387 | ubm-a-2987 |
| a2009s lh G postcentral thickness | 14 | 94795202 | rs9989237 | T | C | 0.21004 | 7916 | -0.0403 | 0.0176 | 0.022387 | ubm-a-2986 |
| Other arthritis (FG) | 14 | 94795202 | rs9989237 | T | C | 0.2081 | NA | 0.0576 | 0.0252 | 0.0224 | finn-b-RHEU_ARTHRITIS_OTH |
| Organic, including symptomatic, mental disorders | 14 | 94795202 | rs9989237 | T | C | 0.2083 | NA | 0.0459 | 0.0201 | 0.02246 | finn-b-F5_ORGANIC |
| NA | 14 | 94795202 | rs9989237 | T | C | 0.5719 | NA | -0.2203 | 0.09658 | 0.02255 | ukb-e-recode338_EAS |
| Country of Birth (non-UK origin) | 14 | 94795202 | rs9989237 | T | C | 0.5697 | NA | -0.2183 | 0.09572 | 0.02257 | ukb-e-20115_p22_EAS |
| Number of cigarettes smoked daily, combined previous and current smoking | 14 | 94795202 | rs9989237 | T | C | 0.7917 | NA | 0.0808 | 0.03545 | 0.02266 | ukb-e-recode2_AFR |
| Beta-defensin 103 | 14 | 94795202 | rs9989237 | T | C | 0.21768 | 3301 | 0.0685 | 0.0301 | 0.022909 | prot-a-781 |
| Dipeptidyl peptidase 2 | 14 | 94795202 | rs9989237 | T | C | 0.21768 | 3301 | 0.0684 | 0.0301 | 0.022909 | prot-a-860 |
| Type 2 lactosamine alpha-2,3-sialyltransferase | 14 | 94795202 | rs9989237 | T | C | 0.21768 | 3301 | 0.0684 | 0.03 | 0.022909 | prot-a-2850 |
| Piwi-like protein 1 | 14 | 94795202 | rs9989237 | T | C | 0.21768 | 3301 | 0.0684 | 0.03 | 0.022909 | prot-a-2280 |
| DKTatlas lh precentral thickness | 14 | 94795202 | rs9989237 | T | C | 0.21004 | 7916 | -0.0394 | 0.0173 | 0.022909 | ubm-a-2948 |
| Memory B cell Absolute Count | 14 | 94795202 | rs9989237 | T | C | 0.2674 | 3656 | -0.0658 | 0.02891 | 0.02292 | ebi-a-GCST90001407 |
| Apolipoprotein B | 14 | 94795202 | rs9989237 | T | C | 0.20942 | NA | -0.01105 | 0.005082 | 0.023 | met-d-ApoB |
| Phospholipids in large HDL | 14 | 94795202 | rs9989237 | T | C | 0.20942 | NA | 0.008875 | 0.004483 | 0.023 | met-d-L_HDL_PL |
| Total cholesterol minus HDL-C | 14 | 94795202 | rs9989237 | T | C | 0.20942 | NA | -0.01099 | 0.005061 | 0.023 | met-d-non_HDL_C |
| Triglycerides to total lipids ratio in small HDL | 14 | 94795202 | rs9989237 | T | C | 0.20942 | NA | -0.01096 | 0.004845 | 0.023 | met-d-S_HDL_TG_pct |
| Cholesterol to total lipids ratio in medium LDL | 14 | 94795202 | rs9989237 | T | C | 0.20942 | NA | -0.01104 | 0.005092 | 0.023 | met-d-M_LDL_C_pct |
| Frequency of heavy DIY in last 4 weeks | 14 | 94795202 | rs9989237 | T | C | 0.210192 | 182511 | 0.010985 | 0.004834 | 0.023 | ukb-b-5053 |
| Operation code: appendicectomy | 14 | 94795202 | rs9989237 | T | C | 0.209974 | 462933 | -0.00188 | 0.000828 | 0.023 | ukb-b-14944 |
| Wants to stop smoking | 14 | 94795202 | rs9989237 | T | C | 0.21078 | 35536 | 0.018145 | 0.008001 | 0.023 | ukb-b-3709 |
| PCT responsible for patient data: ROTHERHAM PCT | 14 | 94795202 | rs9989237 | T | C | 0.209969 | 463010 | 0.0004 | 0.000175 | 0.023 | ukb-b-18264 |
| IgD+ CD38- B cell Absolute Count | 14 | 94795202 | rs9989237 | T | C | 0.2674 | 3656 | -0.06578 | 0.02893 | 0.02303 | ebi-a-GCST90001396 |
| Age at menopause | 14 | 94795202 | rs9989237 | T | C | NA | 11859 | 0.1566 | 0.0689 | 0.02304 | ieu-b-4824 |
| Carcinoma in situ of skin of upper limb, including shoulder | 14 | 94795202 | rs9989237 | T | C | 0.2083 | NA | 0.3066 | 0.135 | 0.02317 | finn-b-CD2_INSITU_SKIN_UPPERLIMB |
| Phosphate | 14 | 94795202 | rs9989237 | T | C | 0.20995 | 314658 | -0.00678 | 0.002986 | 0.02327 | ukb-d-30810_irnt |
| Valine--tRNA ligase | 14 | 94795202 | rs9989237 | T | C | 0.21768 | 3301 | -0.0681 | 0.03 | 0.023442 | prot-a-3191 |
| WW domain-binding protein 1 | 14 | 94795202 | rs9989237 | T | C | 0.21768 | 3301 | 0.068 | 0.03 | 0.023442 | prot-a-3218 |
| IDP T1 SIENAX peripheral grey normalised volume | 14 | 94795202 | rs9989237 | T | C | 0.21004 | 7916 | 0.0312 | 0.0138 | 0.023442 | ubm-a-1 |
| DNA-(apurinic or apyrimidinic site) lyase levels | 14 | 94795202 | rs9989237 | T | C | 0.233 | 1187 | 0.110667 | 0.048724 | 0.023599 | ebi-a-GCST90010284 |
| Other retinal disorders | 14 | 94795202 | rs9989237 | T | C | 0.6092 | NA | -0.2033 | 0.08989 | 0.02369 | ukb-e-362_CSA |
| NA | 14 | 94795202 | rs9989237 | T | C | 0.7912 | NA | -0.2307 | 0.102 | 0.02372 | ukb-e-recode436_AFR |
| NA | 14 | 94795202 | rs9989237 | T | C | 0.7912 | NA | -0.2307 | 0.102 | 0.02372 | ukb-e-recode286_AFR |
| Birth weight known | 14 | 94795202 | rs9989237 | T | C | 0.5689 | NA | -0.2377 | 0.1052 | 0.02383 | ukb-e-120_p2_EAS |
| Operative procedures - main OPCS4 | 14 | 94795202 | rs9989237 | T | C | 0.7945 | NA | 0.2639 | 0.1169 | 0.02393 | ukb-e-41200_p702_AFR |
| Melanocytic naevi of other and unspecified parts of face | 14 | 94795202 | rs9989237 | T | C | 0.2083 | NA | -0.1376 | 0.061 | 0.02397 | finn-b-CD2_BENIGN_MELANOCYTIC_FACE |
| Glioma pathogenesis-related protein 1 | 14 | 94795202 | rs9989237 | T | C | 0.21768 | 3301 | 0.0679 | 0.0301 | 0.023988 | prot-a-1217 |
| IDP SWI T2star right accumbens | 14 | 94795202 | rs9989237 | T | C | 0.21004 | 7916 | 0.0436 | 0.0193 | 0.023988 | ubm-a-179 |
| NET100 0915 | 14 | 94795202 | rs9989237 | T | C | 0.21004 | 7916 | -0.0437 | 0.0194 | 0.023988 | ubm-a-2071 |
| LDL cholesterol | 14 | 94795202 | rs9989237 | T | C | 0.20942 | NA | -0.01078 | 0.005067 | 0.024 | met-d-LDL_C |
| Free cholesterol in medium LDL | 14 | 94795202 | rs9989237 | T | C | 0.20942 | NA | -0.01058 | 0.005082 | 0.024 | met-d-M_LDL_FC |
| Oral cavity cancer | 14 | 94795202 | rs9989237 | T | C | 0.209892 | 372373 | 0.000199 | 8.80E-05 | 0.024 | ieu-b-4961 |
| Operative procedures - secondary OPCS: Y50.8 Other specified approach through abdominal cavity | 14 | 94795202 | rs9989237 | T | C | 0.209969 | 463010 | -0.00073 | 0.000323 | 0.024 | ukb-b-9961 |
| Diagnoses - main ICD10: K20 Oesophagitis | 14 | 94795202 | rs9989237 | T | C | 0.209969 | 463010 | 0.00059 | 0.000262 | 0.024 | ukb-b-19354 |
| Type 1 diabetes with neurological complications | 14 | 94795202 | rs9989237 | T | C | 0.2077 | NA | -0.1646 | 0.0729 | 0.02405 | finn-b-E4_DM1NEU |
| Leisure/social activities | 14 | 94795202 | rs9989237 | T | C | 0.7913 | NA | -0.1849 | 0.08199 | 0.0241 | ukb-e-6160_p5_AFR |
| TestASV_21 (Ruminococcaceae) prevalence | 14 | 94795202 | rs9989237 | T | C | NA | 8956 | 0.095513 | 0.042358 | 0.024139 | ebi-a-GCST90011695 |
| Diagnoses - main ICD10: H25 Senile cataract | 14 | 94795202 | rs9989237 | T | C | 0.209385 | 337199 | -0.0006 | 0.000264 | 0.024295 | ukb-a-529 |
| Skin changes due to chronic exposure to nonionizing radiation | 14 | 94795202 | rs9989237 | T | C | 0.2083 | NA | -0.0593 | 0.0264 | 0.02439 | finn-b-L12_NONIONRADISKIN |
| focal epilepsy, all documented cases | 14 | 94795202 | rs9989237 | T | C | 0.2252 | 39348 | 0.022616 | 0.010056 | 0.02453 | ieu-b-10 |
| Bone morphogenetic protein 6 | 14 | 94795202 | rs9989237 | T | C | 0.21768 | 3301 | -0.0675 | 0.03 | 0.024547 | prot-a-256 |
| Bcl-2-like protein 2 | 14 | 94795202 | rs9989237 | T | C | 0.21768 | 3301 | 0.0676 | 0.0301 | 0.024547 | prot-a-240 |
| Pro-FMRFamide-related neuropeptide FF | 14 | 94795202 | rs9989237 | T | C | 0.21768 | 3301 | -0.0676 | 0.0301 | 0.024547 | prot-a-2073 |
| T-lymphocyte activation antigen CD80 | 14 | 94795202 | rs9989237 | T | C | 0.21768 | 3301 | 0.0676 | 0.03 | 0.024547 | prot-a-463 |
| Polyadenylate-binding protein 3 | 14 | 94795202 | rs9989237 | T | C | 0.21768 | 3301 | -0.0676 | 0.03 | 0.024547 | prot-a-2163 |
| Leukemia inhibitory factor receptor | 14 | 94795202 | rs9989237 | T | C | 0.21768 | 3301 | -0.0677 | 0.03 | 0.024547 | prot-a-1737 |
| Carboxypeptidase A2 | 14 | 94795202 | rs9989237 | T | C | 0.21768 | 3301 | -0.0675 | 0.03 | 0.024547 | prot-a-642 |
| NET25 0125 | 14 | 94795202 | rs9989237 | T | C | 0.21004 | 7916 | 0.0432 | 0.0192 | 0.024547 | ubm-a-1071 |
| IDP dMRI TBSS MD Fornix | 14 | 94795202 | rs9989237 | T | C | 0.21004 | 7916 | -0.0369 | 0.0164 | 0.024547 | ubm-a-249 |
| NET100 0684 | 14 | 94795202 | rs9989237 | T | C | 0.21004 | 7916 | 0.0436 | 0.0194 | 0.024547 | ubm-a-1840 |
| a2009s rh S postcentral area | 14 | 94795202 | rs9989237 | T | C | 0.21004 | 7916 | 0.0362 | 0.0161 | 0.024547 | ubm-a-2919 |
| NET100 0764 | 14 | 94795202 | rs9989237 | T | C | 0.21004 | 7916 | -0.0429 | 0.0191 | 0.024547 | ubm-a-1920 |
| COVID-19 (very severe respiratory confirmed vs population) RELEASE 5 | 14 | 94795202 | rs9989237 | T | C | 0.2105 | 1059456 | 0.0875 | 0.038943 | 0.02465 | ebi-a-GCST011077 |
| Type of sliced bread eaten | 14 | 94795202 | rs9989237 | T | C | 0.788 | NA | 0.2843 | 0.1265 | 0.02465 | ukb-e-20091_p3_AFR |
| Phosphate | 14 | 94795202 | rs9989237 | T | C | 0.20995 | 314658 | -0.00108 | 0.00048 | 0.02473 | ukb-d-30810_raw |
| Hearing aid user | 14 | 94795202 | rs9989237 | T | C | 0.209385 | 204240 | -0.00189 | 0.000841 | 0.024807 | ukb-a-340 |
| Crohn disease (strict definition, require KELA) | 14 | 94795202 | rs9989237 | T | C | 0.2083 | NA | -0.1353 | 0.0603 | 0.02494 | finn-b-K11_CD_STRICT |
| Free cholesterol in small HDL | 14 | 94795202 | rs9989237 | T | C | 0.20942 | NA | -0.01132 | 0.004995 | 0.025 | met-d-S_HDL_FC |
| Longest period of unenthusiasm / disinterest | 14 | 94795202 | rs9989237 | T | C | 0.21102 | 41182 | 0.018795 | 0.008414 | 0.025 | ukb-b-6392 |
| Pustulosis palmaris et plantaris | 14 | 94795202 | rs9989237 | T | C | 0.2084 | NA | 0.1709 | 0.0763 | 0.02503 | finn-b-L12_PSORI_PUSTUPALM |
| Myeloperoxidase-DNA complexes | 14 | 94795202 | rs9989237 | T | C | NA | 5590 | -0.0429 | 0.0192 | 0.02508 | ebi-a-GCST90013658 |
| Immunoglobulin superfamily member 8 | 14 | 94795202 | rs9989237 | T | C | 0.21768 | 3301 | 0.0674 | 0.0301 | 0.025119 | prot-a-1462 |
| Eukaryotic translation initiation factor 4 gamma 3 | 14 | 94795202 | rs9989237 | T | C | 0.21768 | 3301 | 0.0673 | 0.0301 | 0.025119 | prot-a-925 |
| mRNA-capping enzyme | 14 | 94795202 | rs9989237 | T | C | 0.21768 | 3301 | 0.0674 | 0.0301 | 0.025119 | prot-a-2574 |
| Endoplasmic reticulum lectin 1 | 14 | 94795202 | rs9989237 | T | C | 0.21768 | 3301 | -0.0672 | 0.0301 | 0.025119 | prot-a-979 |
| Bone morphogenetic protein 10 | 14 | 94795202 | rs9989237 | T | C | 0.21768 | 3301 | 0.0673 | 0.03 | 0.025119 | prot-a-254 |
| DKTatlas rh middletemporal area | 14 | 94795202 | rs9989237 | T | C | 0.21004 | 7916 | 0.0294 | 0.0131 | 0.025119 | ubm-a-2833 |
| Malignant neoplasm of ovary (all cancers excluded) | 14 | 94795202 | rs9989237 | T | C | 0.2079 | NA | -0.1483 | 0.0662 | 0.02513 | finn-b-C3_OVARY_EXALLC |
| Siatica+with lumbago | 14 | 94795202 | rs9989237 | T | C | 0.208 | NA | 0.0443 | 0.0198 | 0.02517 | finn-b-M13_SCIATICA |
| NA | 14 | 94795202 | rs9989237 | T | C | 0.7916 | NA | -0.2828 | 0.1265 | 0.02535 | ukb-e-recode74_AFR |
| Duration of walks | 14 | 94795202 | rs9989237 | T | C | 0.7957 | NA | 0.05777 | 0.02586 | 0.02546 | ukb-e-874_p1_AFR |
| OTU99_106 (Barnesiella) prevalence | 14 | 94795202 | rs9989237 | T | C | NA | 8956 | 0.096244 | 0.043084 | 0.025492 | ebi-a-GCST90011621 |
| Benign neoplasm: Other/unspecified site (all cancers excluded) | 14 | 94795202 | rs9989237 | T | C | 0.2081 | NA | 0.2626 | 0.1177 | 0.02563 | finn-b-CD2_BENIGN_NOS_EXALLC |
| Stanniocalcin-1 | 14 | 94795202 | rs9989237 | T | C | 0.21768 | 3301 | -0.067 | 0.03 | 0.025704 | prot-a-2873 |
| M-phase inducer phosphatase 2 | 14 | 94795202 | rs9989237 | T | C | 0.21768 | 3301 | 0.0671 | 0.0301 | 0.025704 | prot-a-472 |
| Lymphocyte activation gene 3 protein | 14 | 94795202 | rs9989237 | T | C | 0.21768 | 3301 | -0.0669 | 0.03 | 0.025704 | prot-a-1693 |
| IDP T1 SIENAX peripheral grey unnormalised volume | 14 | 94795202 | rs9989237 | T | C | 0.21004 | 7916 | 0.021 | 0.0094 | 0.025704 | ubm-a-2 |
| NET100 1393 | 14 | 94795202 | rs9989237 | T | C | 0.21004 | 7916 | 0.043 | 0.0193 | 0.025704 | ubm-a-2549 |
| OTU99_106 (Barnesiella) abundance | 14 | 94795202 | rs9989237 | T | C | NA | 8956 | -0.08127 | 0.036456 | 0.025796 | ebi-a-GCST90011420 |
| Starch | 14 | 94795202 | rs9989237 | T | C | 0.6217 | NA | 0.105 | 0.0471 | 0.02586 | ukb-e-100023_CSA |
| 3-Hydroxybutyrate | 14 | 94795202 | rs9989237 | T | C | 0.20921 | NA | 0.011335 | 0.005083 | 0.026 | met-d-bOHbutyrate |
| Remnant cholesterol (non-HDL, non-LDL -cholesterol) | 14 | 94795202 | rs9989237 | T | C | 0.20942 | NA | -0.01095 | 0.005056 | 0.026 | met-d-Remnant_C |
| Total lipids in chylomicrons and extremely large VLDL | 14 | 94795202 | rs9989237 | T | C | 0.20942 | NA | -0.01044 | 0.004905 | 0.026 | met-d-XXL_VLDL_L |
| Free cholesterol to total lipids ratio in IDL | 14 | 94795202 | rs9989237 | T | C | 0.20942 | NA | -0.01087 | 0.005049 | 0.026 | met-d-IDL_FC_pct |
| Concentration of LDL particles | 14 | 94795202 | rs9989237 | T | C | 0.20942 | NA | -0.01081 | 0.005085 | 0.026 | met-d-LDL_P |
| Crohn's disease vs ulcerative colitis (ordinary least squares (OLS)) | 14 | 94795202 | rs9989237 | T | C | NA | 12924 | -0.017 | 0.0076 | 0.026 | ebi-a-GCST90016611 |
| 15-hydroxyprostaglandin dehydrogenase [NAD(+)] | 14 | 94795202 | rs9989237 | T | C | 0.21768 | 3301 | -0.0667 | 0.03 | 0.026303 | prot-a-1370 |
| Alpha-N-acetylgalactosaminide alpha-2,6-sialyltransferase 6 | 14 | 94795202 | rs9989237 | T | C | 0.21768 | 3301 | 0.0668 | 0.0301 | 0.026303 | prot-a-2857 |
| T-cell surface glycoprotein CD8 beta chain | 14 | 94795202 | rs9989237 | T | C | 0.21768 | 3301 | -0.0666 | 0.03 | 0.026303 | prot-a-467 |
| Suppressor of cytokine signaling 7 | 14 | 94795202 | rs9989237 | T | C | 0.21768 | 3301 | 0.0667 | 0.0301 | 0.026303 | prot-a-2798 |
| Zinc finger protein 10 | 14 | 94795202 | rs9989237 | T | C | 0.21768 | 3301 | 0.0668 | 0.0301 | 0.026303 | prot-a-3259 |
| Glutathione S-transferase theta-2B | 14 | 94795202 | rs9989237 | T | C | 0.21768 | 3301 | 0.0667 | 0.03 | 0.026303 | prot-a-1291 |
| Zinc finger protein 329 | 14 | 94795202 | rs9989237 | T | C | 0.21768 | 3301 | 0.0667 | 0.03 | 0.026303 | prot-a-3268 |
| Spells in hospital | 14 | 94795202 | rs9989237 | T | C | 0.7956 | NA | 0.04596 | 0.02071 | 0.02644 | ukb-e-41235_AFR |
| Neurotic, stress-related and somatoform disorders | 14 | 94795202 | rs9989237 | T | C | 0.2083 | NA | 0.0308 | 0.0139 | 0.0265 | finn-b-F5_NEUROTIC |
| Speech disturbances, not elsewhere classified | 14 | 94795202 | rs9989237 | T | C | 0.2084 | NA | 0.1187 | 0.0535 | 0.02659 | finn-b-R18_SPEECH_DISTU_NOT_ELSEW_CLASSIFIED |
| Vitamin and mineral supplements: Vitamin A | 14 | 94795202 | rs9989237 | T | C | 0.209385 | 335591 | 0.000894 | 0.000403 | 0.026619 | ukb-a-458 |
| Glucose | 14 | 94795202 | rs9989237 | T | C | 0.5792 | NA | -0.06533 | 0.0295 | 0.02676 | ukb-e-30740_EAS |
| Tyrosine-protein kinase ABL1 | 14 | 94795202 | rs9989237 | T | C | 0.21768 | 3301 | 0.0665 | 0.0301 | 0.026915 | prot-a-7 |
| Neuropeptide W | 14 | 94795202 | rs9989237 | T | C | 0.21768 | 3301 | -0.0665 | 0.0301 | 0.026915 | prot-a-2082 |
| NET100 0451 | 14 | 94795202 | rs9989237 | T | C | 0.21004 | 7916 | -0.0427 | 0.0193 | 0.026915 | ubm-a-1607 |
| Melanocytic naevi of other and unspecified parts of face (all cancers excluded) | 14 | 94795202 | rs9989237 | T | C | 0.2081 | NA | -0.1349 | 0.061 | 0.02695 | finn-b-CD2_BENIGN_MELANOCYTIC_FACE_EXALLC |
| Operative procedures - secondary OPCS: Z40.1 Pulmonary artery | 14 | 94795202 | rs9989237 | T | C | 0.209969 | 463010 | 0.000363 | 0.000164 | 0.027 | ukb-b-18616 |
| Banana intake | 14 | 94795202 | rs9989237 | T | C | 0.210565 | 64949 | -0.01598 | 0.007224 | 0.027 | ukb-b-5362 |
| Gut microbiota abundance (genus Lachnospiraceae UCG001 id.11321) | 14 | 94795202 | rs9989237 | T | C | NA | 14306 | -0.03789 | 0.017004 | 0.027038 | ebi-a-GCST90017025 |
| Illnesses of father: Chronic bronchitis/emphysema | 14 | 94795202 | rs9989237 | T | C | 0.209385 | 292053 | 0.002191 | 0.000992 | 0.02719 | ukb-a-206 |
| IgD+ CD24+ B cell Absolute Count | 14 | 94795202 | rs9989237 | T | C | 0.2674 | 3656 | -0.06254 | 0.02831 | 0.02721 | ebi-a-GCST90001412 |
| Glomerular diseases | 14 | 94795202 | rs9989237 | T | C | 0.2083 | NA | -0.0614 | 0.0278 | 0.02725 | finn-b-N14_GLOMERULAR |
| Benign neoplasm: Other/unspecified site | 14 | 94795202 | rs9989237 | T | C | 0.2083 | NA | 0.2596 | 0.1176 | 0.02729 | finn-b-CD2_BENIGN_NOS |
| OTU97_38 (Alphaproteobacteria) prevalence | 14 | 94795202 | rs9989237 | T | C | NA | 8956 | 0.126518 | 0.057369 | 0.02743 | ebi-a-GCST90011597 |
| Witnessed sudden violent death | 14 | 94795202 | rs9989237 | T | C | 0.209903 | 117862 | -0.00377 | 0.001707 | 0.027444 | ukb-d-20530 |
| Atypical mycobacterium lung infection | 14 | 94795202 | rs9989237 | T | C | 0.2083 | NA | 0.3237 | 0.1468 | 0.02745 | finn-b-MYCOBLUNGATYPICA |
| Leukocyte immunoglobulin-like receptor subfamily B member 4 | 14 | 94795202 | rs9989237 | T | C | 0.21768 | 3301 | 0.0662 | 0.0301 | 0.027542 | prot-a-1746 |
| Low-density lipoprotein receptor-related protein 1B | 14 | 94795202 | rs9989237 | T | C | 0.21768 | 3301 | -0.0662 | 0.03 | 0.027542 | prot-a-1779 |
| Ras-related protein Rab-14 | 14 | 94795202 | rs9989237 | T | C | 0.21768 | 3301 | -0.0663 | 0.0301 | 0.027542 | prot-a-2471 |
| Fibroblast growth factor 8 isoform B | 14 | 94795202 | rs9989237 | T | C | 0.21768 | 3301 | 0.0662 | 0.03 | 0.027542 | prot-a-1099 |
| DKTatlas lh paracentral area | 14 | 94795202 | rs9989237 | T | C | 0.21004 | 7916 | 0.0332 | 0.0151 | 0.027542 | ubm-a-2729 |
| NET100 0924 | 14 | 94795202 | rs9989237 | T | C | 0.21004 | 7916 | 0.0423 | 0.0192 | 0.027542 | ubm-a-2080 |
| a2009s rh S circular insula sup area | 14 | 94795202 | rs9989237 | T | C | 0.21004 | 7916 | 0.0333 | 0.0151 | 0.027542 | ubm-a-2901 |
| Lichen sclerosus et atrophicus | 14 | 94795202 | rs9989237 | T | C | 0.2083 | NA | 0.1218 | 0.0553 | 0.02769 | finn-b-L12_LICHENSCLERATROPH |
| Plasma Blast-Plasma Cell %B cell | 14 | 94795202 | rs9989237 | T | C | 0.2674 | 3658 | 0.06242 | 0.02835 | 0.02775 | ebi-a-GCST90001404 |
| Varicella zoster virus glycoproteins E and I antibody levels | 14 | 94795202 | rs9989237 | T | C | 0.21564 | 7595 | 0.043175 | 0.019631 | 0.027858 | ebi-a-GCST90006929 |
| Direct bilirubin | 14 | 94795202 | rs9989237 | T | C | 0.577 | NA | -0.06502 | 0.02957 | 0.02786 | ukb-e-30660_EAS |
| Mother's age | 14 | 94795202 | rs9989237 | T | C | 0.6088 | NA | 0.03532 | 0.01607 | 0.02796 | ukb-e-1845_CSA |
| Protein ripply1 | 14 | 94795202 | rs9989237 | T | C | 0.21768 | 3301 | -0.0659 | 0.03 | 0.028184 | prot-a-2547 |
| Collectin-10 | 14 | 94795202 | rs9989237 | T | C | 0.21768 | 3301 | -0.066 | 0.03 | 0.028184 | prot-a-627 |
| Membrane metallo-endopeptidase-like 1 | 14 | 94795202 | rs9989237 | T | C | 0.21768 | 3301 | 0.066 | 0.03 | 0.028184 | prot-a-1908 |
| Serine/threonine-protein kinase PLK1 | 14 | 94795202 | rs9989237 | T | C | 0.21768 | 3301 | 0.0659 | 0.0301 | 0.028184 | prot-a-2303 |
| NAD-dependent protein deacylase sirtuin-5, mitochondrial | 14 | 94795202 | rs9989237 | T | C | 0.21768 | 3301 | 0.0659 | 0.0301 | 0.028184 | prot-a-2737 |
| Midkine | 14 | 94795202 | rs9989237 | T | C | 0.21768 | 3301 | 0.0658 | 0.03 | 0.028184 | prot-a-1871 |
| a2009s lh S occipital ant thickness | 14 | 94795202 | rs9989237 | T | C | 0.21004 | 7916 | -0.0391 | 0.0178 | 0.028184 | ubm-a-3017 |
| NET25 0096 | 14 | 94795202 | rs9989237 | T | C | 0.21004 | 7916 | 0.0419 | 0.0191 | 0.028184 | ubm-a-1042 |
| Open wound of hip and thigh | 14 | 94795202 | rs9989237 | T | C | 0.2083 | NA | -0.1866 | 0.0852 | 0.02853 | finn-b-ST19_OPEN_WOUND_HIP_THIGH |
| plasminogen activator, tissue type | 14 | 94795202 | rs9989237 | T | C | 0.2384 | 3394 | -0.0687 | 0.0314 | 0.02882 | prot-b-62 |
| Netrin receptor UNC5C | 14 | 94795202 | rs9989237 | T | C | 0.21768 | 3301 | -0.0657 | 0.0301 | 0.02884 | prot-a-3168 |
| Nuclear receptor subfamily 4 group A member 1 | 14 | 94795202 | rs9989237 | T | C | 0.21768 | 3301 | 0.0656 | 0.0301 | 0.02884 | prot-a-2090 |
| Platelet-activating factor acetylhydrolase IB subunit beta | 14 | 94795202 | rs9989237 | T | C | 0.21768 | 3301 | -0.0658 | 0.0301 | 0.02884 | prot-a-2166 |
| RING finger protein 148 | 14 | 94795202 | rs9989237 | T | C | 0.21768 | 3301 | 0.0657 | 0.03 | 0.02884 | prot-a-2564 |
| Pleiotrophin | 14 | 94795202 | rs9989237 | T | C | 0.21768 | 3301 | 0.0657 | 0.0301 | 0.02884 | prot-a-2435 |
| NODEamps25 0015 | 14 | 94795202 | rs9989237 | T | C | 0.21004 | 7916 | 0.0403 | 0.0184 | 0.02884 | ubm-a-885 |
| NET100 1068 | 14 | 94795202 | rs9989237 | T | C | 0.21004 | 7916 | 0.0421 | 0.0193 | 0.02884 | ubm-a-2224 |
| a2009s rh G occipital sup area | 14 | 94795202 | rs9989237 | T | C | 0.21004 | 7916 | 0.0358 | 0.0164 | 0.02884 | ubm-a-2872 |
| Gut microbiota abundance (genus Oscillibacter id.2063) | 14 | 94795202 | rs9989237 | T | C | NA | 14306 | 0.04114 | 0.018017 | 0.028941 | ebi-a-GCST90017036 |
| Histidine | 14 | 94795202 | rs9989237 | T | C | 0.211834 | 19242 | 0.027462 | 0.012522 | 0.029213 | met-c-866 |
| 6mm index of best keratometry results (left) | 14 | 94795202 | rs9989237 | T | C | 0.6115 | NA | 0.03839 | 0.01764 | 0.02949 | ukb-e-5306_CSA |
| Artrosis, including avohilmo | 14 | 94795202 | rs9989237 | T | C | 0.2083 | NA | 0.0247 | 0.0114 | 0.02951 | finn-b-M13_ARTHROSIS_INCLAVO |
| Neurotensin/neuromedin N | 14 | 94795202 | rs9989237 | T | C | 0.21768 | 3301 | -0.0654 | 0.0301 | 0.029512 | prot-a-2124 |
| Neural proliferation differentiation and control protein 1 | 14 | 94795202 | rs9989237 | T | C | 0.21768 | 3301 | -0.0653 | 0.0301 | 0.029512 | prot-a-2072 |
| Matrix metalloproteinase-14 | 14 | 94795202 | rs9989237 | T | C | 0.21768 | 3301 | -0.0655 | 0.0301 | 0.029512 | prot-a-1914 |
| 39S ribosomal protein L32, mitochondrial | 14 | 94795202 | rs9989237 | T | C | 0.21768 | 3301 | -0.0653 | 0.0301 | 0.029512 | prot-a-1941 |
| Beta-defensin 108B | 14 | 94795202 | rs9989237 | T | C | 0.21768 | 3301 | 0.0654 | 0.0301 | 0.029512 | prot-a-786 |
| SAGA-associated factor 29 homolog | 14 | 94795202 | rs9989237 | T | C | 0.21768 | 3301 | 0.0653 | 0.0301 | 0.029512 | prot-a-379 |
| Immunoglobulin superfamily DCC subclass member 4 | 14 | 94795202 | rs9989237 | T | C | 0.21768 | 3301 | 0.0654 | 0.03 | 0.029512 | prot-a-1442 |
| Chymase | 14 | 94795202 | rs9989237 | T | C | 0.21768 | 3301 | 0.0653 | 0.03 | 0.029512 | prot-a-597 |
| NET100 1075 | 14 | 94795202 | rs9989237 | T | C | 0.21004 | 7916 | -0.0419 | 0.0192 | 0.029512 | ubm-a-2231 |
| Sweetened cereal intake | 14 | 94795202 | rs9989237 | T | C | 0.6217 | NA | 0.02786 | 0.01282 | 0.02972 | ukb-e-100820_CSA |
| Primary coxarthrosis, bilateral | 14 | 94795202 | rs9989237 | T | C | 0.2082 | NA | 0.0622 | 0.0286 | 0.02973 | finn-b-PRIM_COXARTHROSIS |
| Bulimia nervosa | 14 | 94795202 | rs9989237 | T | C | 0.204 | 2442 | 0.019 | 0.009 | 0.02974 | ieu-a-990 |
| epithelial cell adhesion molecule levels | 14 | 94795202 | rs9989237 | T | C | 0.234 | 1323 | 0.100637 | 0.04624 | 0.029864 | ebi-a-GCST90010213 |
| Duration of fitness test | 14 | 94795202 | rs9989237 | T | C | 0.7915 | NA | 0.05943 | 0.02737 | 0.02991 | ukb-e-6039_AFR |
| Triglycerides in chylomicrons and extremely large VLDL | 14 | 94795202 | rs9989237 | T | C | 0.20942 | NA | -0.01043 | 0.004913 | 0.03 | met-d-XXL_VLDL_TG |
| Cholesterol in medium VLDL | 14 | 94795202 | rs9989237 | T | C | 0.20942 | NA | -0.01062 | 0.005074 | 0.03 | met-d-M_VLDL_C |
| Acetone | 14 | 94795202 | rs9989237 | T | C | 0.209422 | NA | 0.011035 | 0.005088 | 0.03 | met-d-Acetone |
| Saturated fatty acids | 14 | 94795202 | rs9989237 | T | C | 0.209439 | NA | -0.01129 | 0.005043 | 0.03 | met-d-SFA |
| Fatty acid-binding protein, adipocyte levels | 14 | 94795202 | rs9989237 | T | C | 0.2197 | 21758 | 0.024 | 0.0111 | 0.03011 | ebi-a-GCST90012075 |
| Other and unspcified rosacea | 14 | 94795202 | rs9989237 | T | C | 0.2083 | NA | -0.122 | 0.0563 | 0.03019 | finn-b-L12_ROSACEANAS |
| Keratin, type II cytoskeletal 7 | 14 | 94795202 | rs9989237 | T | C | 0.21768 | 3301 | -0.0651 | 0.03 | 0.0302 | prot-a-1686 |
| Hemojuvelin | 14 | 94795202 | rs9989237 | T | C | 0.21768 | 3301 | 0.0653 | 0.03 | 0.0302 | prot-a-1332 |
| Vesicular integral-membrane protein VIP36 | 14 | 94795202 | rs9989237 | T | C | 0.21768 | 3301 | -0.0651 | 0.03 | 0.0302 | prot-a-1755 |
| Retinoblastoma-like protein 1 | 14 | 94795202 | rs9989237 | T | C | 0.21768 | 3301 | 0.065 | 0.0301 | 0.0302 | prot-a-2500 |
| NET100 1469 | 14 | 94795202 | rs9989237 | T | C | 0.21004 | 7916 | 0.0423 | 0.0195 | 0.0302 | ubm-a-2625 |
| Other general symptoms and signs | 14 | 94795202 | rs9989237 | T | C | 0.2083 | NA | -0.1637 | 0.0755 | 0.0302 | finn-b-R18_OTHER_GENERAL_SYMPTOMS_SIGNS |
| Nervous feelings | 14 | 94795202 | rs9989237 | T | C | 0.7672 | NA | 0.2107 | 0.09724 | 0.03022 | ukb-e-1970_MID |
| Unspecified haematuria | 14 | 94795202 | rs9989237 | T | C | 0.2082 | NA | 0.0482 | 0.0223 | 0.03033 | finn-b-R18_UNSPE_HAEMATU |
| HLA DR+ Natural Killer %CD3- lymphocyte | 14 | 94795202 | rs9989237 | T | C | 0.2679 | 3596 | -0.06203 | 0.02865 | 0.03042 | ebi-a-GCST90001650 |
| Other meningitis | 14 | 94795202 | rs9989237 | T | C | 0.2083 | NA | 0.2794 | 0.1291 | 0.03046 | finn-b-G6_MENINGOTH |
| Certain infectious and parasitic diseases | 14 | 94795202 | rs9989237 | T | C | 0.2083 | NA | 0.0191 | 0.0088 | 0.03046 | finn-b-AB1_INFECTIONS |
| Vascular diseases of the intestine | 14 | 94795202 | rs9989237 | T | C | 0.2084 | NA | -0.1855 | 0.0859 | 0.0307 | finn-b-I9_VASCINT |
| Degeneration of macula and posterior pole | 14 | 94795202 | rs9989237 | T | C | 0.209437 | 361194 | -0.00033 | 0.000153 | 0.0307 | ukb-d-H7_MACULADEGEN |
| CD8 on Terminally Differentiated CD8+ T cell | 14 | 94795202 | rs9989237 | T | C | 0.2692 | 2911 | 0.0684 | 0.03168 | 0.03089 | ebi-a-GCST90002057 |
| Proline-rich protein 1 | 14 | 94795202 | rs9989237 | T | C | 0.21768 | 3301 | 0.0649 | 0.0301 | 0.030903 | prot-a-2383 |
| Histone deacetylase 8 | 14 | 94795202 | rs9989237 | T | C | 0.21768 | 3301 | -0.0648 | 0.0301 | 0.030903 | prot-a-1320 |
| Calcium/calmodulin-dependent protein kinase kinase 1 | 14 | 94795202 | rs9989237 | T | C | 0.21768 | 3301 | 0.0649 | 0.0301 | 0.030903 | prot-a-350 |
| Protein G6b | 14 | 94795202 | rs9989237 | T | C | 0.21768 | 3301 | -0.065 | 0.03 | 0.030903 | prot-a-1160 |
| Ephrin-A3 | 14 | 94795202 | rs9989237 | T | C | 0.21768 | 3301 | -0.0648 | 0.0301 | 0.030903 | prot-a-899 |
| NET100 0131 | 14 | 94795202 | rs9989237 | T | C | 0.21004 | 7916 | 0.0413 | 0.0191 | 0.030903 | ubm-a-1287 |
| NODEamps100 0017 | 14 | 94795202 | rs9989237 | T | C | 0.21004 | 7916 | 0.0384 | 0.0178 | 0.030903 | ubm-a-908 |
| a2009s lh Lat Fis-post thickness | 14 | 94795202 | rs9989237 | T | C | 0.21004 | 7916 | -0.0375 | 0.0174 | 0.030903 | ubm-a-2999 |
| volume Left-Cerebellum-White-Matter | 14 | 94795202 | rs9989237 | T | C | 0.21004 | 7916 | 0.0335 | 0.0156 | 0.030903 | ubm-a-2658 |
| E-selectin levels | 14 | 94795202 | rs9989237 | T | C | 0.234 | 1323 | 0.101606 | 0.046973 | 0.030921 | ebi-a-GCST90010258 |
| ENSG00000165959 | 14 | 94795202 | rs9989237 | T | C | 0.22464 | 14262 | 0.03077 | 0.014259 | 0.030928 | eqtl-a-ENSG00000165959 |
| Phospholipids in large LDL | 14 | 94795202 | rs9989237 | T | C | 0.20942 | NA | -0.01027 | 0.00505 | 0.031 | met-d-L_LDL_PL |
| Cholesterol to total lipids ratio in large HDL | 14 | 94795202 | rs9989237 | T | C | 0.20942 | NA | 0.010347 | 0.004801 | 0.031 | met-d-L_HDL_C_pct |
| Non-cancer illness code, self-reported: depression | 14 | 94795202 | rs9989237 | T | C | 0.209974 | 462933 | -0.00128 | 0.000593 | 0.031 | ukb-b-12064 |
| Pulses intake | 14 | 94795202 | rs9989237 | T | C | 0.210565 | 64949 | 0.010655 | 0.004952 | 0.031 | ukb-b-621 |
| Leg pain when walking uphill or hurrying | 14 | 94795202 | rs9989237 | T | C | 0.213576 | 32192 | -0.00958 | 0.00443 | 0.031 | ukb-b-3196 |
| Gut microbiota abundance (order Coriobacteriales id.810) | 14 | 94795202 | rs9989237 | T | C | NA | 14306 | -0.02753 | 0.012873 | 0.031211 | ebi-a-GCST90017096 |
| Gut microbiota abundance (class Coriobacteriia id.809) | 14 | 94795202 | rs9989237 | T | C | NA | 14306 | -0.02753 | 0.012873 | 0.031211 | ebi-a-GCST90016914 |
| Gut microbiota abundance (family Coriobacteriaceae id.811) | 14 | 94795202 | rs9989237 | T | C | NA | 14306 | -0.02753 | 0.012873 | 0.031211 | ebi-a-GCST90016933 |
| Ulcerative colitis (strict definition, require KELA, min 2 HDR) | 14 | 94795202 | rs9989237 | T | C | 0.2083 | NA | 0.0768 | 0.0357 | 0.03125 | finn-b-K11_UC_STRICT2 |
| Faecal incontinence | 14 | 94795202 | rs9989237 | T | C | 0.2083 | NA | -0.1101 | 0.0511 | 0.03127 | finn-b-R18_FAECAL_INCONTINENCE |
| Peptidyl-prolyl cis-trans isomerase B | 14 | 94795202 | rs9989237 | T | C | 0.21768 | 3301 | 0.0645 | 0.0301 | 0.031623 | prot-a-2340 |
| Phosphopantothenoylcysteine decarboxylase | 14 | 94795202 | rs9989237 | T | C | 0.21768 | 3301 | 0.0647 | 0.0301 | 0.031623 | prot-a-2338 |
| Semaphorin-3A | 14 | 94795202 | rs9989237 | T | C | 0.21768 | 3301 | -0.0647 | 0.0301 | 0.031623 | prot-a-2669 |
| Kelch-like protein 12 | 14 | 94795202 | rs9989237 | T | C | 0.21768 | 3301 | 0.0646 | 0.03 | 0.031623 | prot-a-1654 |
| BDNF/NT-3 growth factors receptor | 14 | 94795202 | rs9989237 | T | C | 0.21768 | 3301 | 0.0646 | 0.03 | 0.031623 | prot-a-2122 |
| RING finger protein 215 | 14 | 94795202 | rs9989237 | T | C | 0.21768 | 3301 | 0.0647 | 0.03 | 0.031623 | prot-a-2569 |
| Mammaglobin-B | 14 | 94795202 | rs9989237 | T | C | 0.21768 | 3301 | 0.0646 | 0.0301 | 0.031623 | prot-a-2648 |
| Glutathione peroxidase 7 | 14 | 94795202 | rs9989237 | T | C | 0.21768 | 3301 | 0.0647 | 0.0301 | 0.031623 | prot-a-1265 |
| NET100 0578 | 14 | 94795202 | rs9989237 | T | C | 0.21004 | 7916 | 0.0414 | 0.0192 | 0.031623 | ubm-a-1734 |
| a2009s lh S occipital ant area | 14 | 94795202 | rs9989237 | T | C | 0.21004 | 7916 | 0.038 | 0.0177 | 0.031623 | ubm-a-2805 |
| DKTatlas rh rostralmiddlefrontal area | 14 | 94795202 | rs9989237 | T | C | 0.21004 | 7916 | 0.0299 | 0.0139 | 0.031623 | ubm-a-2845 |
| Human immunodeficiency virus [HIV] disease resulting in other conditions | 14 | 94795202 | rs9989237 | T | C | 0.2083 | NA | -0.2049 | 0.0956 | 0.032 | finn-b-AB1_OTHER_CONDITIONS |
| Vitamin C | 14 | 94795202 | rs9989237 | T | C | 0.210621 | 64979 | -0.01437 | 0.006713 | 0.032 | ukb-b-19390 |
| DNA methylation PhenoAge acceleration | 14 | 94795202 | rs9989237 | T | C | 0.2005 | 6148 | 0.3393 | 0.1583 | 0.03206 | ebi-a-GCST90014298 |
| Medication for pain relief, constipation, heartburn | 14 | 94795202 | rs9989237 | T | C | 0.5804 | NA | 0.2015 | 0.09405 | 0.03212 | ukb-e-6154_p3_EAS |
| Diagnoses - main ICD10: M54 Dorsalgia | 14 | 94795202 | rs9989237 | T | C | 0.209385 | 337199 | -0.00082 | 0.000384 | 0.032157 | ukb-a-569 |
| Vitamin and mineral supplements | 14 | 94795202 | rs9989237 | T | C | 0.7681 | NA | 0.2254 | 0.1052 | 0.03221 | ukb-e-6155_p8_MID |
| Vitamin and/or mineral supplement use | 14 | 94795202 | rs9989237 | T | C | 0.6307 | NA | 0.2312 | 0.108 | 0.03231 | ukb-e-20084_p5_CSA |
| Signal-transducing adaptor protein 1 | 14 | 94795202 | rs9989237 | T | C | 0.21768 | 3301 | -0.0644 | 0.0301 | 0.032359 | prot-a-2865 |
| WSC domain-containing protein 2 | 14 | 94795202 | rs9989237 | T | C | 0.21768 | 3301 | -0.0643 | 0.03 | 0.032359 | prot-a-3235 |
| Scavenger receptor class F member 2 | 14 | 94795202 | rs9989237 | T | C | 0.21768 | 3301 | 0.0644 | 0.03 | 0.032359 | prot-a-2641 |
| Asporin | 14 | 94795202 | rs9989237 | T | C | 0.21768 | 3301 | 0.0642 | 0.0301 | 0.032359 | prot-a-192 |
| Protein CEI | 14 | 94795202 | rs9989237 | T | C | 0.21768 | 3301 | -0.0643 | 0.03 | 0.032359 | prot-a-316 |
| Interleukin-18 receptor 1 | 14 | 94795202 | rs9989237 | T | C | 0.21768 | 3301 | -0.0642 | 0.03 | 0.032359 | prot-a-1491 |
| Keratin, type II cytoskeletal 5 | 14 | 94795202 | rs9989237 | T | C | 0.21768 | 3301 | -0.0644 | 0.0301 | 0.032359 | prot-a-1685 |
| Coiled-coil domain-containing protein 90B, mitochondrial | 14 | 94795202 | rs9989237 | T | C | 0.21768 | 3301 | 0.0643 | 0.03 | 0.032359 | prot-a-385 |
| Brorin | 14 | 94795202 | rs9989237 | T | C | 0.21768 | 3301 | 0.0643 | 0.03 | 0.032359 | prot-a-3216 |
| Gremlin-1 | 14 | 94795202 | rs9989237 | T | C | 0.21768 | 3301 | 0.0644 | 0.0301 | 0.032359 | prot-a-1273 |
| Signal-regulatory protein beta-2 | 14 | 94795202 | rs9989237 | T | C | 0.21768 | 3301 | -0.0642 | 0.0301 | 0.032359 | prot-a-2734 |
| IDP dMRI TBSS L3 Fornix | 14 | 94795202 | rs9989237 | T | C | 0.21004 | 7916 | -0.0354 | 0.0165 | 0.032359 | ubm-a-441 |
| IDP T1 FAST ROIs R amygdala | 14 | 94795202 | rs9989237 | T | C | 0.21004 | 7916 | 0.0307 | 0.0144 | 0.032359 | ubm-a-133 |
| a2009s lh G insular short thickness | 14 | 94795202 | rs9989237 | T | C | 0.21004 | 7916 | -0.0393 | 0.0184 | 0.032359 | ubm-a-2976 |
| Interleukin-18 levels | 14 | 94795202 | rs9989237 | T | C | 0.218 | 21758 | 0.0268 | 0.0125 | 0.03237 | ebi-a-GCST90012024 |
| Epstein-Barr virus EA-D antibody levels | 14 | 94795202 | rs9989237 | T | C | 0.2116 | 7763 | 0.041504 | 0.019399 | 0.032394 | ebi-a-GCST90006898 |
| Thyrotropin subunit beta levels | 14 | 94795202 | rs9989237 | T | C | 0.233 | 1285 | 0.100039 | 0.046721 | 0.032608 | ebi-a-GCST90010353 |
| Gut microbiota abundance (genus Ruminococcus gnavus group id.14376) | 14 | 94795202 | rs9989237 | T | C | NA | 14306 | 0.045008 | 0.0213 | 0.032828 | ebi-a-GCST90017065 |
| Concentration of large HDL particles | 14 | 94795202 | rs9989237 | T | C | 0.20942 | NA | 0.008324 | 0.004462 | 0.033 | met-d-L_HDL_P |
| Fractured heel | 14 | 94795202 | rs9989237 | T | C | 0.209737 | 306379 | -0.00039 | 0.000182 | 0.033 | ukb-b-18389 |
| Operative procedures - main OPCS: J18.3 Total cholecystectomy NEC | 14 | 94795202 | rs9989237 | T | C | 0.209969 | 463010 | -0.0008 | 0.000376 | 0.033 | ukb-b-13803 |
| RAC-beta serine/threonine-protein kinase | 14 | 94795202 | rs9989237 | T | C | 0.21768 | 3301 | -0.0642 | 0.0301 | 0.033113 | prot-a-72 |
| Beta-defensin 123 | 14 | 94795202 | rs9989237 | T | C | 0.21768 | 3301 | 0.064 | 0.0301 | 0.033113 | prot-a-798 |
| Histatin-1 | 14 | 94795202 | rs9989237 | T | C | 0.21768 | 3301 | -0.064 | 0.0301 | 0.033113 | prot-a-1391 |
| Myeloid zinc finger 1 | 14 | 94795202 | rs9989237 | T | C | 0.21768 | 3301 | -0.0639 | 0.03 | 0.033113 | prot-a-1987 |
| Endoplasmic reticulum resident protein 29 | 14 | 94795202 | rs9989237 | T | C | 0.21768 | 3301 | 0.064 | 0.03 | 0.033113 | prot-a-984 |
| TPA-induced transmembrane protein | 14 | 94795202 | rs9989237 | T | C | 0.21768 | 3301 | 0.0639 | 0.03 | 0.033113 | prot-a-3115 |
| netmat ICA 09Aug2017 005 | 14 | 94795202 | rs9989237 | T | C | 0.21004 | 7916 | -0.0416 | 0.0195 | 0.033113 | ubm-a-3145 |
| a2009s lh S front sup area | 14 | 94795202 | rs9989237 | T | C | 0.21004 | 7916 | 0.0325 | 0.0152 | 0.033113 | ubm-a-2800 |
| NODEamps25 0010 | 14 | 94795202 | rs9989237 | T | C | 0.21004 | 7916 | 0.0399 | 0.0187 | 0.033113 | ubm-a-880 |
| NET100 0297 | 14 | 94795202 | rs9989237 | T | C | 0.21004 | 7916 | 0.0409 | 0.0192 | 0.033113 | ubm-a-1453 |
| NET100 0779 | 14 | 94795202 | rs9989237 | T | C | 0.21004 | 7916 | 0.0411 | 0.0192 | 0.033113 | ubm-a-1935 |
| Diagnoses - main ICD10: K50 Crohn's disease [regional enteritis] | 14 | 94795202 | rs9989237 | T | C | 0.209385 | 337199 | -0.0003 | 0.000139 | 0.033259 | ukb-a-552 |
| Job SOC coding: Researchers n.e.c. | 14 | 94795202 | rs9989237 | T | C | 0.208274 | 91149 | 0.001294 | 0.000608 | 0.033326 | ukb-d-22617_2329 |
| Malignant immunoproliferative diseases | 14 | 94795202 | rs9989237 | T | C | 0.2083 | NA | 0.3602 | 0.1694 | 0.03351 | finn-b-CD2_IMMUNOPROLIFERATIVE |
| Type 2 diabetes with coma | 14 | 94795202 | rs9989237 | T | C | 0.2076 | NA | -0.0828 | 0.039 | 0.03366 | finn-b-E4_DM2COMA |
| Grapefruit juice intake | 14 | 94795202 | rs9989237 | T | C | 0.6218 | NA | -0.03108 | 0.01464 | 0.03373 | ukb-e-100200_CSA |
| Interleukin enhancer-binding factor 3 | 14 | 94795202 | rs9989237 | T | C | 0.21768 | 3301 | -0.0639 | 0.0301 | 0.033884 | prot-a-1547 |
| Endothelial cell-selective adhesion molecule | 14 | 94795202 | rs9989237 | T | C | 0.21768 | 3301 | 0.0636 | 0.0301 | 0.033884 | prot-a-987 |
| Retinoid-binding protein 7 | 14 | 94795202 | rs9989237 | T | C | 0.21768 | 3301 | -0.0636 | 0.0301 | 0.033884 | prot-a-2508 |
| Peptidyl-prolyl cis-trans isomerase FKBP14 | 14 | 94795202 | rs9989237 | T | C | 0.21768 | 3301 | 0.0637 | 0.0301 | 0.033884 | prot-a-1113 |
| IDP tfMRI 90th-percentile BOLD shapes | 14 | 94795202 | rs9989237 | T | C | 0.21004 | 7916 | 0.0403 | 0.019 | 0.033884 | ubm-a-181 |
| NET25 0203 | 14 | 94795202 | rs9989237 | T | C | 0.21004 | 7916 | -0.0409 | 0.0193 | 0.033884 | ubm-a-1149 |
| NET100 1066 | 14 | 94795202 | rs9989237 | T | C | 0.21004 | 7916 | -0.04 | 0.0188 | 0.033884 | ubm-a-2222 |
| a2009s lh S parieto occipital area | 14 | 94795202 | rs9989237 | T | C | 0.21004 | 7916 | 0.0353 | 0.0167 | 0.033884 | ubm-a-2811 |
| IDP tfMRI 90th-percentile zstat shapes | 14 | 94795202 | rs9989237 | T | C | 0.21004 | 7916 | 0.0405 | 0.0191 | 0.033884 | ubm-a-183 |
| NET100 1440 | 14 | 94795202 | rs9989237 | T | C | 0.21004 | 7916 | -0.041 | 0.0193 | 0.033884 | ubm-a-2596 |
| Gut microbiota abundance (order Desulfovibrionales id.3156) | 14 | 94795202 | rs9989237 | T | C | NA | 14306 | -0.02941 | 0.013917 | 0.033906 | ebi-a-GCST90017097 |
| Clinical LDL cholesterol | 14 | 94795202 | rs9989237 | T | C | 0.20942 | NA | -0.01014 | 0.005059 | 0.034 | met-d-Clinical_LDL_C |
| Total lipids in lipoprotein particles | 14 | 94795202 | rs9989237 | T | C | 0.20942 | NA | -0.01079 | 0.005006 | 0.034 | met-d-Total_L |
| Bioavailable Testosterone | 14 | 94795202 | rs9989237 | T | C | 0.210535 | NA | 0.008459 | 0.003996 | 0.034 | ieu-b-4868 |
| Number of children ever born measurement | 14 | 94795202 | rs9989237 | T | C | NA | 60430 | -0.0174 | 0.0082 | 0.03408 | ieu-b-4828 |
| Ever had eye surgery | 14 | 94795202 | rs9989237 | T | C | 0.7902 | NA | -0.2495 | 0.1178 | 0.03419 | ukb-e-5181_AFR |
| Cervical disc disorders | 14 | 94795202 | rs9989237 | T | C | 0.2079 | NA | 0.0467 | 0.0221 | 0.0342 | finn-b-M13_CERVICDISC |
| Spondylopathies | 14 | 94795202 | rs9989237 | T | C | 0.2081 | NA | 0.0317 | 0.015 | 0.03421 | finn-b-M13_SPONDYLOPATHY |
| Non-butter spread type details: Olive oil based spread (eg: Bertolli) | 14 | 94795202 | rs9989237 | T | C | 0.208939 | 190094 | -0.00364 | 0.00172 | 0.034468 | ukb-d-2654_6 |
| Disturbances of skin sensation | 14 | 94795202 | rs9989237 | T | C | 0.2082 | NA | 0.0602 | 0.0285 | 0.03455 | finn-b-R18_DISTU_SKIN_SENSA |
| Mean platelet volume | 14 | 94795202 | rs9989237 | T | C | 0.2103 | 164454 | 0.009367 | 0.004433 | 0.0346 | ebi-a-GCST004599 |
| Red blood cell (erythrocyte) distribution width | 14 | 94795202 | rs9989237 | T | C | 0.209417 | 350473 | 0.006155 | 0.002913 | 0.034602 | ukb-d-30070_irnt |
| Job SOC coding: Information and communication technology managers | 14 | 94795202 | rs9989237 | T | C | 0.208274 | 91149 | 0.001607 | 0.000761 | 0.03461 | ukb-d-22617_1136 |
| CD16 on CD14+ CD16+ monocyte | 14 | 94795202 | rs9989237 | T | C | 0.2679 | 3617 | -0.06158 | 0.02913 | 0.03462 | ebi-a-GCST90002005 |
| Signal-regulatory protein beta-1 | 14 | 94795202 | rs9989237 | T | C | 0.21768 | 3301 | -0.0634 | 0.0301 | 0.034674 | prot-a-2733 |
| Protein FAM189A2 | 14 | 94795202 | rs9989237 | T | C | 0.21768 | 3301 | 0.0635 | 0.0301 | 0.034674 | prot-a-1038 |
| Acid-sensing ion channel 4 | 14 | 94795202 | rs9989237 | T | C | 0.21768 | 3301 | 0.0636 | 0.0301 | 0.034674 | prot-a-186 |
| Killer cell immunoglobulin-like receptor 3DL2 | 14 | 94795202 | rs9989237 | T | C | 0.21768 | 3301 | 0.0636 | 0.0301 | 0.034674 | prot-a-1646 |
| Integrin alpha-I: beta-1 complex | 14 | 94795202 | rs9989237 | T | C | 0.21768 | 3301 | -0.0636 | 0.03 | 0.034674 | prot-a-1581 |
| Homeodomain-interacting protein kinase 3 | 14 | 94795202 | rs9989237 | T | C | 0.21768 | 3301 | 0.0634 | 0.03 | 0.034674 | prot-a-1340 |
| Parathyroid hormone/parathyroid hormone-related peptide receptor | 14 | 94795202 | rs9989237 | T | C | 0.21768 | 3301 | -0.0635 | 0.0301 | 0.034674 | prot-a-2429 |
| Scavenger receptor class A member 3 | 14 | 94795202 | rs9989237 | T | C | 0.21768 | 3301 | -0.0634 | 0.0301 | 0.034674 | prot-a-2636 |
| NET100 0030 | 14 | 94795202 | rs9989237 | T | C | 0.21004 | 7916 | 0.0408 | 0.0193 | 0.034674 | ubm-a-1186 |
| NET100 1397 | 14 | 94795202 | rs9989237 | T | C | 0.21004 | 7916 | -0.041 | 0.0194 | 0.034674 | ubm-a-2553 |
| IDP T1 FAST ROIs L subcallosal cortex | 14 | 94795202 | rs9989237 | T | C | 0.21004 | 7916 | 0.0281 | 0.0133 | 0.034674 | ubm-a-78 |
| Gut microbiota abundance (family Desulfovibrionaceae id.3169) | 14 | 94795202 | rs9989237 | T | C | NA | 14306 | -0.02926 | 0.013918 | 0.034847 | ebi-a-GCST90016935 |
| TCRgd T cell %lymphocyte | 14 | 94795202 | rs9989237 | T | C | 0.2675 | 3666 | 0.06053 | 0.02868 | 0.0349 | ebi-a-GCST90001617 |
| Peptidoglycan recognition protein 1 levels | 14 | 94795202 | rs9989237 | T | C | 0.234 | 1323 | 0.09942 | 0.047059 | 0.034967 | ebi-a-GCST90010249 |
| Rheumatoid arthritis | 14 | 94795202 | rs9989237 | T | C | NA | 22515 | -0.05129 | 0.026878 | 0.035 | ieu-a-831 |
| Red cell distribution width | 14 | 94795202 | rs9989237 | T | C | 0.210038 | 408112 | 0.005495 | 0.00261 | 0.035 | ebi-a-GCST90002404 |
| Non-cancer illness code self-reported: eczema/dermatitis | 14 | 94795202 | rs9989237 | T | C | 0.209385 | 337159 | 0.001001 | 0.000475 | 0.035077 | ukb-a-99 |
| Time spent using computer | 14 | 94795202 | rs9989237 | T | C | 0.6095 | NA | 0.02986 | 0.01417 | 0.0351 | ukb-e-1080_CSA |
| Gut microbiota abundance (phylum Actinobacteria id.400) | 14 | 94795202 | rs9989237 | T | C | NA | 14306 | -0.02727 | 0.012763 | 0.035222 | ebi-a-GCST90017110 |
| Gamma glutamyltransferase | 14 | 94795202 | rs9989237 | T | C | 0.7586 | NA | 0.08138 | 0.03865 | 0.03525 | ukb-e-30730_MID |
| Interleukin-6 levels | 14 | 94795202 | rs9989237 | T | C | 0.236 | 1301 | 0.096647 | 0.045687 | 0.035321 | ebi-a-GCST90010146 |
| Pain type(s) experienced in last month | 14 | 94795202 | rs9989237 | T | C | 0.7975 | NA | 0.1038 | 0.04931 | 0.03536 | ukb-e-6159_p8_AFR |
| Rheumatoid Arthritis | 14 | 94795202 | rs9989237 | T | C | 0.39153 | NA | -0.05421 | 0.02577 | 0.035383 | bbj-a-72 |
| Interleukin-9 | 14 | 94795202 | rs9989237 | T | C | 0.21768 | 3301 | -0.0631 | 0.0301 | 0.035481 | prot-a-1546 |
| Receptor tyrosine-protein kinase erbB-2 | 14 | 94795202 | rs9989237 | T | C | 0.21768 | 3301 | 0.0631 | 0.0301 | 0.035481 | prot-a-973 |
| T-cell surface glycoprotein CD3 epsilon chain | 14 | 94795202 | rs9989237 | T | C | 0.21768 | 3301 | -0.0633 | 0.0301 | 0.035481 | prot-a-445 |
| DKTatlas rh superiorparietal area | 14 | 94795202 | rs9989237 | T | C | 0.21004 | 7916 | 0.0322 | 0.0154 | 0.035481 | ubm-a-2847 |
| NET100 1413 | 14 | 94795202 | rs9989237 | T | C | 0.21004 | 7916 | -0.0409 | 0.0195 | 0.035481 | ubm-a-2569 |
| NET100 1381 | 14 | 94795202 | rs9989237 | T | C | 0.21004 | 7916 | -0.0405 | 0.0192 | 0.035481 | ubm-a-2537 |
| Transitional B cell Absolute Count | 14 | 94795202 | rs9989237 | T | C | 0.2674 | 3656 | -0.05881 | 0.02796 | 0.03553 | ebi-a-GCST90001577 |
| Sexual dysfunction | 14 | 94795202 | rs9989237 | T | C | 0.2083 | NA | 0.289 | 0.1375 | 0.03555 | finn-b-F5_SEXDYS |
| Prolactin levels | 14 | 94795202 | rs9989237 | T | C | 0.2232 | 21758 | 0.0373 | 0.0178 | 0.03578 | ebi-a-GCST90012030 |
| Symptoms involving digestive system | 14 | 94795202 | rs9989237 | T | C | 0.6102 | NA | 0.1525 | 0.07263 | 0.03579 | ukb-e-561_CSA |
| Gut microbiota abundance (class Deltaproteobacteria id.3087) | 14 | 94795202 | rs9989237 | T | C | NA | 14306 | -0.02911 | 0.013905 | 0.035828 | ebi-a-GCST90016915 |
| Gastric ulcer | 14 | 94795202 | rs9989237 | T | C | 0.2084 | NA | -0.0699 | 0.0333 | 0.03592 | finn-b-K11_GULC |
| Carrot intake | 14 | 94795202 | rs9989237 | T | C | 0.210565 | 64949 | -0.01781 | 0.008483 | 0.036 | ukb-b-16710 |
| Trunk predicted mass | 14 | 94795202 | rs9989237 | T | C | 0.209993 | 454463 | -0.00319 | 0.001516 | 0.036 | ukb-b-9685 |
| Types of spread used on bread/crackers: Cholesterol-lowering polyunsaturated margarine on bread/crackers | 14 | 94795202 | rs9989237 | T | C | 0.210565 | 64949 | -0.00295 | 0.001408 | 0.036 | ukb-b-4946 |
| Pyogenic granuloma | 14 | 94795202 | rs9989237 | T | C | 0.2082 | NA | 0.2604 | 0.1243 | 0.03612 | finn-b-L12_PYOGENGRANUL |
| Home location - north co-ordinate (rounded) | 14 | 94795202 | rs9989237 | T | C | 0.5756 | NA | 0.05203 | 0.02485 | 0.03625 | ukb-e-22704_EAS |
| Galectin-4 | 14 | 94795202 | rs9989237 | T | C | 0.21768 | 3301 | 0.0631 | 0.0301 | 0.036308 | prot-a-1728 |
| Amyloid beta A4 precursor protein-binding family B member 3 | 14 | 94795202 | rs9989237 | T | C | 0.21768 | 3301 | -0.0631 | 0.0301 | 0.036308 | prot-a-118 |
| C-C motif chemokine 25 | 14 | 94795202 | rs9989237 | T | C | 0.21768 | 3301 | -0.063 | 0.0301 | 0.036308 | prot-a-402 |
| Secreted Ly-6/uPAR-related protein 1 | 14 | 94795202 | rs9989237 | T | C | 0.21768 | 3301 | 0.0629 | 0.0301 | 0.036308 | prot-a-2770 |
| Cold shock domain-containing protein C2 | 14 | 94795202 | rs9989237 | T | C | 0.21768 | 3301 | -0.0629 | 0.0301 | 0.036308 | prot-a-678 |
| Neurexin-3-beta | 14 | 94795202 | rs9989237 | T | C | 0.21768 | 3301 | 0.063 | 0.0301 | 0.036308 | prot-a-2106 |
| Ligand-dependent nuclear receptor corepressor-like protein | 14 | 94795202 | rs9989237 | T | C | 0.21768 | 3301 | 0.0629 | 0.0301 | 0.036308 | prot-a-1711 |
| NFU1 iron-sulfur cluster scaffold homolog, mitochondrial | 14 | 94795202 | rs9989237 | T | C | 0.21768 | 3301 | 0.063 | 0.0301 | 0.036308 | prot-a-2041 |
| NET100 1222 | 14 | 94795202 | rs9989237 | T | C | 0.21004 | 7916 | -0.0398 | 0.019 | 0.036308 | ubm-a-2378 |
| NODEamps100 0023 | 14 | 94795202 | rs9989237 | T | C | 0.21004 | 7916 | 0.0358 | 0.0171 | 0.036308 | ubm-a-914 |
| NET100 0110 | 14 | 94795202 | rs9989237 | T | C | 0.21004 | 7916 | -0.0407 | 0.0194 | 0.036308 | ubm-a-1266 |
| Achalasia of cardia | 14 | 94795202 | rs9989237 | T | C | 0.2086 | NA | 0.229 | 0.1094 | 0.03635 | finn-b-K11_ACHAL |
| Leptin levels | 14 | 94795202 | rs9989237 | T | C | 0.236 | 1301 | 0.098172 | 0.046813 | 0.036387 | ebi-a-GCST90010148 |
| Benign neoplasm of colon | 14 | 94795202 | rs9989237 | T | C | 0.612 | NA | 0.1538 | 0.07348 | 0.03639 | ukb-e-208_CSA |
| Suggestive for eosinophilic asthma | 14 | 94795202 | rs9989237 | T | C | 0.209437 | 361194 | -0.00048 | 0.00023 | 0.036446 | ukb-d-ASTHMA_EOSINOPHIL_SUGG |
| Chronic crepitant synovitis/bursitis of hand and wrist/periarhritis of wrist | 14 | 94795202 | rs9989237 | T | C | 0.2075 | NA | -0.2573 | 0.1231 | 0.03655 | finn-b-M13_CHRONSYNOVITISHANDWRIST |
| Pneumocystosis | 14 | 94795202 | rs9989237 | T | C | 0.2083 | NA | -0.3075 | 0.1471 | 0.0366 | finn-b-AB1_PNEUMOCYSTOSIS |
| Transitional B cell %lymphocyte | 14 | 94795202 | rs9989237 | T | C | 0.2674 | 3656 | -0.05884 | 0.02816 | 0.03672 | ebi-a-GCST90001578 |
| TestASV_35 (Alphaproteobacteria) prevalence | 14 | 94795202 | rs9989237 | T | C | NA | 8956 | 0.126998 | 0.060818 | 0.036783 | ebi-a-GCST90011710 |
| Diagnoses - main ICD10: K58 Irritable bowel syndrome | 14 | 94795202 | rs9989237 | T | C | 0.209437 | 361194 | 0.000342 | 0.000164 | 0.036809 | ukb-d-K58 |
| Treatment/medication code: gaviscon liquid | 14 | 94795202 | rs9989237 | T | C | 0.209974 | 462933 | 0.000387 | 0.000186 | 0.037 | ukb-b-12861 |
| Trunk fat-free mass | 14 | 94795202 | rs9989237 | T | C | 0.209991 | 454508 | -0.00316 | 0.00152 | 0.037 | ukb-b-17409 |
| Etoposide drug response (IC50) | 14 | 94795202 | rs9989237 | T | C | 0.197 | 84 | 0.434625 | 0.205087 | 0.037096 | ebi-a-GCST90011790 |
| FSC-A on HLA DR+ Natural Killer | 14 | 94795202 | rs9989237 | T | C | 0.2693 | 2971 | -0.06577 | 0.03154 | 0.03711 | ebi-a-GCST90001970 |
| Transferrin receptor protein 1 | 14 | 94795202 | rs9989237 | T | C | 0.21768 | 3301 | 0.0627 | 0.0301 | 0.037154 | prot-a-2959 |
| Importin subunit alpha-3 | 14 | 94795202 | rs9989237 | T | C | 0.21768 | 3301 | 0.0627 | 0.0301 | 0.037154 | prot-a-1678 |
| Junctophilin-3 | 14 | 94795202 | rs9989237 | T | C | 0.21768 | 3301 | 0.0627 | 0.0301 | 0.037154 | prot-a-1603 |
| Zinc finger protein 134 | 14 | 94795202 | rs9989237 | T | C | 0.21768 | 3301 | 0.0626 | 0.0301 | 0.037154 | prot-a-3260 |
| Beta-1,3-galactosyltransferase 6 | 14 | 94795202 | rs9989237 | T | C | 0.21768 | 3301 | 0.0626 | 0.0301 | 0.037154 | prot-a-209 |
| Protein dpy-30 homolog | 14 | 94795202 | rs9989237 | T | C | 0.21768 | 3301 | -0.0627 | 0.0301 | 0.037154 | prot-a-862 |
| Had other major operations | 14 | 94795202 | rs9989237 | T | C | 0.8022 | NA | 0.12 | 0.05759 | 0.03717 | ukb-e-2844_AFR |
| Childhood sunburn occasions | 14 | 94795202 | rs9989237 | T | C | 0.7931 | NA | -0.01812 | 0.008703 | 0.0374 | ukb-e-1737_AFR |
| Persons encountering health services for examination and investigation | 14 | 94795202 | rs9989237 | T | C | 0.2083 | NA | 0.017 | 0.0082 | 0.03744 | finn-b-Z21_PERSONS_ENCOUNTERI_HEALTH_SERVI_EXAM_INVES |
| 6mm weak meridian angle (left) | 14 | 94795202 | rs9989237 | T | C | 0.6115 | NA | 0.04791 | 0.02303 | 0.03748 | ukb-e-5102_CSA |
| CD20- CD38- B cell %B cell | 14 | 94795202 | rs9989237 | T | C | 0.2677 | 3659 | 0.05822 | 0.028 | 0.03763 | ebi-a-GCST90001422 |
| SHBG | 14 | 94795202 | rs9989237 | T | C | 0.7587 | NA | -0.08308 | 0.03996 | 0.03763 | ukb-e-30830_MID |
| Non-cancer illness code self-reported: type 2 diabetes | 14 | 94795202 | rs9989237 | T | C | 0.209385 | 337159 | -0.00049 | 0.000237 | 0.037782 | ukb-a-75 |
| Other and unspecified epidermal thickening | 14 | 94795202 | rs9989237 | T | C | 0.2082 | NA | 0.1966 | 0.0947 | 0.03784 | finn-b-L12_EPIDERMTHICKNAS |
| CD20- B cell %lymphocyte | 14 | 94795202 | rs9989237 | T | C | 0.2674 | 3657 | 0.05894 | 0.02838 | 0.03786 | ebi-a-GCST90001443 |
| Disorders of pituitary gland, other and/or unspecified | 14 | 94795202 | rs9989237 | T | C | 0.2084 | NA | 0.3969 | 0.1912 | 0.03788 | finn-b-E4_PITUDISNAS |
| Corneal hysteresis (left) | 14 | 94795202 | rs9989237 | T | C | 0.210776 | 97465 | -0.01126 | 0.005433 | 0.038 | ukb-b-11650 |
| Diagnoses - secondary ICD10: Z53.8 Procedure not carried out for other reasons | 14 | 94795202 | rs9989237 | T | C | 0.209969 | 463010 | 0.000806 | 0.000388 | 0.038 | ukb-b-9043 |
| Fruit consumers | 14 | 94795202 | rs9989237 | T | C | 0.6593 | NA | 0.205 | 0.09883 | 0.03801 | ukb-e-104400_CSA |
| Sodium- and chloride-dependent neutral and basic amino acid transporter B(0+) | 14 | 94795202 | rs9989237 | T | C | 0.21768 | 3301 | -0.0623 | 0.0301 | 0.038019 | prot-a-2761 |
| Ubiquitin carboxyl-terminal hydrolase 21 | 14 | 94795202 | rs9989237 | T | C | 0.21768 | 3301 | -0.0623 | 0.0301 | 0.038019 | prot-a-3179 |
| Procollagen C-endopeptidase enhancer 1 | 14 | 94795202 | rs9989237 | T | C | 0.21768 | 3301 | -0.0623 | 0.0301 | 0.038019 | prot-a-2209 |
| Protein p13 MTCP-1 | 14 | 94795202 | rs9989237 | T | C | 0.21768 | 3301 | -0.0623 | 0.0301 | 0.038019 | prot-a-1960 |
| IDP dMRI ProbtrackX MO ilf r | 14 | 94795202 | rs9989237 | T | C | 0.21004 | 7916 | -0.0384 | 0.0186 | 0.038019 | ubm-a-697 |
| O_Selenomonadales prevalence | 14 | 94795202 | rs9989237 | T | C | NA | 8956 | -0.11116 | 0.053583 | 0.038025 | ebi-a-GCST90011554 |
| C_Negativicutes prevalence | 14 | 94795202 | rs9989237 | T | C | NA | 8956 | -0.11116 | 0.053583 | 0.038025 | ebi-a-GCST90011534 |
| Mean corpuscular hemoglobin concentration | 14 | 94795202 | rs9989237 | T | C | 0.2106 | 172851 | -0.00879 | 0.004238 | 0.0381 | ebi-a-GCST004605 |
| Haemorrhoidal disease | 14 | 94795202 | rs9989237 | T | C | 0.2112 | 944133 | 0.01 | 0.0048 | 0.03828 | ebi-a-GCST90014033 |
| Habit and impulse disorders | 14 | 94795202 | rs9989237 | T | C | 0.2083 | NA | -0.2246 | 0.1085 | 0.03854 | finn-b-F5_HABIT |
| OTU99_39 (Alphaproteobacteria) prevalence | 14 | 94795202 | rs9989237 | T | C | NA | 8956 | 0.119249 | 0.057638 | 0.038551 | ebi-a-GCST90011657 |
| Malignant immunoproliferative diseases (all cancers excluded) | 14 | 94795202 | rs9989237 | T | C | 0.2081 | NA | 0.337 | 0.163 | 0.03873 | finn-b-CD2_IMMUNOPROLIFERATIVE_EXALLC |
| COVID-19 (very severe respiratory confirmed vs population) RELEASE 5 | 14 | 94795202 | rs9989237 | T | C | 0.2102 | 707407 | 0.07489 | 0.03624 | 0.03878 | ebi-a-GCST011076 |
| 6mm cylindrical power angle (left) | 14 | 94795202 | rs9989237 | T | C | 0.6115 | NA | 0.04762 | 0.02305 | 0.03885 | ukb-e-5113_CSA |
| Galectin-3 levels | 14 | 94795202 | rs9989237 | T | C | 0.234 | 1323 | 0.097126 | 0.046937 | 0.038854 | ebi-a-GCST90010217 |
| Cystatin-8 | 14 | 94795202 | rs9989237 | T | C | 0.21768 | 3301 | -0.0622 | 0.0301 | 0.038905 | prot-a-706 |
| Intercellular adhesion molecule 3 | 14 | 94795202 | rs9989237 | T | C | 0.21768 | 3301 | 0.0621 | 0.0301 | 0.038905 | prot-a-1399 |
| Succinate dehydrogenase assembly factor 2, mitochondrial | 14 | 94795202 | rs9989237 | T | C | 0.21768 | 3301 | -0.0622 | 0.03 | 0.038905 | prot-a-2657 |
| Integral membrane protein 2A | 14 | 94795202 | rs9989237 | T | C | 0.21768 | 3301 | -0.0621 | 0.0301 | 0.038905 | prot-a-1589 |
| 60S ribosomal protein L30 | 14 | 94795202 | rs9989237 | T | C | 0.21768 | 3301 | -0.0622 | 0.0301 | 0.038905 | prot-a-2586 |
| Pentraxin-related protein PTX3 | 14 | 94795202 | rs9989237 | T | C | 0.21768 | 3301 | -0.0619 | 0.0301 | 0.038905 | prot-a-2451 |
| Ras-related protein Rab-35 | 14 | 94795202 | rs9989237 | T | C | 0.21768 | 3301 | -0.0621 | 0.03 | 0.038905 | prot-a-2476 |
| Membrane-associated progesterone receptor component 2 | 14 | 94795202 | rs9989237 | T | C | 0.21768 | 3301 | 0.0621 | 0.0301 | 0.038905 | prot-a-2260 |
| Suppressor of cytokine signaling 3 | 14 | 94795202 | rs9989237 | T | C | 0.21768 | 3301 | -0.062 | 0.0301 | 0.038905 | prot-a-2797 |
| Appetite-regulating hormone | 14 | 94795202 | rs9989237 | T | C | 0.21768 | 3301 | 0.062 | 0.03 | 0.038905 | prot-a-1212 |
| Congenital deformities of hip | 14 | 94795202 | rs9989237 | T | C | 0.2083 | NA | 0.2497 | 0.1209 | 0.03892 | finn-b-Q17_CONGEN_DEFORMITI_HIP |
| Hematocrit | 14 | 94795202 | rs9989237 | T | C | 0.210511 | 562259 | 0.004879 | 0.002363 | 0.038951 | ebi-a-GCST90002304 |
| Phospholipids to total lipids ratio in IDL | 14 | 94795202 | rs9989237 | T | C | 0.20942 | NA | 0.009655 | 0.005107 | 0.039 | met-d-IDL_PL_pct |
| Types of transport used (excluding work): Walk | 14 | 94795202 | rs9989237 | T | C | 0.209943 | 460491 | -0.00264 | 0.001276 | 0.039 | ukb-b-14926 |
| Pain type(s) experienced in last month: Neck or shoulder pain | 14 | 94795202 | rs9989237 | T | C | 0.209979 | 461857 | -0.00221 | 0.001074 | 0.039 | ukb-b-18596 |
| 6mm weak meridian (left) | 14 | 94795202 | rs9989237 | T | C | 0.211254 | 83410 | 0.011592 | 0.005624 | 0.039 | ukb-b-13538 |
| Illnesses of mother: Alzheimer's disease/dementia | 14 | 94795202 | rs9989237 | T | C | 0.209385 | 308780 | -0.00178 | 0.000864 | 0.03917 | ukb-a-210 |
| Operative procedures - main OPCS4 | 14 | 94795202 | rs9989237 | T | C | 0.7908 | NA | -0.164 | 0.07959 | 0.03929 | ukb-e-41200_p346_AFR |
| Milk type used: Other type of milk | 14 | 94795202 | rs9989237 | T | C | 0.209426 | 360806 | 0.00064 | 0.000311 | 0.039557 | ukb-d-1418_5 |
| Ulcerative colitis (strict definition, require KELA) | 14 | 94795202 | rs9989237 | T | C | 0.2083 | NA | 0.0718 | 0.0349 | 0.03956 | finn-b-K11_UC_STRICT |
| Adhesive capsulitis of shoulder | 14 | 94795202 | rs9989237 | T | C | 0.2077 | NA | 0.0682 | 0.0332 | 0.03961 | finn-b-M13_ADHCAPSULITIS |
| Tinnitus: Yes, now a lot of the time | 14 | 94795202 | rs9989237 | T | C | 0.210001 | 117882 | -0.00163 | 0.00079 | 0.039652 | ukb-d-4803_12 |
| COVID-19 (hospitalized vs population) RELEASE 5 | 14 | 94795202 | rs9989237 | T | C | 0.2075 | 1557411 | 0.062609 | 0.030445 | 0.03974 | ebi-a-GCST011083 |
| Stromelysin-2 | 14 | 94795202 | rs9989237 | T | C | 0.21768 | 3301 | 0.0618 | 0.0301 | 0.039811 | prot-a-1910 |
| C-C motif chemokine 19 | 14 | 94795202 | rs9989237 | T | C | 0.21768 | 3301 | 0.0618 | 0.0301 | 0.039811 | prot-a-395 |
| MAM domain-containing protein 2 | 14 | 94795202 | rs9989237 | T | C | 0.21768 | 3301 | 0.0619 | 0.0301 | 0.039811 | prot-a-1831 |
| MAP kinase-activated protein kinase 3 | 14 | 94795202 | rs9989237 | T | C | 0.21768 | 3301 | 0.0617 | 0.0301 | 0.039811 | prot-a-1852 |
| Calcium uptake protein 3, mitochondrial | 14 | 94795202 | rs9989237 | T | C | 0.21768 | 3301 | 0.0618 | 0.0301 | 0.039811 | prot-a-896 |
| NET100 0201 | 14 | 94795202 | rs9989237 | T | C | 0.21004 | 7916 | 0.0395 | 0.0192 | 0.039811 | ubm-a-1357 |
| Victim of sexual assault | 14 | 94795202 | rs9989237 | T | C | 0.20995 | 116671 | -0.00363 | 0.001767 | 0.039859 | ukb-d-20531 |
| Recent inability to stop or control worrying | 14 | 94795202 | rs9989237 | T | C | 0.6148 | NA | -0.06359 | 0.03096 | 0.03999 | ukb-e-20509_CSA |
| Concentration of very small VLDL particles | 14 | 94795202 | rs9989237 | T | C | 0.20942 | NA | -0.01012 | 0.005021 | 0.04 | met-d-XS_VLDL_P |
| Sphingomyelins | 14 | 94795202 | rs9989237 | T | C | 0.209439 | NA | -0.01023 | 0.004758 | 0.04 | met-d-Sphingomyelins |
| Percentage of invited food questionnaires completed | 14 | 94795202 | rs9989237 | T | C | 0.209784 | 300639 | 0.224478 | 0.109992 | 0.04 | ebi-a-GCST90012790 |
| Diagnoses - secondary ICD10: R63.4 Abnormal weight loss | 14 | 94795202 | rs9989237 | T | C | 0.209969 | 463010 | -0.0004 | 0.000196 | 0.04 | ukb-b-13845 |
| Treatment/medication code: simvastatin | 14 | 94795202 | rs9989237 | T | C | 0.209974 | 462933 | -0.00165 | 0.000803 | 0.04 | ukb-b-11268 |
| PCT where patients GP was registered: HAMPSHIRE PCT | 14 | 94795202 | rs9989237 | T | C | 0.209969 | 463010 | -0.00038 | 0.000185 | 0.04 | ukb-b-14262 |
| Other hearing loss | 14 | 94795202 | rs9989237 | T | C | 0.2083 | NA | 0.0574 | 0.0279 | 0.0401 | finn-b-H8_OTHHEARINGLOSS |
| Maternal history of Alzheimer's disease | 14 | 94795202 | rs9989237 | T | C | NA | 288676 | -0.02575 | 0.01255 | 0.040188 | ebi-a-GCST005923 |
| Flatulence and related conditions | 14 | 94795202 | rs9989237 | T | C | 0.2084 | NA | -0.3383 | 0.165 | 0.040301 | finn-b-R18_FLATU_RELATED_CONDI |
| Gut microbiota abundance (genus Family XIII AD3011 group id.11293) | 14 | 94795202 | rs9989237 | T | C | NA | 14306 | -0.02944 | 0.01429 | 0.040379 | ebi-a-GCST90017008 |
| Direct bilirubin | 14 | 94795202 | rs9989237 | T | C | 0.20949 | 292933 | -0.00628 | 0.00307 | 0.04066 | ukb-d-30660_irnt |
| Interleukin-17A | 14 | 94795202 | rs9989237 | T | C | 0.21768 | 3301 | 0.0616 | 0.0301 | 0.040738 | prot-a-1482 |
| E3 ubiquitin-protein ligase RNF13 | 14 | 94795202 | rs9989237 | T | C | 0.21768 | 3301 | -0.0615 | 0.0301 | 0.040738 | prot-a-2563 |
| Anaphase-promoting complex subunit 10 | 14 | 94795202 | rs9989237 | T | C | 0.21768 | 3301 | 0.0614 | 0.0301 | 0.040738 | prot-a-90 |
| NET100 0574 | 14 | 94795202 | rs9989237 | T | C | 0.21004 | 7916 | 0.0395 | 0.0193 | 0.040738 | ubm-a-1730 |
| Pneumonia due to other infectious organisms, not elsewhere classified | 14 | 94795202 | rs9989237 | T | C | 0.208 | NA | -0.1859 | 0.0909 | 0.04082 | finn-b-J10_PNEUMONONBACT |
| Actinic keratosis | 14 | 94795202 | rs9989237 | T | C | 0.2084 | NA | -0.0552 | 0.027 | 0.0409 | finn-b-L12_ACTINKERA |
| Gut microbiota abundance (genus Dialister id.2183) | 14 | 94795202 | rs9989237 | T | C | NA | 14306 | 0.032129 | 0.015564 | 0.040943 | ebi-a-GCST90016988 |
| Main speciality of consultant (recoded): Plastic surgery | 14 | 94795202 | rs9989237 | T | C | 0.209978 | 461218 | -0.00104 | 0.000508 | 0.041 | ukb-b-8938 |
| Diagnoses - secondary ICD10: Z88.6 Personal history of allergy to analgesic agent | 14 | 94795202 | rs9989237 | T | C | 0.209969 | 463010 | -0.0004 | 0.000194 | 0.041 | ukb-b-4267 |
| TestASV_28 (Holdemanella) abundance | 14 | 94795202 | rs9989237 | T | C | NA | 8956 | 0.0986 | 0.048282 | 0.041134 | ebi-a-GCST90011505 |
| TNF-related apoptosis-inducing ligand levels | 14 | 94795202 | rs9989237 | T | C | 0.2206 | 21758 | -0.028 | 0.0137 | 0.04122 | ebi-a-GCST90012011 |
| Tea intake | 14 | 94795202 | rs9989237 | T | C | 0.793 | NA | 0.03813 | 0.01868 | 0.04122 | ukb-e-1488_AFR |
| D12 Benign neoplasm of colon, rectum, anus and anal canal | 14 | 94795202 | rs9989237 | T | C | 0.6114 | NA | 0.1964 | 0.09624 | 0.04128 | ukb-e-D12_CSA |
| NA | 14 | 94795202 | rs9989237 | T | C | 0.6112 | NA | 0.1445 | 0.07084 | 0.04135 | ukb-e-recode254_CSA |
| NA | 14 | 94795202 | rs9989237 | T | C | 0.6112 | NA | 0.1445 | 0.07084 | 0.04135 | ukb-e-recode123_CSA |
| Uncharacterized protein C17orf78 | 14 | 94795202 | rs9989237 | T | C | 0.21768 | 3301 | 0.0612 | 0.03 | 0.041687 | prot-a-287 |
| Serine protease inhibitor Kazal-type 6 | 14 | 94795202 | rs9989237 | T | C | 0.21768 | 3301 | -0.0613 | 0.0301 | 0.041687 | prot-a-2820 |
| Rho guanine nucleotide exchange factor 7 | 14 | 94795202 | rs9989237 | T | C | 0.21768 | 3301 | -0.0611 | 0.0301 | 0.041687 | prot-a-158 |
| Cathepsin G | 14 | 94795202 | rs9989237 | T | C | 0.21768 | 3301 | 0.0613 | 0.0301 | 0.041687 | prot-a-723 |
| Probable ATP-dependent RNA helicase DDX58 | 14 | 94795202 | rs9989237 | T | C | 0.21768 | 3301 | -0.0613 | 0.0301 | 0.041687 | prot-a-779 |
| RING finger protein 122 | 14 | 94795202 | rs9989237 | T | C | 0.21768 | 3301 | -0.0611 | 0.0301 | 0.041687 | prot-a-2561 |
| Cell surface glycoprotein CD200 receptor 1 | 14 | 94795202 | rs9989237 | T | C | 0.21768 | 3301 | 0.0613 | 0.03 | 0.041687 | prot-a-423 |
| NET25 0016 | 14 | 94795202 | rs9989237 | T | C | 0.21004 | 7916 | -0.0391 | 0.0192 | 0.041687 | ubm-a-962 |
| NET25 0056 | 14 | 94795202 | rs9989237 | T | C | 0.21004 | 7916 | 0.0392 | 0.0193 | 0.041687 | ubm-a-1002 |
| NET100 0459 | 14 | 94795202 | rs9989237 | T | C | 0.21004 | 7916 | 0.0394 | 0.0193 | 0.041687 | ubm-a-1615 |
| NET100 0466 | 14 | 94795202 | rs9989237 | T | C | 0.21004 | 7916 | -0.0389 | 0.0191 | 0.041687 | ubm-a-1622 |
| NODEamps25 0004 | 14 | 94795202 | rs9989237 | T | C | 0.21004 | 7916 | 0.0378 | 0.0185 | 0.041687 | ubm-a-874 |
| NET100 0332 | 14 | 94795202 | rs9989237 | T | C | 0.21004 | 7916 | 0.0394 | 0.0193 | 0.041687 | ubm-a-1488 |
| C-C motif chemokine 3 levels | 14 | 94795202 | rs9989237 | T | C | 0.218 | 21758 | 0.0254 | 0.0125 | 0.0418 | ebi-a-GCST90012055 |
| Anoxic brain damage | 14 | 94795202 | rs9989237 | T | C | 0.2082 | NA | -0.2592 | 0.1273 | 0.04183 | finn-b-G6_ANOXBRAINDAM |
| Cholesteryl esters in chylomicrons and extremely large VLDL | 14 | 94795202 | rs9989237 | T | C | 0.20942 | NA | -0.00913 | 0.004918 | 0.042 | met-d-XXL_VLDL_CE |
| CD24 on IgD- CD38- B cell | 14 | 94795202 | rs9989237 | T | C | 0.2674 | 3648 | -0.05937 | 0.02918 | 0.042 | ebi-a-GCST90001769 |
| Sex hormone-binding globulin levels | 14 | 94795202 | rs9989237 | T | C | 0.210034 | 189473 | -0.00415 | 0.001744 | 0.042 | ebi-a-GCST90012107 |
| Duration to complete alphanumeric path (trail #2) | 14 | 94795202 | rs9989237 | T | C | 0.210126 | 99477 | -0.0111 | 0.005452 | 0.042 | ukb-b-20140 |
| Arm fat-free mass (right) | 14 | 94795202 | rs9989237 | T | C | 0.20999 | 454753 | -0.00309 | 0.001518 | 0.042 | ukb-b-19520 |
| Month of birth | 14 | 94795202 | rs9989237 | T | C | 0.791 | NA | -0.1625 | 0.07991 | 0.04202 | ukb-e-52_p8_AFR |
| Interleukin-1 receptor-like 2 levels | 14 | 94795202 | rs9989237 | T | C | 0.236 | 1301 | 0.095327 | 0.046836 | 0.042172 | ebi-a-GCST90010143 |
| Symptoms and signs involving the urinary system | 14 | 94795202 | rs9989237 | T | C | 0.2083 | NA | 0.0302 | 0.0149 | 0.04259 | finn-b-R18_SYMPTOMS_SIGNS_INVOLVI_URINARY_SYSTEM |
| IgD+ CD38+ B cell %lymphocyte | 14 | 94795202 | rs9989237 | T | C | 0.2674 | 3656 | -0.05733 | 0.02827 | 0.042631 | ebi-a-GCST90001429 |
| Noggin | 14 | 94795202 | rs9989237 | T | C | 0.21768 | 3301 | 0.0609 | 0.0301 | 0.042658 | prot-a-2064 |
| Interferon regulatory factor 1 | 14 | 94795202 | rs9989237 | T | C | 0.21768 | 3301 | -0.0609 | 0.0301 | 0.042658 | prot-a-1566 |
| Fibroblast growth factor 9 | 14 | 94795202 | rs9989237 | T | C | 0.21768 | 3301 | 0.061 | 0.0301 | 0.042658 | prot-a-1101 |
| C-C motif chemokine 16 | 14 | 94795202 | rs9989237 | T | C | 0.21768 | 3301 | -0.061 | 0.0301 | 0.042658 | prot-a-393 |
| F-box/LRR-repeat protein 5 | 14 | 94795202 | rs9989237 | T | C | 0.21768 | 3301 | 0.061 | 0.0301 | 0.042658 | prot-a-1068 |
| Insulin-induced gene 1 protein | 14 | 94795202 | rs9989237 | T | C | 0.21768 | 3301 | 0.061 | 0.0301 | 0.042658 | prot-a-1559 |
| Ethanolamine kinase 1 | 14 | 94795202 | rs9989237 | T | C | 0.21768 | 3301 | 0.0608 | 0.0301 | 0.042658 | prot-a-993 |
| Transcription regulator protein BACH2 | 14 | 94795202 | rs9989237 | T | C | 0.21768 | 3301 | -0.0608 | 0.0301 | 0.042658 | prot-a-223 |
| N-acetyllactosaminide beta-1,6-N-acetylglucosaminyl-transferase, isoform C | 14 | 94795202 | rs9989237 | T | C | 0.21768 | 3301 | 0.0608 | 0.03 | 0.042658 | prot-a-1186 |
| IDP T1 FAST ROIs R cerebellum VIIIb | 14 | 94795202 | rs9989237 | T | C | 0.21004 | 7916 | 0.0311 | 0.0154 | 0.042658 | ubm-a-158 |
| IDP dMRI TBSS FA Fornix | 14 | 94795202 | rs9989237 | T | C | 0.21004 | 7916 | 0.0341 | 0.0168 | 0.042658 | ubm-a-201 |
| IDP tfMRI median zstat faces | 14 | 94795202 | rs9989237 | T | C | 0.21004 | 7916 | 0.0387 | 0.0191 | 0.042658 | ubm-a-186 |
| IgD+ CD38+ B cell Absolute Count | 14 | 94795202 | rs9989237 | T | C | 0.2674 | 3656 | -0.05633 | 0.0278 | 0.04283 | ebi-a-GCST90001392 |
| CD8dim Natural Killer T %lymphocyte | 14 | 94795202 | rs9989237 | T | C | 0.2674 | 3668 | 0.05758 | 0.02843 | 0.04291 | ebi-a-GCST90001635 |
| Triglycerides in IDL | 14 | 94795202 | rs9989237 | T | C | 0.20942 | NA | -0.00963 | 0.00503 | 0.043 | met-d-IDL_TG |
| Why reduced smoking: Illness or ill health | 14 | 94795202 | rs9989237 | T | C | 0.211255 | 14951 | -0.00994 | 0.004908 | 0.043 | ukb-b-9930 |
| Lymphocyte percentage | 14 | 94795202 | rs9989237 | T | C | 0.7582 | NA | -0.08651 | 0.04275 | 0.043 | ukb-e-30180_MID |
| Neurological diseases | 14 | 94795202 | rs9989237 | T | C | 0.2083 | NA | 0.0177 | 0.0088 | 0.04303 | finn-b-G6_NEURO |
| Diastolic blood pressure, combined automated + manual reading, adjusted by medication | 14 | 94795202 | rs9989237 | T | C | 0.575 | NA | -0.05861 | 0.029 | 0.04324 | ukb-e-DBP_p3_EAS |
| Type of fat/oil used in cooking: Low fat olive spread for | 14 | 94795202 | rs9989237 | T | C | 0.209871 | 51427 | -0.00229 | 0.001131 | 0.043298 | ukb-d-20090_367 |
| pheno 48 / pheno 49 | 14 | 94795202 | rs9989237 | T | C | 0.5758 | NA | -0.03938 | 0.01949 | 0.04332 | ukb-e-whr_EAS |
| Mean arterial pressure, combined automated + manual reading, adjusted by medication | 14 | 94795202 | rs9989237 | T | C | 0.575 | NA | -0.05775 | 0.02859 | 0.04338 | ukb-e-MAP_p4_EAS |
| Other specified/unspecified systemic involvement of connective tissue | 14 | 94795202 | rs9989237 | T | C | 0.2083 | NA | 0.1243 | 0.0615 | 0.04343 | finn-b-M13_CONNECTNAS |
| Secretogranin-2 | 14 | 94795202 | rs9989237 | T | C | 0.21768 | 3301 | 0.0606 | 0.0301 | 0.043652 | prot-a-2644 |
| Calcium-dependent phospholipase A2 | 14 | 94795202 | rs9989237 | T | C | 0.21768 | 3301 | 0.0606 | 0.0301 | 0.043652 | prot-a-2290 |
| NET100 0396 | 14 | 94795202 | rs9989237 | T | C | 0.21004 | 7916 | -0.0385 | 0.0191 | 0.043652 | ubm-a-1552 |
| IDP dMRI TBSS L2 Fornix | 14 | 94795202 | rs9989237 | T | C | 0.21004 | 7916 | -0.0333 | 0.0165 | 0.043652 | ubm-a-393 |
| NET100 0279 | 14 | 94795202 | rs9989237 | T | C | 0.21004 | 7916 | 0.0384 | 0.0191 | 0.043652 | ubm-a-1435 |
| NET100 1357 | 14 | 94795202 | rs9989237 | T | C | 0.21004 | 7916 | 0.0391 | 0.0193 | 0.043652 | ubm-a-2513 |
| NA | 14 | 94795202 | rs9989237 | T | C | 0.6112 | NA | 0.1452 | 0.072 | 0.04372 | ukb-e-recode234_CSA |
| Spinal enthesopathy | 14 | 94795202 | rs9989237 | T | C | 0.2076 | NA | 0.1558 | 0.0772 | 0.04377 | finn-b-M13_SPINENTHES |
| Father's age at death | 14 | 94795202 | rs9989237 | T | C | 0.209385 | 248726 | -0.00695 | 0.003451 | 0.043864 | ukb-a-41 |
| Central corneal thickness | 14 | 94795202 | rs9989237 | T | C | 0.219 | 17803 | -0.9173 | 0.4552 | 0.0439 | ebi-a-GCST006366 |
| interleukin 6 receptor | 14 | 94795202 | rs9989237 | T | C | 0.2384 | 3394 | -0.0633 | 0.0314 | 0.04396 | prot-b-23 |
| Work hours - lumped category: 15 to less-than-20 hours | 14 | 94795202 | rs9989237 | T | C | 0.208421 | 75177 | 0.004254 | 0.002112 | 0.043988 | ukb-d-22604_1 |
| Total fatty acids | 14 | 94795202 | rs9989237 | T | C | 0.209439 | NA | -0.0107 | 0.005049 | 0.044 | met-d-Total_FA |
| Bipolar disorder vs anorexia nervosa (ordinary least squares (OLS)) | 14 | 94795202 | rs9989237 | T | C | NA | 37344 | -0.0074 | 0.0037 | 0.044 | ebi-a-GCST90016605 |
| Qualifications: O levels/GCSEs or equivalent | 14 | 94795202 | rs9989237 | T | C | 0.209977 | 458079 | -0.00257 | 0.001271 | 0.044 | ukb-b-18099 |
| Speech-reception-threshold (SRT) estimate (left) | 14 | 94795202 | rs9989237 | T | C | 0.210633 | 146131 | -0.00906 | 0.004489 | 0.044 | ukb-b-9052 |
| Pizza intake | 14 | 94795202 | rs9989237 | T | C | 0.210565 | 64949 | -0.00844 | 0.004182 | 0.044 | ukb-b-3852 |
| Enthesopathies of lower limb, excluding foot | 14 | 94795202 | rs9989237 | T | C | 0.2078 | NA | 0.0611 | 0.0303 | 0.04406 | finn-b-M13_ENTESOPATHYLOW |
| Final attempt correct | 14 | 94795202 | rs9989237 | T | C | 0.6056 | NA | -0.1119 | 0.05559 | 0.044121 | ukb-e-4294_p1_CSA |
| Other symptoms and signs involving cognitive functions and awareness | 14 | 94795202 | rs9989237 | T | C | 0.2083 | NA | 0.0433 | 0.0215 | 0.04421 | finn-b-R18_OTHER_SYMPTOMS_SIGNS_INVOLVI_COGNITIVE_FUNCTIONS_AWARE |
| Standard tea intake | 14 | 94795202 | rs9989237 | T | C | 0.7781 | NA | 0.1395 | 0.06937 | 0.04425 | ukb-e-100400_AFR |
| Anxiety disorders | 14 | 94795202 | rs9989237 | T | C | 0.2083 | NA | 0.0277 | 0.0138 | 0.04426 | finn-b-KRA_PSY_ANXIETY |
| Red blood cell (erythrocyte) distribution width | 14 | 94795202 | rs9989237 | T | C | 0.5768 | NA | 0.06398 | 0.03183 | 0.04445 | ukb-e-30070_EAS |
| Chronic gastritis | 14 | 94795202 | rs9989237 | T | C | 0.209437 | 361194 | 0.000408 | 0.000203 | 0.044484 | ukb-d-K11_CHRONGASTR |
| T cell Absolute Count | 14 | 94795202 | rs9989237 | T | C | 0.2672 | 3653 | 0.05654 | 0.02813 | 0.0445 | ebi-a-GCST90001603 |
| Coxsackievirus and adenovirus receptor | 14 | 94795202 | rs9989237 | T | C | 0.21768 | 3301 | -0.0604 | 0.0301 | 0.044668 | prot-a-737 |
| EF-hand calcium-binding domain-containing protein 14 | 14 | 94795202 | rs9989237 | T | C | 0.21768 | 3301 | 0.0603 | 0.0301 | 0.044668 | prot-a-895 |
| Carbohydrate sulfotransferase 9 | 14 | 94795202 | rs9989237 | T | C | 0.21768 | 3301 | -0.0604 | 0.03 | 0.044668 | prot-a-559 |
| Tensin-2 | 14 | 94795202 | rs9989237 | T | C | 0.21768 | 3301 | 0.0604 | 0.0301 | 0.044668 | prot-a-3069 |
| Kin of IRRE-like protein 2 | 14 | 94795202 | rs9989237 | T | C | 0.21768 | 3301 | 0.0604 | 0.0301 | 0.044668 | prot-a-1649 |
| UPF0577 protein KIAA1324-like | 14 | 94795202 | rs9989237 | T | C | 0.21768 | 3301 | -0.0605 | 0.0301 | 0.044668 | prot-a-1632 |
| CUB and zona pellucida-like domain-containing protein 1 | 14 | 94795202 | rs9989237 | T | C | 0.21768 | 3301 | -0.0602 | 0.03 | 0.044668 | prot-a-735 |
| Granulins | 14 | 94795202 | rs9989237 | T | C | 0.21768 | 3301 | -0.0604 | 0.03 | 0.044668 | prot-a-1277 |
| Beta-defensin 134 | 14 | 94795202 | rs9989237 | T | C | 0.21768 | 3301 | -0.0604 | 0.03 | 0.044668 | prot-a-801 |
| IDP T1 FAST ROIs R latocc cortex inf | 14 | 94795202 | rs9989237 | T | C | 0.21004 | 7916 | 0.0314 | 0.0156 | 0.044668 | ubm-a-71 |
| IDP tfMRI 90th-percentile zstat faces | 14 | 94795202 | rs9989237 | T | C | 0.21004 | 7916 | 0.0379 | 0.0189 | 0.044668 | ubm-a-187 |
| IDP dMRI TBSS MD Cerebral peduncle L | 14 | 94795202 | rs9989237 | T | C | 0.21004 | 7916 | 0.0339 | 0.0168 | 0.044668 | ubm-a-259 |
| a2009s rh G&S cingul-Ant area | 14 | 94795202 | rs9989237 | T | C | 0.21004 | 7916 | 0.0262 | 0.0131 | 0.044668 | ubm-a-2858 |
| DKTatlas rh precentral area | 14 | 94795202 | rs9989237 | T | C | 0.21004 | 7916 | 0.0276 | 0.0138 | 0.044668 | ubm-a-2842 |
| NET100 1485 | 14 | 94795202 | rs9989237 | T | C | 0.21004 | 7916 | -0.0392 | 0.0195 | 0.044668 | ubm-a-2641 |
| Other disorders of male genital organs | 14 | 94795202 | rs9989237 | T | C | 0.2082 | NA | 0.1634 | 0.0814 | 0.04471 | finn-b-N14_OTHDISMALEGEN |
| CD4-CD8- T cell Absolute Count | 14 | 94795202 | rs9989237 | T | C | 0.2671 | 3652 | 0.05429 | 0.02704 | 0.04475 | ebi-a-GCST90001598 |
| Calcium | 14 | 94795202 | rs9989237 | T | C | 0.6217 | NA | 0.09202 | 0.04587 | 0.04487 | ukb-e-100024_CSA |
| Non-accidental death in close genetic family | 14 | 94795202 | rs9989237 | T | C | 0.6036 | NA | -0.09219 | 0.04597 | 0.0449 | ukb-e-4501_CSA |
| Time to complete round | 14 | 94795202 | rs9989237 | T | C | 0.209841 | 110323 | -0.01038 | 0.005183 | 0.045 | ukb-b-10412 |
| Diagnoses - main ICD10: K20 Oesophagitis | 14 | 94795202 | rs9989237 | T | C | 0.209385 | 337199 | 0.000596 | 0.000298 | 0.045079 | ukb-a-544 |
| CD8 on CD28- CD8+ T cell | 14 | 94795202 | rs9989237 | T | C | 0.2699 | 2920 | 0.06376 | 0.03181 | 0.04508 | ebi-a-GCST90002120 |
| Belittlement by partner or ex-partner as an adult | 14 | 94795202 | rs9989237 | T | C | 0.6151 | NA | -0.08022 | 0.04004 | 0.04511 | ukb-e-20521_CSA |
| Psoriatic arthropathies | 14 | 94795202 | rs9989237 | T | C | 0.2078 | NA | -0.0918 | 0.0458 | 0.04525 | finn-b-M13_PSORIARTH |
| Diseases of liver | 14 | 94795202 | rs9989237 | T | C | 0.2083 | NA | 0.0498 | 0.0249 | 0.0453 | finn-b-K11_LIVER |
| Time spent using computer | 14 | 94795202 | rs9989237 | T | C | 0.5786 | NA | 0.04882 | 0.02441 | 0.04549 | ukb-e-1080_EAS |
| Folate | 14 | 94795202 | rs9989237 | T | C | 0.6217 | NA | 0.09047 | 0.04526 | 0.045621 | ukb-e-100014_CSA |
| Protein O-glucosyltransferase 1 | 14 | 94795202 | rs9989237 | T | C | 0.21768 | 3301 | 0.0601 | 0.0301 | 0.045709 | prot-a-2320 |
| Carbohydrate sulfotransferase 2 | 14 | 94795202 | rs9989237 | T | C | 0.21768 | 3301 | 0.0601 | 0.0301 | 0.045709 | prot-a-555 |
| NET100 0989 | 14 | 94795202 | rs9989237 | T | C | 0.21004 | 7916 | 0.0386 | 0.0193 | 0.045709 | ubm-a-2145 |
| NET100 0524 | 14 | 94795202 | rs9989237 | T | C | 0.21004 | 7916 | -0.0386 | 0.0194 | 0.045709 | ubm-a-1680 |
| NET100 1050 | 14 | 94795202 | rs9989237 | T | C | 0.21004 | 7916 | -0.0383 | 0.0192 | 0.045709 | ubm-a-2206 |
| a2009s rh Lat Fis-ant-Vertical area | 14 | 94795202 | rs9989237 | T | C | 0.21004 | 7916 | 0.0369 | 0.0185 | 0.045709 | ubm-a-2892 |
| IDP tfMRI median zstat shapes | 14 | 94795202 | rs9989237 | T | C | 0.21004 | 7916 | 0.0383 | 0.0191 | 0.045709 | ubm-a-182 |
| Main speciality of consultant (recoded) | 14 | 94795202 | rs9989237 | T | C | 0.7942 | NA | 0.2178 | 0.109 | 0.04572 | ukb-e-41245_p41_AFR |
| C-X-C motif chemokine 6 levels | 14 | 94795202 | rs9989237 | T | C | 0.2198 | 21758 | 0.0266 | 0.0133 | 0.04583 | ebi-a-GCST90012057 |
| Age completed full time education | 14 | 94795202 | rs9989237 | T | C | 0.5687 | NA | -0.05834 | 0.02923 | 0.04597 | ukb-e-845_EAS |
| Arm predicted mass (left) | 14 | 94795202 | rs9989237 | T | C | 0.209988 | 454655 | -0.00308 | 0.001542 | 0.046 | ukb-b-9093 |
| Number of baps with butter/margarine | 14 | 94795202 | rs9989237 | T | C | 0.210572 | 64942 | 0.003736 | 0.001873 | 0.046 | ukb-b-13616 |
| Astigmatism angle (left) | 14 | 94795202 | rs9989237 | T | C | 0.210952 | 99071 | 0.010832 | 0.005433 | 0.046 | ukb-b-5090 |
| Unspecified urinary incontinence | 14 | 94795202 | rs9989237 | T | C | 0.2081 | NA | 0.0968 | 0.0485 | 0.04601 | finn-b-R18_UNSPE_URINARY_INCONTINENCE |
| OCT measured (right): Not performed - other reason | 14 | 94795202 | rs9989237 | T | C | 0.211457 | 54608 | 0.002899 | 0.001453 | 0.046061 | ukb-d-6070_7 |
| Treatment/medication code | 14 | 94795202 | rs9989237 | T | C | 0.7916 | NA | -0.2115 | 0.1061 | 0.04617 | ukb-e-20003_p13_AFR |
| CX3CL1 levels | 14 | 94795202 | rs9989237 | T | C | 0.2198 | 21758 | 0.0264 | 0.0132 | 0.04618 | ebi-a-GCST90012074 |
| TestASV_7 (Bacteroides) prevalence | 14 | 94795202 | rs9989237 | T | C | NA | 8956 | 0.074675 | 0.037457 | 0.046196 | ebi-a-GCST90011728 |
| Endometrial cancer | 14 | 94795202 | rs9989237 | T | C | 0.216708 | 121885 | 0.036637 | 0.018382 | 0.046252 | ebi-a-GCST006464 |
| Protein S100-A12 levels | 14 | 94795202 | rs9989237 | T | C | 0.235 | 1100 | 0.100024 | 0.049897 | 0.046276 | ebi-a-GCST90010341 |
| Time employed in main current job | 14 | 94795202 | rs9989237 | T | C | 0.5759 | NA | -0.0606 | 0.03041 | 0.04628 | ukb-e-757_p1_EAS |
| Vitamin B6 | 14 | 94795202 | rs9989237 | T | C | 0.6217 | NA | 0.08246 | 0.04139 | 0.04632 | ukb-e-100012_CSA |
| Current employment status | 14 | 94795202 | rs9989237 | T | C | 0.794 | NA | 0.2441 | 0.1226 | 0.04646 | ukb-e-6142_p7_AFR |
| CD45 on CD33+ HLA DR+ | 14 | 94795202 | rs9989237 | T | C | 0.2706 | 1580 | 0.08596 | 0.04314 | 0.04647 | ebi-a-GCST90002053 |
| Treatment/medication code: quinine | 14 | 94795202 | rs9989237 | T | C | 0.209385 | 337159 | -0.00049 | 0.000245 | 0.046489 | ukb-a-138 |
| Gas or solid-fuel cooking/heating | 14 | 94795202 | rs9989237 | T | C | 0.7961 | NA | 0.1215 | 0.06105 | 0.04662 | ukb-e-6139_p2_AFR |
| Pain in joint | 14 | 94795202 | rs9989237 | T | C | 0.6112 | NA | 0.1741 | 0.08751 | 0.04662 | ukb-e-745_CSA |
| Soft tissue disorders | 14 | 94795202 | rs9989237 | T | C | 0.2083 | NA | 0.0192 | 0.0096 | 0.04667 | finn-b-M13_SOFTTISSUE |
| Creatinine (enzymatic) in urine | 14 | 94795202 | rs9989237 | T | C | 0.7619 | NA | -0.07872 | 0.03957 | 0.04668 | ukb-e-30510_MID |
| OTU99_85 (Alistipes) abundance | 14 | 94795202 | rs9989237 | T | C | NA | 8956 | -0.15996 | 0.080425 | 0.046711 | ebi-a-GCST90011476 |
| Multiple coagulation factor deficiency protein 2 | 14 | 94795202 | rs9989237 | T | C | 0.21768 | 3301 | 0.0598 | 0.0301 | 0.046774 | prot-a-1867 |
| Elafin | 14 | 94795202 | rs9989237 | T | C | 0.21768 | 3301 | -0.0598 | 0.0301 | 0.046774 | prot-a-2265 |
| Endostatin | 14 | 94795202 | rs9989237 | T | C | 0.21768 | 3301 | -0.0596 | 0.0301 | 0.046774 | prot-a-617 |
| Cytochrome c oxidase subunit 6C | 14 | 94795202 | rs9989237 | T | C | 0.21768 | 3301 | -0.0597 | 0.0301 | 0.046774 | prot-a-639 |
| Epididymal secretory protein E1 | 14 | 94795202 | rs9989237 | T | C | 0.21768 | 3301 | -0.0598 | 0.0301 | 0.046774 | prot-a-2071 |
| a2009s lh S central thickness | 14 | 94795202 | rs9989237 | T | C | 0.21004 | 7916 | -0.0352 | 0.0176 | 0.046774 | ubm-a-3003 |
| NET100 0875 | 14 | 94795202 | rs9989237 | T | C | 0.21004 | 7916 | -0.0386 | 0.0194 | 0.046774 | ubm-a-2031 |
| a2009s lh S parieto occipital thickness | 14 | 94795202 | rs9989237 | T | C | 0.21004 | 7916 | -0.0343 | 0.0172 | 0.046774 | ubm-a-3023 |
| NET100 0193 | 14 | 94795202 | rs9989237 | T | C | 0.21004 | 7916 | 0.038 | 0.0191 | 0.046774 | ubm-a-1349 |
| NET25 0099 | 14 | 94795202 | rs9989237 | T | C | 0.21004 | 7916 | 0.0386 | 0.0194 | 0.046774 | ubm-a-1045 |
| NET100 1109 | 14 | 94795202 | rs9989237 | T | C | 0.21004 | 7916 | -0.038 | 0.0192 | 0.046774 | ubm-a-2265 |
| a2009s lh G temp sup-G T transv thickness | 14 | 94795202 | rs9989237 | T | C | 0.21004 | 7916 | -0.0362 | 0.0182 | 0.046774 | ubm-a-2991 |
| Prawns intake | 14 | 94795202 | rs9989237 | T | C | 0.6217 | NA | 0.02855 | 0.01436 | 0.04682 | ukb-e-103200_CSA |
| Job SOC coding: Management consultants, actuaries, economists and statisticians | 14 | 94795202 | rs9989237 | T | C | 0.208274 | 91149 | -0.00123 | 0.00062 | 0.046833 | ukb-d-22617_2423 |
| Own or rent accommodation lived in: Own with a mortgage | 14 | 94795202 | rs9989237 | T | C | 0.209407 | 356340 | 0.002439 | 0.001227 | 0.046839 | ukb-d-680_2 |
| Low calorie drink intake | 14 | 94795202 | rs9989237 | T | C | 0.7781 | NA | 0.1162 | 0.05847 | 0.04685 | ukb-e-100160_AFR |
| Non-cancer illness code, self-reported: tuberculosis (tb) | 14 | 94795202 | rs9989237 | T | C | 0.209974 | 462933 | -0.00036 | 0.000179 | 0.047 | ukb-b-15622 |
| Type of fat/oil used in cooking: Low fat polyunsaturated margarine | 14 | 94795202 | rs9989237 | T | C | 0.210565 | 64949 | 0.002537 | 0.001277 | 0.047 | ukb-b-12511 |
| Ever smoked | 14 | 94795202 | rs9989237 | T | C | NA | 44052 | 0.0081 | 0.0041 | 0.04709 | ieu-b-4857 |
| Human immunodeficiency virus [HIV] disease | 14 | 94795202 | rs9989237 | T | C | 0.2083 | NA | -0.1834 | 0.0924 | 0.04715 | finn-b-AB1_HIV |
| IgD+ B cell %Lymphocyte | 14 | 94795202 | rs9989237 | T | C | 0.2674 | 3657 | -0.05603 | 0.02826 | 0.04747 | ebi-a-GCST90001424 |
| Leg pain when walking normally | 14 | 94795202 | rs9989237 | T | C | 0.5888 | NA | -0.1327 | 0.06698 | 0.0475 | ukb-e-5485_CSA |
| Diabetic nephropathy | 14 | 94795202 | rs9989237 | T | C | 0.2083 | NA | -0.0624 | 0.0315 | 0.04778 | finn-b-DM_NEPHROPATHY |
| ADP-ribosyl cyclase/cyclic ADP-ribose hydrolase 1 | 14 | 94795202 | rs9989237 | T | C | 0.21768 | 3301 | 0.0596 | 0.0301 | 0.047863 | prot-a-443 |
| Inositol 1,4,5-trisphosphate receptor-interacting protein-like 1 | 14 | 94795202 | rs9989237 | T | C | 0.21768 | 3301 | -0.0596 | 0.0301 | 0.047863 | prot-a-1594 |
| Alcohol dehydrogenase 1B | 14 | 94795202 | rs9989237 | T | C | 0.21768 | 3301 | -0.0594 | 0.0301 | 0.047863 | prot-a-46 |
| Histone H2A deubiquitinase MYSM1 | 14 | 94795202 | rs9989237 | T | C | 0.21768 | 3301 | 0.0593 | 0.0301 | 0.047863 | prot-a-1983 |
| Cysteine-rich secretory protein LCCL domain-containing 2 | 14 | 94795202 | rs9989237 | T | C | 0.21768 | 3301 | -0.0596 | 0.0301 | 0.047863 | prot-a-663 |
| Glycolipid transfer protein domain-containing protein 2 | 14 | 94795202 | rs9989237 | T | C | 0.21768 | 3301 | -0.0593 | 0.0301 | 0.047863 | prot-a-1223 |
| Fibroblast growth factor 6 | 14 | 94795202 | rs9989237 | T | C | 0.21768 | 3301 | 0.0594 | 0.0301 | 0.047863 | prot-a-1096 |
| DKTatlas rh lateralorbitofrontal area | 14 | 94795202 | rs9989237 | T | C | 0.21004 | 7916 | 0.0257 | 0.013 | 0.047863 | ubm-a-2830 |
| DKTatlas lh transversetemporal thickness | 14 | 94795202 | rs9989237 | T | C | 0.21004 | 7916 | -0.0362 | 0.0182 | 0.047863 | ubm-a-2956 |
| IDP T1 FAST ROIs R mid temp gyrus tempocc | 14 | 94795202 | rs9989237 | T | C | 0.21004 | 7916 | 0.0327 | 0.0165 | 0.047863 | ubm-a-51 |
| a2009s rh G front inf-Orbital area | 14 | 94795202 | rs9989237 | T | C | 0.21004 | 7916 | -0.0349 | 0.0176 | 0.047863 | ubm-a-2865 |
| NET100 1420 | 14 | 94795202 | rs9989237 | T | C | 0.21004 | 7916 | 0.0383 | 0.0194 | 0.047863 | ubm-a-2576 |
| O_Burkholderiales abundance | 14 | 94795202 | rs9989237 | T | C | NA | 8956 | 0.037782 | 0.0191 | 0.04792 | ebi-a-GCST90011349 |
| Carcinoma in situ of breast, other/unspecified | 14 | 94795202 | rs9989237 | T | C | 0.2083 | NA | -0.2729 | 0.138 | 0.04794 | finn-b-CD2_INSITU_BREAST_NOS |
| Asymptomatic human immunodeficiency virus [HIV] infection status | 14 | 94795202 | rs9989237 | T | C | 0.2083 | NA | -0.3297 | 0.1667 | 0.04796 | finn-b-Z21_ASYMP_HUMAN_IMMUNODEFICIENCY_VIRUS_HIV_INFECTION_STATUS |
| 6mm strong meridian angle (right) | 14 | 94795202 | rs9989237 | T | C | 0.6112 | NA | 0.05113 | 0.02585 | 0.04797 | ukb-e-5106_CSA |
| Years since last cervical smear test | 14 | 94795202 | rs9989237 | T | C | 0.5886 | NA | 0.06369 | 0.03221 | 0.04801 | ukb-e-2704_EAS |
| Injury, poisoning and certain other consequences of external causes | 14 | 94795202 | rs9989237 | T | C | 0.209437 | 361194 | -0.00188 | 0.000951 | 0.048295 | ukb-d-XIX_INJURY_POISON |
| Leisure/social activities | 14 | 94795202 | rs9989237 | T | C | 0.7504 | NA | -0.2422 | 0.1227 | 0.048411 | ukb-e-6160_p6_MID |
| CD24+ CD27+ B cell Absolute Count | 14 | 94795202 | rs9989237 | T | C | 0.2674 | 3656 | -0.05689 | 0.02882 | 0.04846 | ebi-a-GCST90001418 |
| NA | 14 | 94795202 | rs9989237 | T | C | 0.6078 | NA | -0.1307 | 0.06629 | 0.048619 | ukb-e-recode54_CSA |
| Disorders of lens | 14 | 94795202 | rs9989237 | T | C | 0.209437 | 361194 | -0.00119 | 0.000603 | 0.048737 | ukb-d-H7_LENS |
| "Alzheimer<c3><95>s disease, wide definition" (more controls excluded) | 14 | 94795202 | rs9989237 | T | C | 0.207 | NA | 0.056 | 0.0284 | 0.04876 | finn-b-G6_AD_WIDE_EXMORE |
| PM: final answer | 14 | 94795202 | rs9989237 | T | C | 0.6278 | NA | 0.1095 | 0.05563 | 0.04892 | ukb-e-4293_p3_CSA |
| Cysteine-rich secretory protein 3 | 14 | 94795202 | rs9989237 | T | C | 0.21768 | 3301 | 0.059 | 0.0301 | 0.048978 | prot-a-662 |
| Histidine triad nucleotide-binding protein 1 | 14 | 94795202 | rs9989237 | T | C | 0.21768 | 3301 | -0.0593 | 0.0301 | 0.048978 | prot-a-1338 |
| Non-histone chromosomal protein HMG-14 | 14 | 94795202 | rs9989237 | T | C | 0.21768 | 3301 | -0.0593 | 0.0301 | 0.048978 | prot-a-1357 |
| 2'-5'-oligoadenylate synthase 1 | 14 | 94795202 | rs9989237 | T | C | 0.21768 | 3301 | 0.0592 | 0.0301 | 0.048978 | prot-a-2136 |
| Kallikrein-8 | 14 | 94795202 | rs9989237 | T | C | 0.21768 | 3301 | -0.059 | 0.0301 | 0.048978 | prot-a-1667 |
| Transmembrane protein 40 | 14 | 94795202 | rs9989237 | T | C | 0.21768 | 3301 | 0.0592 | 0.03 | 0.048978 | prot-a-3010 |
| NET100 0139 | 14 | 94795202 | rs9989237 | T | C | 0.21004 | 7916 | -0.0381 | 0.0193 | 0.048978 | ubm-a-1295 |
| NET100 0356 | 14 | 94795202 | rs9989237 | T | C | 0.21004 | 7916 | 0.0378 | 0.0192 | 0.048978 | ubm-a-1512 |
| NET100 0125 | 14 | 94795202 | rs9989237 | T | C | 0.21004 | 7916 | 0.0376 | 0.019 | 0.048978 | ubm-a-1281 |
| IDP dMRI ProbtrackX MO ptr r | 14 | 94795202 | rs9989237 | T | C | 0.21004 | 7916 | -0.0375 | 0.019 | 0.048978 | ubm-a-702 |
| Total lipids in large LDL | 14 | 94795202 | rs9989237 | T | C | 0.20942 | NA | -0.00931 | 0.005039 | 0.049 | met-d-L_LDL_L |
| Types of spreads/sauces consumed: Tomato ketchup | 14 | 94795202 | rs9989237 | T | C | 0.210565 | 64949 | 0.002667 | 0.001354 | 0.049 | ukb-b-260 |
| Duration to complete numeric path (trail #1) | 14 | 94795202 | rs9989237 | T | C | 0.210132 | 99479 | -0.01079 | 0.005472 | 0.049 | ukb-b-16026 |
| PCT responsible for patient data: HAMPSHIRE PCT | 14 | 94795202 | rs9989237 | T | C | 0.209969 | 463010 | -0.00037 | 0.000187 | 0.049 | ukb-b-5693 |
| Operative procedures - secondary OPCS: Y98.2 Radiology of two body areas | 14 | 94795202 | rs9989237 | T | C | 0.209969 | 463010 | -0.00045 | 0.000228 | 0.049 | ukb-b-3686 |
| Non-cancer illness code, self-reported: endometriosis | 14 | 94795202 | rs9989237 | T | C | 0.209974 | 462933 | -0.00045 | 0.00023 | 0.049 | ukb-b-10903 |
| Pain type(s) experienced in last month | 14 | 94795202 | rs9989237 | T | C | 0.5836 | NA | 0.1312 | 0.06666 | 0.04902 | ukb-e-6159_p1_EAS |
| C-type lectin domain family 5 member A levels | 14 | 94795202 | rs9989237 | T | C | 0.235 | 1313 | 0.09192 | 0.046626 | 0.049034 | ebi-a-GCST90010298 |
| Creatinine | 14 | 94795202 | rs9989237 | T | C | 0.20968 | 344104 | 0.00468 | 0.002378 | 0.049119 | ukb-d-30700_irnt |
| Drive faster than motorway speed limit | 14 | 94795202 | rs9989237 | T | C | 0.209385 | 306030 | 0.004912 | 0.002499 | 0.049324 | ukb-a-8 |
| Alcoholic liver disease (K11_ALCOLIV) | 14 | 94795202 | rs9989237 | T | C | 0.2082 | NA | 0.0917 | 0.0467 | 0.04949 | finn-b-K11_ALCOLIV |
| SSC-A on plasmacytoid Dendritic Cell | 14 | 94795202 | rs9989237 | T | C | 0.2688 | 2872 | 0.06292 | 0.03202 | 0.04949 | ebi-a-GCST90002072 |
| C_Betaproteobacteria abundance | 14 | 94795202 | rs9989237 | T | C | NA | 8956 | 0.037478 | 0.019103 | 0.049771 | ebi-a-GCST90011304 |
| Non-cancer illness code, self-reported | 14 | 94795202 | rs9989237 | T | C | 0.6077 | NA | -0.1281 | 0.06532 | 0.04984 | ukb-e-20002_p62_CSA |
| TNF receptor superfamily member 1B | 14 | 94795202 | rs9989237 | T | C | 0.2384 | 3394 | 0.0616 | 0.0314 | 0.04988 | prot-b-42 |
| Fibroblast growth factor 5 levels | 14 | 94795202 | rs9989237 | T | C | NA | 982 | -0.108 | 0.0551 | 0.04999 | ebi-a-GCST90000469 |
| NA | 14 | 94795202 | rs9989237 | T | C | 0.6111 | NA | 0.1543 | 0.07873 | 0.04999 | ukb-e-recode403_CSA |

Note, only values with a p-value < 0.05 are presented in this table

# **References**

1. Warrington NM, Beaumont RN, Horikoshi M, Day FR, Helgeland Ø, Laurin C, Bacelis J, Peng S, Hao K, Feenstra B *et al*: Maternal and fetal genetic effects on birth weight and their relevance to cardio-metabolic risk factors. Nat Genet 2019, 51(5):804-814.

2. Warrington NM, Freathy RM, Neale MC, Evans DM: Using structural equation modelling to jointly estimate maternal and fetal effects on birthweight in the UK Biobank. Int J Epidemiol 2018.

3. Hewitt J, Walters M, Padmanabhan S, Dawson J: Cohort profile of the UK Biobank: diagnosis and characteristics of cerebrovascular disease. BMJ Open 2016, 6(3).

4. Bycroft C, Freeman C, Petkova D, Band G, Elliott LT, Sharp K, Motyer A, Vukcevic D, Delaneau O, O’Connell J *et al*: The UK Biobank resource with deep phenotyping and genomic data. Nature 2018, 562(7726):203-209.

5. **Important note about imputed genetics data** [<http://www.ukbiobank.ac.uk/2017/07/important-note-about-imputed-genetics-data/>]

6. Loh P-R, Tucker G, Bulik-Sullivan BK, Vilhjálmsson BJ, Finucane HK, Salem RM, Chasman DI, Ridker PM, Neale BM, Berger B *et al*: Efficient Bayesian mixed-model analysis increases association power in large cohorts. Nat Genet 2015, 47(3):284-290.

7. Knight B, Shields BM, Hattersley AT: The Exeter Family Study of Childhood Health (EFSOCH): study protocol and methodology. Paediatr Perinat Epidemiol 2006, 20(2):172-179.

8. Abraham G, Inouye M: Fast Principal Component Analysis of Large-Scale Genome-Wide Data. PLOS ONE 2014, 9(4):e93766.

9. Crawford AA, Bankier S, Altmaier E, Barnes CLK, Clark DW, Ermel R, Friedrich N, van der Harst P, Joshi PK, Karhunen V *et al*: Variation in the SERPINA6/SERPINA1 locus alters morning plasma cortisol, hepatic corticosteroid binding globulin expression, gene expression in peripheral tissues, and risk of cardiovascular disease. J Hum Genet 2021, 66(6):625-636.

10. Bolton JL, Hayward C, Direk N, Lewis JG, Hammond GL, Hill LA, Anderson A, Huffman J, Wilson JF, Campbell H *et al*: Genome Wide Association Identifies Common Variants at the SERPINA6/SERPINA1 Locus Influencing Plasma Cortisol and Corticosteroid Binding Globulin. PLoS Genet 2014, 10(7):e1004474.
